# Towards an Accurate and Precise Chronology for the Colonization of Australia: The Example of Riwi, Kimberley, Western Australia

**DOI:** 10.1371/journal.pone.0160123

**Published:** 2016-09-21

**Authors:** Rachel Wood, Zenobia Jacobs, Dorcas Vannieuwenhuyse, Jane Balme, Sue O’Connor, Rose Whitau

**Affiliations:** 1 Research School of Earth Sciences, Australian National University, Canberra, ACT, 2601, Australia; 2 Centre for Archaeological Science, School of Earth and Environmental Sciences, University of Wollongong, New South Wales, 2522, Australia; 3 School of Social Sciences, University of Western Australia, Crawley, Western Australia, 6009, Australia; 4 Department of Archaeology and Natural History, Research School of Pacific and Asian Studies, Australian National University, Canberra, ACT, 2601, Australia; University of Ferrara, ITALY

## Abstract

An extensive series of 44 radiocarbon (^14^C) and 37 optically stimulated luminescence (OSL) ages have been obtained from the site of Riwi, south central Kimberley (NW Australia). As one of the earliest known Pleistocene sites in Australia, with archaeologically sterile sediment beneath deposits containing occupation, the chronology of the site is important in renewed debates surrounding the colonization of Sahul. Charcoal is preserved throughout the sequence and within multiple discrete hearth features. Prior to ^14^C dating, charcoal has been pretreated with both acid-base-acid (ABA) and acid base oxidation-stepped combustion (ABOx-SC) methods at multiple laboratories. Ages are consistent between laboratories and also between the two pretreatment methods, suggesting that contamination is easily removed from charcoal at Riwi and the Pleistocene ages are likely to be accurate. Whilst some charcoal samples recovered from outside hearth features are identified as outliers within a Bayesian model, all ages on charcoal within hearth features are consistent with stratigraphy. OSL dating has been undertaken using single quartz grains from the sandy matrix. The majority of samples show D_e_ distributions that are well-bleached but that also include evidence for mixing as a result of post-depositional bioturbation of the sediment. The results of the two techniques are compared and evaluated within a Bayesian model. Consistency between the two methods is good, and we demonstrate human occupation at this site from 46.4–44.6 cal kBP (95.4% probability range). Importantly, the lowest archaeological horizon at Riwi is underlain by sterile sediments which have been dated by OSL making it possible to demonstrate the absence of human occupation for between 0.9–5.2 ka (68.2% probability range) prior to occupation.

## 1. Introduction

### 1.1 Dating the colonization of Australia

The time of arrival of early modern humans in Australia is an open question, in large part because most sites do not contain human fossils. However, an early colonisation is made possible by recent discoveries of *Homo sapiens* fossils dating to more than 40,000 years in southern China [1] and Laos [2, 3], alongside increasing archaeological evidence for an earlier migration out of Africa than previously thought [4–6]. Moreover, genetic clock estimates for the founding populations in Australia have been estimated variably to be 63 ± 5 ka [7], 58 ± 8 ka [8], and 55 ± 11 ka [9] and most recently, Rasmussen, Guo [10] provided genetic evidence that Aboriginal Australians descended from an early Asian expansion wave some 62–75 ka ago.

In Australia, the oldest human fossils are about 40,000 years old [11, 12]. Earlier archaeological traces are restricted to artefacts buried 45,000–60,000 years ago [13–19]. However precision on age estimates is often in the order of at least ± 5 ka (at 1σ), making interpretation of these early age estimates difficult. Recent reviews of the chronology of Australian sites coalesce on an age of no later than 47–48 ka ago [20–22], although many researchers still consider ages of 50 ka or earlier to be probable [16, 23, 24].

At the heart of the Australian colonization debate lies questions about (a) the accuracy of the ages obtained using ^14^C and luminescence-based dating techniques, (b) the precision of the age estimates and their usefulness in pinpointing the earliest occupation of the continent, and (c) the reliability of the context and association of the artefacts with the dated materials. The debate is currently marred by inappropriate consideration of uncertainties, low numbers of dates hindering application of statistical analyses, and a lack of new systematic dating studies using the latest dating procedures at the oldest sites.

The aim of this study is to test the accuracy of ^14^C and single grain OSL ages at Riwi Cave. High resolution sampling allowed Bayesian chronologies to be created for each method and then compared. This should establish a benchmark for future within- and between-site comparisons to facilitate improved integration of ages and their associated uncertainties into further debates about the earliest occupation of Australia. Riwi Cave provides an excellent testing ground since it is one of only a few ‘old’ Australian sites that contain intact Pleistocene hearth features in a sandy matrix, ideal for both ^14^C and OSL dating.

### 1.2 Riwi setting, stratigraphy and archaeological context

**Fig 1:**
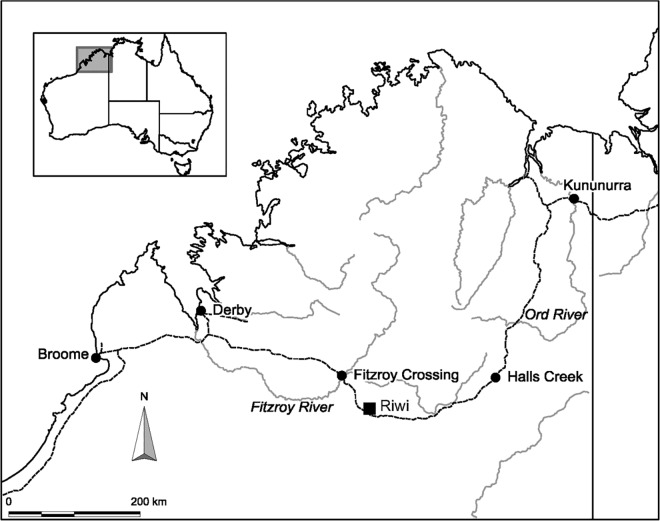
Location of Riwi Cave.

**Fig 2:**
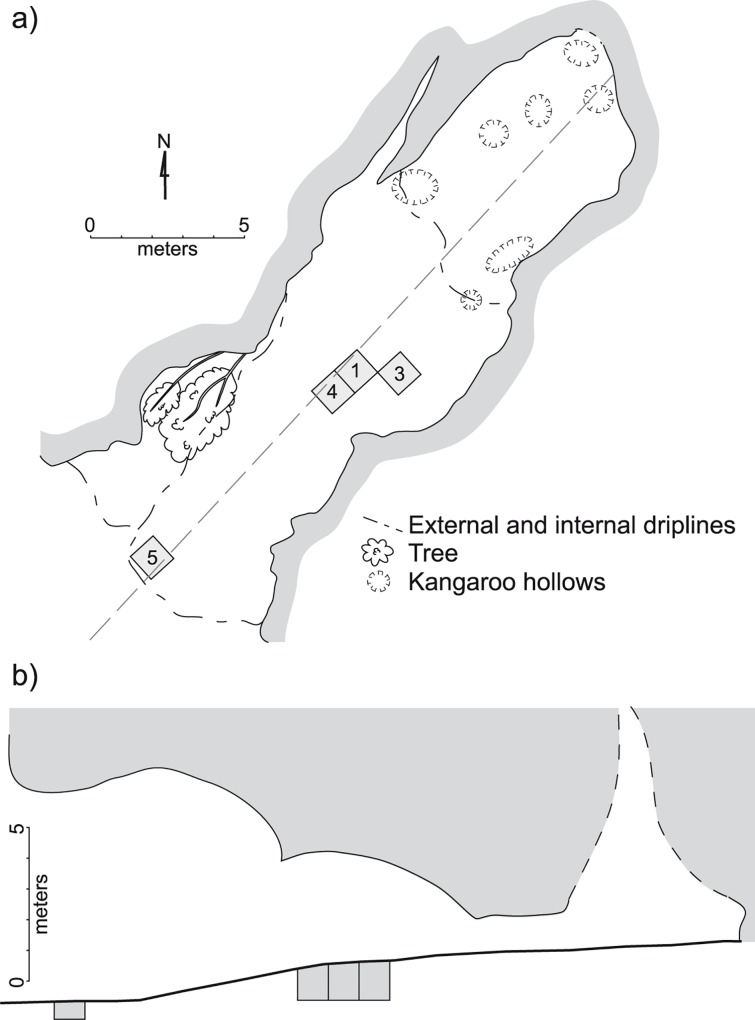
Riwi cave. (A) Plan and (B) profile. CAD by Dorcas Vannieuwenhuyse.

Riwi is situated on Gooniyandi traditional land in the south central Kimberley region of Western Australia (-18.69; 126.06) (Fig 1). It is a large (~146 m^2^), deep and high cave cut into the base of the west-facing Lawford Range composed of Devonian limestone (Fig 2). In 1999, a 1 m wide and 50 cm deep test trench was excavated; in one quadrant, excavation proceeded a further 50 cm in depth [15]. Excavation occurred in 5–10 cm spits, except where guided by large changes in stratigraphy, and all sediment was passed through 5 and 2 mm sieves. In 2013, the site was revisited with a permit provided by the Department of Aboriginal Affairs (Section 16 permit no. 499). The remaining quadrants of the original 1 m^2^ test pit (Square 1) were excavated to bedrock and the excavations were expanded by an additional three 1 m^2^ test pits (Squares 3, 4 and 5) (Fig 2A and 2B). In squares 1, 3 and 4, located in the center of the main cave chamber, bedrock was reached at between 100 cm and 130 cm (in most places circa 117 cm). Square 5, located at the mouth of the cave, was excavated to a maximum depth of 65 cm, when decomposing bedrock was reached. All squares were excavated in arbitrary excavation units of 2 cm thickness, and in 50 cm horizontal quadrants, and some features, such as pits and hearths, were removed separately. Excavation of archaeologically sterile sediments near bedrock was in 3–5 cm arbitrary units. All excavated materials were dry sieved through nested 5 and 1.5 mm mesh screens.

**Fig 3:**
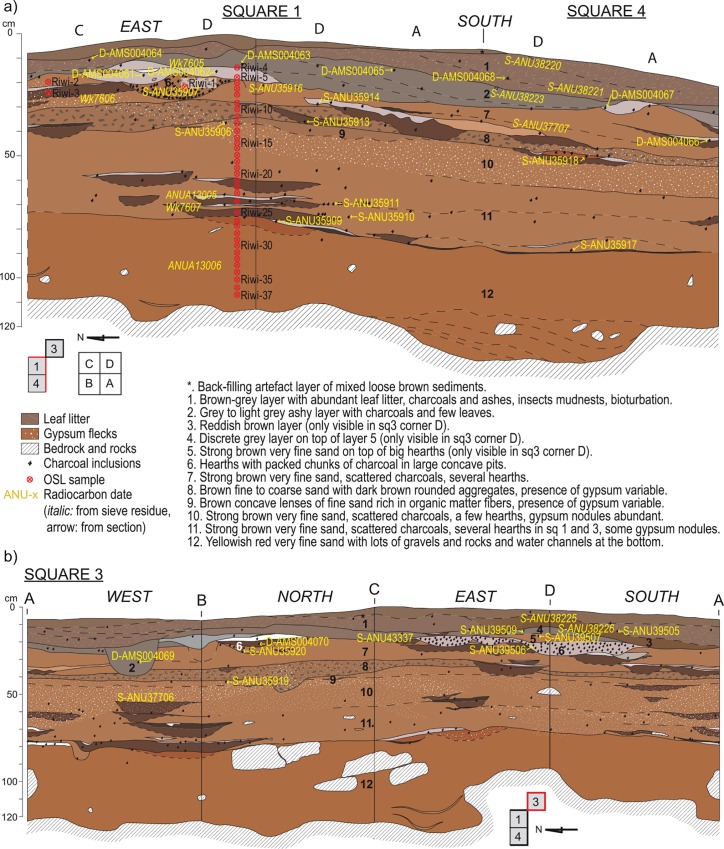
**Stratigraphic profile and description of (A) squares 1 and 4, and (B) square 3.** The location of OSL samples is marked in red, and the location of radiocarbon charcoal samples in yellow. Charcoal samples recovered from the sieve are given in italics, and charcoal samples taken from the section are marked with an arrow. CAD by Dorcas Vannieuwenhuyse.

The stratigraphic sequence observed in squares 1–4 and 3 was divided into twelve stratigraphic units (SUs) (Fig 3A and 3B) that can be grouped in two visually distinct levels: SUs 12–3, dominated by strong brown sediments and top units SUs2-1, composed of grey-ashy deposits. The strong brown sediments are mainly aeolian in origin with generally more than 50% of the <2 mm fraction composed of very fine sands (63–125 μm). The rest of the geogenic deposits are composed of fine to coarser sands derived from the in-situ chemical weathering and physical breakdown of sandstone bedrock and cave walls, especially near the bottom of the deposit.

The deepest recognizable hearth is at the interface of SU11 and 12. Other clear and *in situ* hearths are found in SUs 3, 6, middle and base of 7, top of 10, middle of 11 and top of 12 (Fig 3A and 3B). Artefact numbers drop at the transition between SU12 and SU11. Only 16 stone artefacts have been recovered below this level. Most were from within 10 cm of the transition to SU12 and only three were found between 10 and 20 cm beneath the deepest hearth. All were recovered from Square 4 quad A (N = 4) and quad B (N = 12), where the boundary between SU12 and SU11 was slightly undulating and where excavation units may have inadvertently cut between the two SUs. At the top of this package of strong brown sediments is a conspicuous brown sediment layer (SU3), found in Square 3, at the interface of the underlying red-brown and overlying grey-ashy sediment.

SUs 2 and 1 represent the grey deposits at the top of the sequence. The sediment is dominated by anthropogenic (ash, charcoal) and biogenic (macrobotanics, coprolites) remains mixed with a smaller proportion of geogenic sediment. The top part of the sequence has suffered from more intense trampling, bioturbation, and erosional post-depositional processes than the strong brown sequence beneath. SU2 and SU1 are richer in lithic artefacts than the deeper strong brown sediments, and numerous types of organic materials (charcoal, seeds, nuts, fruit, paper bark fragments and wood shavings) are preserved [25].

### 1.3 Previous chronology

Using six ^14^C ages on charcoal from the initial excavation, Balme [15] suggested that the upper package of sediments was Holocene and the deeper strong brown sequence Pleistocene (Table 1). Major age gaps were recognized in the sequence. Notably, deposits with ages associated with the last glacial maximum (LGM) were absent leading to speculation that humans were not present in the region, located within the arid zone during this period [15, 26, 27]. Furthermore, a sample collected from a hearth prepared using acid-base-oxidation with stepped combustion (ABOx-SC) [28] gave an age of 46890–43040 cal BP at 95.4% probability (41,300 ± 1020 BP, ANUA-13005) [15, 29], one of the earliest dates on a sample directly associated with human activity in Australia.

**Table 1:** Published dates from Riwi [15].

SU	Context	Feature	Pretreatment	Lab code	Radiocarbon date (BP)	Calibrated date (cal BP, 95.4% probability range)	
						from	to
2top	SQ1-XU3-10cm	Isolated charcoal	ABA	Wk-7605	5290 ± 60	6200	5910
6	SQ1-XU5-20cm	Charcoal lens	ABA	Wk-7896	29550 ± 290	34200	33040
7mid	SQ1-XU7-25cm	Hearth	ABA	Wk-7606	31860 ± 450	36730	34770
11 mid	SQ1-XU13-65cm	Hearth	ABOx-SC	ANUA-13005	41300 ± 1020	46890	43040
11 base	SQ1-XU14-70cm	Hearth	ABA	Wk-7607	>40000		
12 mid	SQ1-XU16-95cm	Isolated charcoal	ABOx-SC	ANUA-13006	40700 ± 1260	47070	42430

Radiocarbon dates have been calibrated against SHCal13 [30] in OxCal v.4.2 [31]

## 2. Age Determination by ^14^C Dating

### 2.1 Sample collection, pretreatment and measurement

In this study 33 charcoal fragments have been dated by two laboratories—the Australian National University Single Stage Accelerator (SANU-) and Direct AMS (DAMS-). One sample from a wooden artefact, most likely a boomerang [25, 32] has also been dated at the SANU. Charcoal fragments from hearth features were targeted, but were supplemented by fragments from the matrix, not necessarily associated with any particular archaeological features. The majority of samples were taken from the section wall, providing excellent stratigraphic control, whilst the remaining samples were either sampled *in situ* during excavation or recovered from the 5 mm sieve (Table 2).

**Table 2:** New radiocarbon dates from Riwi Cave.

SU	Context	Feature	Collection method	Genus	%C	Pre-treatment	Lab ID	^14^C date (BP)	Error (1σ)
1 top	SQ4-XU1-QD		5mm sieve	*Corymbia* sp. (Type R01)	62	ABA	SANU-38220	18930	50
1 mid	SQ4-SOUTH-24	scattered in leafy layer	wall section 2012	Not determined		ABA	D-AMS004068	816	27
1 mid	SQ1-EAST-34	scattered charcoal	wall section 2012	Not determined		ABA	D-AMS004064	956	29
1 base	SQ3 XU8 QC			*Grevillea/Hakea* sp.	42	Holo-cellulose	SANU-43337	670	20
SU1/3	SQ3-SOUTH-26	scattered	wall section 2012	*Corymbia* sp. (Type R01)	68	ABA	SANU-39505	6385	30
SU1/2	SQ4-XU5-QD	scattered top grey layer	5mm sieve residue	*Mallotus* sp.	59	ABA	SANU-38221	6445	30
2 pit	SQ3-WEST-21	scattered in pit cutting Pleistocene layer	wall section 2012	Not determined		ABA	D-AMS004069	6179	29
2 top	SQ1-SOUTH-36	scattered charcoal	wall section 2012	Not determined		ABA	D-AMS004065	6206	37
2 top	SQ4-XU8-QD	Scattered at base of grey layer	5mm sieve residue	*Corymbia* sp. (Type R01)	56	ABA	SANU-38223	6245	30
2 top	SQ1-EAST-32	scattered charcoal in ashy layer	wall section 2012	Not determined		ABA	D-AMS004063	6384	32
2base	SQ1-EAST-26	ashy layer (hearth?)	wall section 2012	Not determined		ABA	D-AMS004061	6250	35
2base	SQ1-EAST-28	ashy layer (hearth?)	wall section 2012	Not determined		ABA	D-AMS004062	6315	32
2base	SQ4-SOUTH-20	scattered at top of grey layer	wall section 2012	Not determined		ABA	D-AMS004067	6452	34
3	SQ3-XU3-QD	Hearth feature 1?	5mm sieve	*Corymbia* sp. (Type R01)	55	ABA	SANU-38225	18390	60
3	SQ3-XU5-QD-Feature 1	hearth feature 1	5mm sieve	*Corymbia* sp. (Type R01)	56	ABA	SANU-38226	16930	50
					60	ABA	SANU-38814	16850	100
4	SQ3-EAST-32	scattered grey layer	wall section 2012	Not determined	53	ABA	SANU-39509	27280	160
5	SQ3-EAST-30	scattered red layer	wall section 2012	*Corymbia* sp. (Type R01)	66	ABA	SANU-39507	30690	220
6	SQ3-EAST-28	hearth	wall section 2012	*Corymbia* sp. (Type R01)	63	ABA	SANU-39506	29050	180
					62	ABA	SANU-39510	29350	190
6	SQ1-EAST-20	no details	wall section 2012	*Corymbia* sp. (Type R01)	69	ABOx-SC	SANU-35907	29790	190
6	SQ3-NORTH-18	hearth	wall section 2012	*Corymbia* sp. (Type R01)	59	ABOx-SC	SANU-35920	30110	200
6	SQ3-NORTH-22	hearth	wall section 2012	Not determined		ABA	D-AMS004070	30154	141
7 top	SQ1-SOUTH-32	scattered charcoal	wall section 2012	Unidentifiable	51	ABOx-SC	SANU-35916	29720	190
7mid	SQ4-SOUTH-17	hearth	wall section 2012	Not determined		ABA	D-AMS004066	31888	153
7base	SQ1-SOUTH-29	hearth	wall section 2012	*Corymbia* sp. (Type R01)	58	ABOx-SC	SANU-35914	33000	280
					54	ABOx-SC	SANU-35921	33270	280
					70	ABA	SANU-35924	32910	270
7 base	SQ4-XU12-QD	no details	excavation, in situ sample	*Myrtaceae* sp.	58	ABOx-SC	SANU-37707	34450	340
9 top	SQ1-SOUTH-25	scattered charcoal/hearth	wall section 2012	*Corymbia* sp. (Type R01)	60	ABOx-SC	SANU-35913	33850	300
9	SQ1-EAST-16	scattered charcoal	wall section 2012	*Corymbia* sp. (Type R01)	75	ABOx-SC	SANU-35906	33560	300
9	SQ3-NORTH-14	scattered charcoal	wall section 2012	Unidentifiable	68	ABOx-SC	SANU-35919	34000	310
10 top	SQ4-SOUTH-12	hearth	wall section 2012	*Corymbia* sp. (Type R01)	73	ABOx-SC	SANU-35918	33340	280
10 mid	SQ3-XU19-QB	scattered charcoal	excavation, in situ sample	*Corymbia* sp. (Type R01)	53	ABOx-SC	SANU-37706	36680	420
11 mid	SQ1-SOUTH-12	hearth	wall section 2012	cf. *Corymbia* sp.	71	ABOx-SC	SANU-35911	41520	750
					79	ABOx-SC	SANU-35922	41690	760
11	SQ1-SOUTH-9	scattered charcoal	wall section 2012	Unidentifiable	62	ABOx-SC	SANU-35910	29840	190
12 top	SQ1-SOUTH-2	hearth	wall section 2012	*Corymbia* sp. (Type R01)	69	ABOx-SC	SANU-35909	41590	760
12 top	SQ4-SOUTH-1	charcoal lens	wall section 2012	Unidentifiable	63	ABOx-SC	SANU-35917	42140	810
					68	ABA	SANU-35925	41050	710

Radiocarbon dates have been calibrated against SHCal13 in OxCal v.4.2 [30, 31].

An attempt was made to identify all charcoal samples dated at the SANU to genus level [33] (Table 2). At the SANU, all samples expected to be Pleistocene in age and larger than 50 mg were subjected to ABOx-SC pretreatment [28]. After physical cleaning and crushing to <1 mm, charcoal was washed in acid (HCl, 6M, room temperature, 1 hr), alkali (NaOH, 2M, room temperature, 30 min, replaced until solution was colourless) and an oxidizing solution (2M H_2_SO_4_, 0.1M KCr_2_O_7_, 60°C, 20 hr), rinsing with MilliQ^TM^ water after each treatment. Up to 20 mg of the freeze-dried product was then pre-combusted in oxygen (ultra-high purity, 1 atm, 600°C, 2 hrs).

The more gentle acid-base-acid (ABA) treatment was applied to samples of expected Holocene age, samples smaller than 50 mg, and samples which failed the ABOx-SC treatment (Table 2). At some sites, an ABA pretreatment is not able remove sufficient young contaminants from Pleistocene-aged charcoal to produce an accurate age estimate [18, 28, 34–36]. To test whether it was appropriate to apply a more gentle treatment to charcoal from Riwi, two samples that survived the ABOx-SC treatment were also dated with an ABA protocol. This consisted of physical cleaning and crushing followed by washes in acid (HCl, 1M, 80°C, 30 min), alkali (NaOH, 1M, 80°C, 1 hr, replaced until solution was colourless) and acid (HCl, 1M, 80°C, 30 min), rinsing with MilliQ^TM^ water three times after each treatment or until the solution remained colourless. After the surface was physically removed, ~10 mg of the wooden artefact was also treated with the ABA protocol described for charcoal, followed by a bleaching step (1M NaClO_2_: 1M HCl, room temperature, 15 min).

Charcoal and wood remaining after these treatments was combusted in an evacuated quartz tube (900°C, 6 hrs, in the presence of CuO wire and Ag foil), and the CO_2_ generated was collected and purified cryogenically before graphitization over an Fe catalyst in the presence of H_2_ prior to measurement in a NEC single stage Accelerator Mass Spectrometer (AMS) at the SANU [37]. Ages have been calculated according to Stuiver and Polach [38] using a δ^13^C value measured by AMS. Samples were treated alongside a pretreatment blank of fossil wood charred at 550°C [34] or coal. A background has been subtracted from each sample based on long term repeat measurements of coal pretreated with ABA. The smaller number of ABOx-SC treated blanks are consistent with this correction. Three samples have been pretreated and dated twice as part of routine laboratory quality assurance protocols.

Charcoal dated at Direct AMS was pretreated with an ABA protocol, modified according to the preservation of each sample. Samples were graphitized and dated by AMS. Dates have been calculated according to Stuiver and Polach [38] using a δ^13^C value measured by AMS.

### 2.2 Results

Preservation of charcoal within the Pleistocene levels at Riwi was generally poor. Of 38 samples submitted to the SANU laboratory, 15 found in units dating to the Pleistocene dissolved during the ABA and/or ABOx-SC chemical pretreatment. Despite this, the ^14^C ages appear accurate and are generally consistent with stratigraphy (Table 2). Those samples to survive the pretreatment had high carbon contents (>50%), suggesting that contamination from, for example, sediment inclusions [39] was absent. Where individual charcoal fragments were pretreated in the same way twice, the results are identical, with Chi-squared tests at p<0.05 (Table 2). Moreover, results are also identical where single charcoal fragments were dated with both ABA and ABOx-SC protocols (Table 2). This suggests that the charcoal at Riwi is not grossly contaminated, a conclusion supported by the general consistency seen between ^14^C ages on different fragments of charcoal from the same context produced at four different laboratories (SANU, ANUA, DAMS and Wk) using a range of different ABA pretreatment protocols.

## 3. Age Determination by OSL Dating

### 3.1 Sampling for OSL dating

37 samples were collected for OSL dating using small (~2 cm diameter, 10 cm long) stainless steel tubes. The majority of samples were taken from a continuous column from the eastern profile of Square 1. A further 3 samples were taken from units not present in this column (SU6 and 7) (Fig 3A).

### 3.2 Single grain OSL measurement and analysis

#### 3.2.1 Extraction of quartz

Sample tubes were opened under dim red light and quartz grains extracted using standard preparation procedures [40]. Carbonates were dissolved in 10% HCl, and organic matter oxidised in 30% H_2_O_2_. The remaining sample was dried and sieved to isolate grains of 180–212 μm in diameter, and feldspar, quartz and heavy minerals separated by density using sodium polytungstate solutions of 2.62 and 2.70 specific gravities, respectively. Quartz grains were etched with HF (48%, 40 min) to remove the alpha-irradiated rind of quartz grains and destroy any remaining feldspars, rinsed in HCl to remove precipitated fluorides, dried and sieved. Grains retained on the 180 μm diameter mesh were used for dating.

#### 3.2.2 Measurement

Risø single grain Al discs [41] were used for measurement of the 180–212 μm grains. Each disk was examined under a microscope after measurement to check that only one grain was present in each hole. All measurements were made in an identical manner and with the same equipment, using the single aliquot regenerative-dose (SAR) procedure described elsewhere (e.g. [42, 43]). The SAR procedure involves measuring the OSL signals from the natural (burial) dose (L_N_) and from a series of regenerative doses (L_x_; given in the laboratory by means of a calibrated ^90^Sr/^90^Y beta source). Grains from Holocene-age sediments were given successive regenerative doses of 10, 20, 40, 80, 0 and 10 Gy and grains from Pleistocene-age sediments doses of 40, 80, 160, 240, 0 and 40 Gy. Each regenerative dose is preheated prior to optical stimulation by an intense, green (532 nm) laser beam for 2 s at 125°C. The resulting ultraviolet OSL emissions were detected by an Electron Tubes Ltd 9235QA photomultiplier tube fitted with Hoya U-340 filters. A fixed test dose of ~11 Gy, was given after each natural and regenerative dose, and the induced OSL signals (T_N_ or T_x_) used to correct for any sensitivity changes during the SAR sequence. A duplicate regenerative dose was included in the procedure, to check on the adequacy of this sensitivity correction, and a ‘zero dose’ measurement made to monitor the extent of any ‘recuperation’ induced by the preheat. As a check on possible contamination of the etched quartz grains by feldspar inclusions, we also applied the OSL IR depletion-ratio test [44] to each grain at the end of the SAR sequence, using an infrared exposure of 40 s at 50°C.

L_x_ and T_x_ values were estimated from the first 0.22 s of OSL decay with the mean count recorded over the last 0.3 s subtracted as background. Sensitivity-corrected (L_x_/T_x_) dose response curves were constructed from these induced L_x_ and T_x_ OSL signals, using a single saturating-exponential function of the form *I* = *I*_*0*_(1-exp^-D/D0^). In this function, *I* is the L_x_/T_x_ value at regenerative dose *D*, *I*_*0*_ is the saturation value of the exponential curve and *D*_*0*_ is the characteristic saturation dose; *I*_*0*_ and *D*_*0*_ are estimated from the data. The sensitivity-corrected natural OSL signal (L_N_/T_N_) was projected onto the fitted dose-response curve to obtain the D_e_ by interpolation. The uncertainty on the D_e_ estimate of each grain (from photon counting statistics, curve fitting uncertainties, and an allowance of 2% per OSL measurement for instrument irreproducibility) was determined by Monte Carlo simulation, using the procedures described by Duller [45]. The final age uncertainty also includes a further 2% to allow for any bias in the beta source calibration; this error is added as a systematic uncertainty.

#### 3.2.3 Dose recovery tests

**Fig 4:**
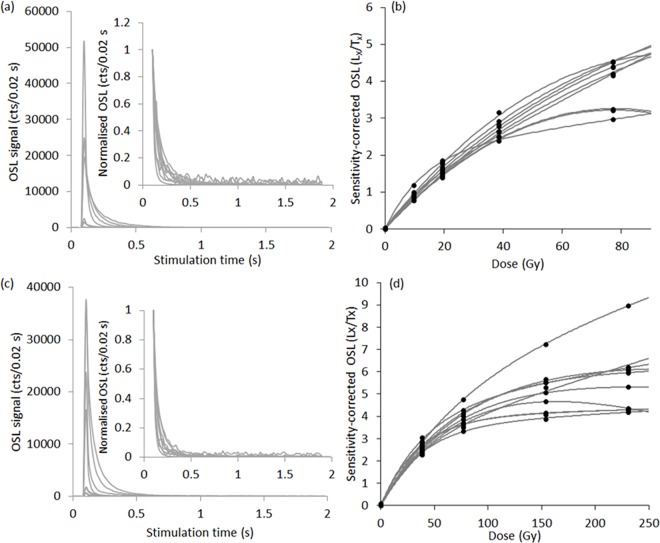
The OSL decay curves for representative samples of grains from (a) Riwi-4 and (c) Riwi-27 that span the range of luminescence sensitivities (‘relative brightness’). (b) and (d) show the corresponding dose response curves for those grains shown in (a) and (c).

A number of dose recovery tests were conducted on one sample (Riwi-22) to determine the optimum preheat temperatures. All grains were exposed to natural sunlight for two days to empty the electron traps, and were then given a known laboratory dose (~70 Gy). Five preheat (PH) combinations were tried, where PH-1 is that prior to measurement of L_N_ and L_x_ and PH-2 that prior to measurement of T_N_ and T_x_. The combinations included: (1) PH-1 = 160°C for 10 s; PH-2 = 160°C for 5 s, (2) PH-1 = 180°C for 10 s; PH-2 = 180°C for 5 s, (3) PH-1 = 220°C for 10 s; PH-2 = 160°C for 5 s, (4) PH-1 = 260°C for 10 s; PH-2 = 160°C for 5 s, and (5) PH-1 = 260°C for 10 s; PH-2 = 220°C for 5 s. The results are provided in Fig 4. Although none of the combinations show a >10% deviation from unity, it does appear that some PH combinations give better results. We used a PH-1 of 260°C for 10 s and PH-2 of 160°C for 5 s combination for measurement of all samples.

#### 3.2.4 Rejection of grains

Using the measurement conditions described above, a total of 18,500 grains were measured, but it is well-known that not every grain will result in an accurate estimate of D_e_ (e.g. [46, 47]). Aberrant grains were rejected using the quality-assurance criteria described by Jacobs, Duller [46] and reasons are given in S1 Table. The majority of grains from Riwi (49.2%) were discarded because they were too dim following a laboratory dose (T_N_ signal<3xBG) or the test dose signal was imprecisely known (>20% error on test dose signal). Rejecting such grains does not cause any bias in the results, because they do not contribute to the luminescence signal. Of the 9109 grains remaining, a further ~28% (N = 2606) were rejected (see S1 Table for details), leaving a total of 6776 grains, or an average of ~185 grains per sample, for D_e_ determination.

#### 3.2.5 Accepted grains–decay curve and dose response curve characteristics

Fig 4A and 4C show a selection of decay curves from one Holocene (Riwi-4) and one Pleistocene (Riwi-27) sample, following the test dose to the natural signal (T_N_) of ~11 Gy, and a PH-2 temperature of 160°C for 5 s. They represent the range of sensitivities and shapes and are representative of all samples measured from Riwi. The decay curve shapes are quite reproducible and decay rapidly to instrumental background level; less than 5% of the signal remains after 0.2 s of optical stimulation. Fig 4B and 4D show the corresponding dose response curves for the same grains. The majority of grains have very similar dose response curves up to ~80 Gy (the dose range of interest for samples from Riwi), after which some of the grains continue to grow with increasing dose and others cease to increase at much lower doses.

### 3.3 Equivalent dose (D_e_) determination and results

**Fig 5:**
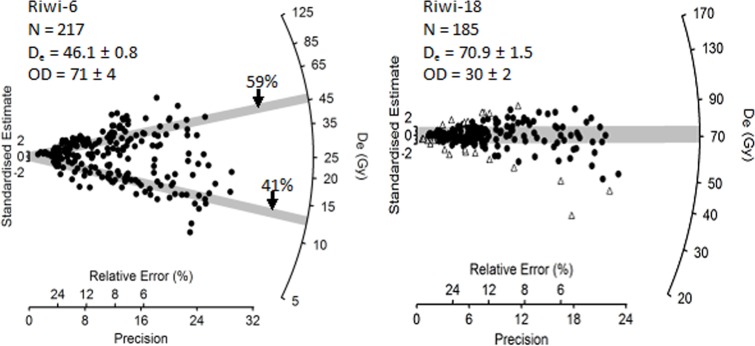
**Radial plot of the D_e_ distribution for the accepted grains in a (A) mixed (Riwi-6) and a (B) scattered (Riwi-18) sample.** The grey bands in (A) are centred on the weighted mean D_e_ determined for each dose population using the Finite Mixture Model. The grey band in (B) is centred on the weighted mean De determined using the central age model after the rejection of outliers (shown as open triangles). Radial plots for all 37 samples are presented in S1 Fig.

Single grain D_e_ values for all samples are displayed as radial plots in S1 Fig. All are spread more widely than can be explained by measurement uncertainties alone, being overdispersed by between 24 ± 2 (Riwi-32) and 118 ± 6% (Riwi-2) (Table 3). We observed two different types of D_e_ distribution—mixed and scattered. A representative example of both types is shown in Fig 5. Only two samples showed mixed D_e_ distributions (Riwi-2 and Riwi-6). Both samples were collected from the upper-most red-brown sands in SU7 that are unconformably overlain by the ashy-grey sands of SU2 (see Fig 3A). We were able to fit the finite mixture model (FMM) of Roberts et al. (2000) to both. This model assumes that grains of discrete dose populations, well bleached prior to deposition, were mixed post-depositionally. The optimum number of fitted components was obtained from the smallest Bayesian Information Criterion (BIC) and maximum log likelihood, using the procedure described in Jacobs, Wintle [48]. The fitting details, D_e_ values for each component and the proportion of grains that make up each component are provided in S2 Table.

**Table 3:** Dose rate data, equivalent doses and OSL ages for sediment samples from Riwi.

Sample	SU	Depth (cm)	Water (%)^#^	Environmental dose rate (Gy/ka)				D_e_ value (Gy)^$^	Number of grains^*^	Overdispersion (%)^&^	Age (ka)^^^	*P-*value
				Beta	Gamma	Cosmic	Total					
Riwi-4	2	13	1.8	1.06 ± 0.05	0.63 ± 0.03	0.05 ± 0.01	1.77 ± 0.06	13.0 ± 0.1	268 (214)	28 ± 1	7.3 ± 0.3 (0.2)	
Riwi-5	2	17	4.3	0.93 ± 0.05	0.65 ± 0.03	0.05 ± 0.01	1.66 ± 0.06	11.7 ± 0.2	234 (191)	31 ± 2	7.1 ± 0.3 (0.2)	
									***Weighted mean =***		***7*.*2 ± 0*.*3***	0.21
Riwi-2	?	20	3.8	1.11 ± 0.05	0.64 ± 0.03	0.05 ± 0.01	1.83 ± 0.06	**49.9 ± 1.7 (53%)**15.3 ± 0.8 (27%)	186	118 ± 6	**27.3 ± 1.4 (1.0)**8.4 ± 0.5 (0.5)	
Riwi-6	?	20	1.5	1.13 ± 0.06	0.63 ± 0.03	0.05 ± 0.01	1.84 ± 0.06	**46.1 ± 0.8 (59%)**12.7 ± 0.3 (41%)	217	71 ± 4	**25.1 ± 1.1 (1.0)**6.9 ± 0.3	
Riwi-1	6	22	7.9	0.92 ± 0.05	0.67 ± 0.03	0.05 ± 0.01	1.67 ± 0.06	57.8 ± 1.0	227 (198)	32 ± 2	34.6 ± 1.5 (0.9)	
Riwi-7	7	23	1.5	1.09 ± 0.05	0.63 ± 0.03	0.05 ± 0.01	1.79 ± 0.06	63.3 ± 1.2	211 (179)	33 ± 2	35.3 ± 1.5 (0.9)	
Riwi-3	7	24	3.2	1.02 ± 0.05	0.64 ± 0.03	0.05 ± 0.01	1.73 ± 0.06	57.4 ± 1.4	179 (146)	64 ± 4	33.1 ± 1.5 (1.1)	
Riwi-8	7	26	3.0	1.06 ± 0.05	0.64 ± 0.03	0.05 ± 0.01	1.77 ± 0.06	67.0 ± 1.1	212 (164)	30 ± 2	37.8 ± 1.6 (0.9)	
									***Weighted mean =***		***35*.*6 ± 1*.*3***	0.13
Riwi-9	8	29	2.4	0.98 ± 0.05	0.63 ± 0.03	0.05 ± 0.01	1.69 ± 0.06	64.1 ± 1.4	185 (159)	34 ± 2	38.0 ± 1.7 (1.1)	
Riwi-10	8	31.5	5.0	0.97 ± 0.05	0.65 ± 0.03	0.05 ± 0.01	1.70 ± 0.06	69.3 ± 1.4	183 (155)	30 ± 2	40.8 ± 1.8 (1.1)	
Riwi-11	8	34	1.8	0.98 ± 0.05	0.63 ± 0.03	0.05 ± 0.01	1.69 ± 0.06	68.0 ± 1.4	207 (185)	27 ± 2	40.2 ± 1.8 (1.1)	
									***Weighted mean =***		***39*.*6 ± 1*.*5***	0.49
Riwi-12	9	36.5	0.6	1.04 ± 0.05	0.65 ± 0.03	0.05 ± 0.01	1.77 ± 0.06	68.9 ± 1.3	203 (167)	29 ± 2	39.0 ± 1.7 (1.0)	
Riwi-13	10	39	2.4	1.09 ± 0.05	0.65 ± 0.03	0.05 ± 0.01	1.82 ± 0.06	72.9 ± 1.9	198 (175)	36 ± 2	40.1 ± 1.9 (1.3)	
Riwi-14	10	42	4.4	1.02 ± 0.05	0.65 ± 0.03	0.04 ± 0.01	1.74 ± 0.06	71.4 ± 1.2	221 (177)	35 ± 2	40.9 ± 1.8 (1.0)	
Riwi-15	10	44.5	2.2	0.99 ± 0.05	0.65 ± 0.03	0.04 ± 0.01	1.72 ± 0.06	68.6 ± 1.6	186 (158)	34 ± 2	40.0 ± 1.8 (1.2)	
Riwi-16	10	47	1.3	1.05 ± 0.05	0.65 ± 0.03	0.04 ± 0.01	1.78 ± 0.06	68.2 ± 1.3	184 (149)	29 ± 2	38.4 ± 1.7 (1.0)	
Riwi-17	10	50	1.7	1.04 ± 0.05	0.63 ± 0.03	0.04 ± 0.01	1.75 ± 0.06	77.4 ± 1.8	173 (144)	33 ± 2	44.3 ± 2.0 (1.3)	
Riwi-18	10	52	1.7	0.99 ± 0.05	0.63 ± 0.03	0.04 ± 0.01	1.69 ± 0.06	70.9 ± 1.5	185 (161)	30 ± 2	41.9 ± 1.9 (1.2)	
Riwi-19	10	55	1.7	0.99 ± 0.05	0.63 ± 0.03	0.04 ± 0.01	1.69 ± 0.06	72.3 ± 1.5	214 (189)	30 ± 2	42.9 ± 1.9 (1.2)	
Riwi-20	10	60	2.0	0.98 ± 0.05	0.63 ± 0.03	0.04 ± 0.01	1.68 ± 0.06	69.2 ± 1.4	203 (182)	31 ± 2	41.1 ± 1.8 (1.1)	
									***Weighted mean =***		***41*.*1 ± 1*.*6***	0.43
Riwi-21	11	63	1.9	1.00 ± 0.05	0.63 ± 0.03	0.04 ± 0.01	1.70 ± 0.06	73.0 ± 2.2	174 (137)	35 ± 2	43.0 ± 2.1 (1.3)	
Riwi-22	11	66	4.9	0.97 ± 0.05	0.65 ± 0.03	0.04 ± 0.01	1.69 ± 0.06	76.2 ± 1.9	178 (164)	35 ± 2	45.0 ± 2.1 (1.4)	
Riwi-23	11	71	5.5	0.98 ± 0.05	0.65 ± 0.03	0.04 ± 0.01	1.70 ± 0.06	73.4 ± 1.8	208 (180)	32 ± 2	43.1 ± 2.0 (1.2)	
Riwi-24	11	74	4.0	0.97 ± 0.05	0.64 ± 0.03	0.04 ± 0.01	1.68 ± 0.06	67.7 ± 1.2	258 (221)	34 ± 2	40.2 ± 1.7 (1.0)	
Riwi-25	11	77	3.4	0.95 ± 0.05	0.64 ± 0.03	0.04 ± 0.01	1.66 ± 0.06	72.9 ± 1.2	217 (183)	28 ± 2	44.0 ± 1.9 (1.1)	
									***Weighted mean =***		***42*.*8 ± 1*.*6***	0.58
Riwi-26	12	80	1.7	0.87 ± 0.04	0.63 ± 0.03	0.04 ± 0.01	1.56 ± 0.05	66.2 ± 1.3	135 (117)	29 ± 2	42.4 ± 1.9 (1.2)	
Riwi-27	12	83	3.0	0.90 ± 0.05	0.64 ± 0.03	0.04 ± 0.01	1.61 ± 0.05	68.2 ± 1.5	142 (125)	27 ± 2	42.4 ± 1.9 (1.2)	
Riwi-28	12	85.5	4.5	0.94 ± 0.05	0.65 ± 0.03	0.04 ± 0.01	1.65 ± 0.06	71.6 ± 1.8	141 (126)	35 ± 2	43.3 ± 2.0 (1.4)	
Riwi-29	12	88	4.8	0.93 ± 0.05	0.65 ± 0.03	0.04 ± 0.01	1.65 ± 0.06	76.8 ± 2.1	130 (113)	37 ± 3	46.5 ± 2.2 (1.6)	
Riwi-30	12	91	4.2	0.92 ± 0.05	0.65 ± 0.03	0.04 ± 0.01	1.63 ± 0.06	74.0 ± 1.5	158 (129)	29 ± 2	45.3 ± 2.0 (1.2)	
Riwi-31	12	93	0.3	0.89 ± 0.05	0.62 ± 0.03	0.04 ± 0.01	1.57 ± 0.05	76.6 ± 1.6	160 (135)	32 ± 2	48.8 ± 2.2 (1.4)	
Riwi-32	12	96	1.9	0.93 ± 0.05	0.63 ± 0.03	0.04 ± 0.01	1.62 ± 0.06	73.3 ± 1.5	156 (135)	24 ± 2	45.2 ± 2.0 (1.2)	
Riwi-33	12	99	1.9	0.93 ± 0.05	0.63 ± 0.03	0.03 ± 0.01	1.62 ± 0.06	78.5 ± 1.9	136 (132)	34 ± 3	48.4 ± 2.2 (1.5)	
Riwi-34	12	102	2.9	0.90 ± 0.05	0.64 ± 0.03	0.03 ± 0.01	1.60 ± 0.05	69.4 ± 1.9	140 (116)	42 ± 3	43.5 ± 2.1 (1.5)	
Riwi-35	12	105	2.4	0.94 ± 0.05	0.63 ± 0.03	0.03 ± 0.01	1.63 ± 0.06	75.8 ± 2.3	117 (110)	29 ± 2	46.4 ± 2.3 (1.7)	
Riwi-36	12	108	1.9	0.95 ± 0.05	0.63 ± 0.03	0.03 ± 0.01	1.65 ± 0.06	84.6 ± 1.8	130 (105)	29 ± 2	51.3 ± 2.3 (1.4)	
Riwi-37	12	110	3.4	0.94 ± 0.05	0.64 ± 0.03	0.03 ± 0.01	1.64 ± 0.06	80.2 ± 2.3	128 (114)	38 ± 3	48.9 ± 2.4 (1.7)	
									***Weighted mean =***		***46*.*7 ± 1*.*7***	0.075

^#^Represent the current measured water content of the sediment. A relative uncertainty of ±40% (at 1σ) was assigned to each estimate of water content. A water content of 5 ± 2% was used for all samples in calculations of dose rate.

^$^D_e_ values for all samples were obtained using the central age model (CAM), except for Riwi-2 and Riwi-6 where the finite mixture model (FMM) was used. The D_e_ values are those for the two components that represent the highest number of grains and the proportions are indicated in brackets.

*Numbers in brackets represent the number of grains included in the CAM D_e_ value after identification and rejection of outlier (nMAD) grains.

^&^The OD values are for the D_e_ distributions that include all D_e_ values, including those identified as outliers. The OD values for the samples where the outliers were rejected are provided in S1 Table.

^Numbers in brackets represent the random-only error on the age.

The remaining samples display scattered D_e_ distributions, probably due to bioturbation and micro-scale differences in the beta dose rate received by individual grains. To obtain D_e_ values we calculated the weighted mean D_e_ using the central age model (CAM) [49] which assumes the D_e_ values for all grains are centered on some average value of D_e_ (similar to the median) and the estimated standard error takes account of any overdispersion (i.e., the greater the overdispersion, the larger the error).

As some grains might have been reworked after deposition, their D_e_ values should be removed before calculating the weighted mean D_e_ values. The median absolute deviation is widely used to screen data for outliers [50, 51]. It is calculated as the median of all absolute distances from the sample median and attaches equal importance to positive and negative deviations from the sample median. After converting the D_e_ values (in Gy) to natural logarithms [52], we calculated the normalised median absolute deviations (nMADs) using 1.4826 as the appropriate correction factor for a normal distribution, and rejected log D_e_ values with nMADs greater than 1.5 [53]. The outlier D_e_ values are shown as open triangles in each of the radial plots in Fig 5 and S1 Fig, and make up between 3% (Riwi-33) and 23% (Riwi-8) of the total number of D_e_ values in each sample. Ratios of the outlier rejected CAM D_e_ value to the CAM D_e_ value with all values included are provided in S3 Table and range between 0.97 (Riwi-3) and 1.19 (Riwi-5), with an average for all samples of 3 ± 4%. A consequence of the outlier detection and rejection is that the OD value for each sample is reduced (S3 Table), and as a result the weighted mean D_e_ values are more precise; the standard error on the weighted mean D_e_ values for each sample decreases from an average of 2.8 to 2.1%.

### 3.4 Dose rate determination and results

The total dose rate consists of contributions from beta, gamma and cosmic radiation external to the grains, plus a small alpha dose rate due to the radioactive decay of U and Th inclusions inside sand-sized grains of quartz. We have assumed that the measured radionuclide activities and dose rates have prevailed throughout the period of sample burial. All dose rates were corrected for long-term water contents assuming a moisture content of 5 ± 2% for all samples. This is consistent with the range of current field values that ranged between 0.3% (Riwi-31) and 7.9% (Riwi-1) (Table 3), with a median and standard deviation of 2.5 ± 1.5%. In general, the calculated total dose rate will decrease, and the calculated OSL age will increase, by ~1% for each 1% increase in water content.

An internal alpha dose rate of 0.032 ± 0.01 Gy/ka has been assumed for all samples. The beta dose rates were estimated by low-level beta counting of dried, homogenised and powdered sediment samples using a GM-25-5 multi-counter system [54] following the procedures described and tested in Jacobs and Roberts [55]. Allowance was made for the effect of sample moisture content [56], grain size [57] and hydrofluoric acid etching [58] on beta-dose attenuation. The beta dose rates are provided in Table 3 and range between 0.87 ± 0.04 (Riwi-26) and 1.13 ± 0.06 (Riwi-6).

Gamma dose rates were measured by *in situ* gamma spectrometry. Counts were collected for 30 min with a 1-inch NaI(Tl) crystal. The detectors were calibrated using the concrete blocks at Oxford University [59] and the gamma dose rates were determined using the ‘threshold’ technique [60]. We did not measure the gamma dose rate at each sampling location; the deposit is only ~ 1 m deep and most samples were collected as a continuous column. Instead, we obtained measurements at 3 depths down this column (25, 60 and 90 cm below the surface) and an additional measurement in the sample hole left after collecting sample Riwi-3. The four results were consistent (0.64 ± 0.03, 0.64 ± 0.03, 0.67 ± 0.03 and 0.65 ± 0.03), so we used the average (0.65 ± 0.03) as the gamma dose rate estimate for all samples. Small variations (0.62–0.65 Gy/ka) occurred because we corrected each of the values for the current field water content at each sample location.

The cosmic-ray contribution was adjusted for the average site altitude (~115 m), geomagnetic latitude (-29.4°), density and thickness of rock and sediment overburden [61]. They range from 0.05 ± 0.01 to 0.03 ± 0.01 (Table 3) between the top and bottom of the excavated square.

The dose rate results for all samples from Riwi are provided in Table 3. The total dose rates for all the samples show only a modest amount of variation, ranging between 1.56 ± 0.05 (Riwi-26) and 1.84 ± 0.06 Gy/ka (Riwi-6).

### 3.5 OSL age estimates

The final ages for all samples are listed in Table 3, together with the supporting D_e_ and dose rate estimates. Uncertainties on the ages are given at 1σ (standard error on the mean) and were derived by combining, in quadrature, all known and estimated sources of random and systematic error. We were able to obtain reliable ages for all 37 samples collected from Riwi. The ages range from ~7 ka for samples from SU2 to ~50 ka for samples near the base of the deposit in SU12.

## 4. ^14^C and OSL Age Comparisons in a Bayesian Framework

### 4.1 Construction of Bayesian models

The unusually large number of ^14^C and OSL age estimates obtained at such high resolution provides an opportunity to make meaningful comparisons between the techniques. Bayesian models were built using the OxCal v.4.2 platform (Ramsey 2009a) to reduce the uncertainty of age estimates for particular events and to allow assessment of the correspondence between ages obtained using the same and/or different dating methods. In the text, all modelled age ranges are given at 95.4% probability, unless otherwise stated.

Two separate models were constructed using ages obtained by the two dating methods. All ^14^C ages were calibrated against SHCal13 [30] in OxCal v.4.2 [31]. Where two radiocarbon dates exist on the same sample, the result of the weighted average was calibrated using the function *R_Combine*. Each OSL age was input as a *C_Date* in calendar years before 1950 with an associated 1σ error. OSL ages do not have fully independent uncertainties; many of the errors are shared among all the OSL ages (i.e., systematic errors). When ages with common systematic errors are combined, only the random errors (given in brackets in Table 3) should be included, and so only the random errors were included in the Bayesian model [62].

For the ^14^C model, each stratigraphic unit (SU) was modelled as a *Phase* in which the measured ages are assumed to be unordered and uniformly distributed. In contrast to the radiocarbon dates undertaken on charcoal across the excavated area, the majority of OSL samples were collected in a continuous column meaning that depth is likely to relate to age. Therefore, each stratigraphic unit was modelled as a *Sequence*, assuming that ages from the bottom of the SU are older than those at the top. A *Boundary* was placed at the start and end of each *Phase* or *Sequence*. The modelled probability distributions of these *Boundaries* provide an estimate for the start and end of a SU. These components were then arranged into a *Sequence*, assuming that the lowest context (SU12) is older than those stratigraphically higher. Holocene and Pleistocene models were run separately so that the models converged faster.

For both models, a *General t-type Outlier Model* [63] was used to assess the likelihood of each OSL or radiocarbon measurement being consistent. Each date was assigned a prior outlier probability of 5%. During the modeling process, the posterior outlier probability is calculated and the date down-weighted accordingly. For example, if the posterior probability is found to be 5%, the date is included in 95% of the model iterations, but if it is found to be 50% it is included in only 50% of model iterations.

### 4.2 The ^14^C model

**Fig 6:**
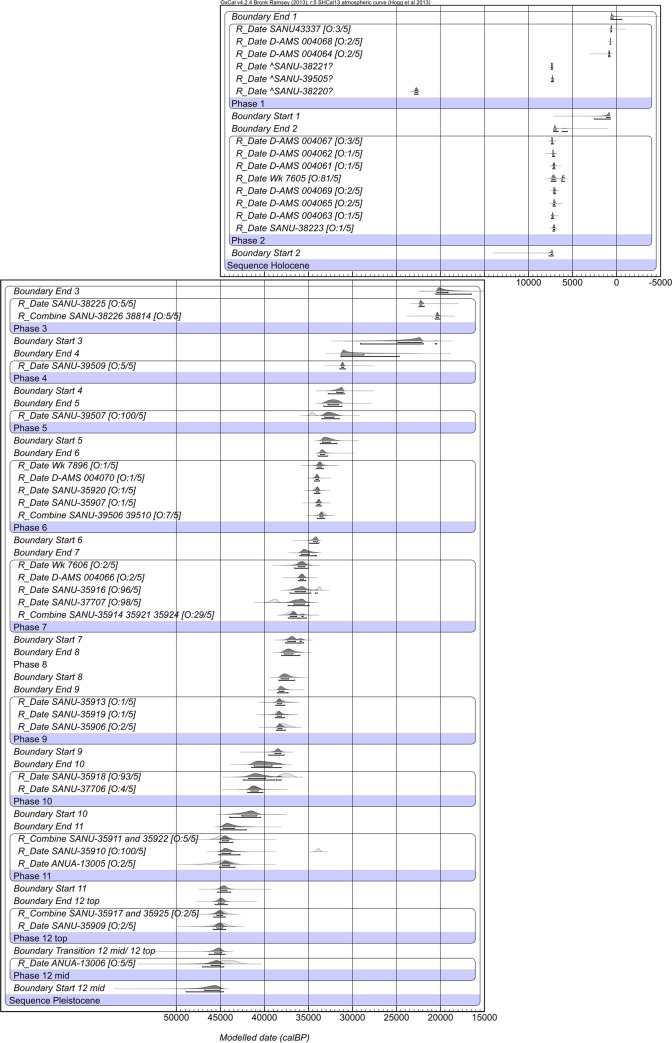
Bayesian model of the radiocarbon dates from Riwi. Dates have been calibrated against SHCal13 [30] and modelled in OxCal v.4.2 [31]. Pale probability distributions represent the calibrated, unmodelled date, whilst dark grey distributions represent the modelled date. The two brackets beneath the distributions represent the 68.2% and 95.4% probability ranges. Prior and posterior outlier probabilities are given in brackets following the sample name in the form [O: posterior/prior]. A ‘?’ implies the date was not included in the model.

The ^14^C model is shown in Fig 6 and S4 Table. 36 dated charcoal samples were included in the model, including both the newly obtained (Table 2) and published ages (Table 1). Wk-7607 is not finite and could not be included in the model. We also omitted one very obvious outlier from the relatively bioturbated stratigraphic unit SU1 (SANU-38220, square 4, which decreased the convergence of previous models), and two samples found at the interface between the two Holocene SUs (SANU-38221 from square 4 and SANU-39505, square 3). Although not included in the model, these are plotted in Fig 6.

A further six samples were identified as outliers by the model (at more than 80%). Interestingly, all were detrital charcoal pieces found outside hearth features. No charcoal sample collected from a hearth feature was identified as an outlier. It is likely that these outliers suggest minor movement of charcoal through the sediment. Although the potential for movement of charcoal through sediments is frequently commented upon, it is rarely observed in contexts where stratigraphic units and features can be identified. Riwi provides a clear example of this phenomenon, stressing the importance of sampling from discrete charcoal lenses or hearths.

### 4.3 The OSL model

**Fig 7:**
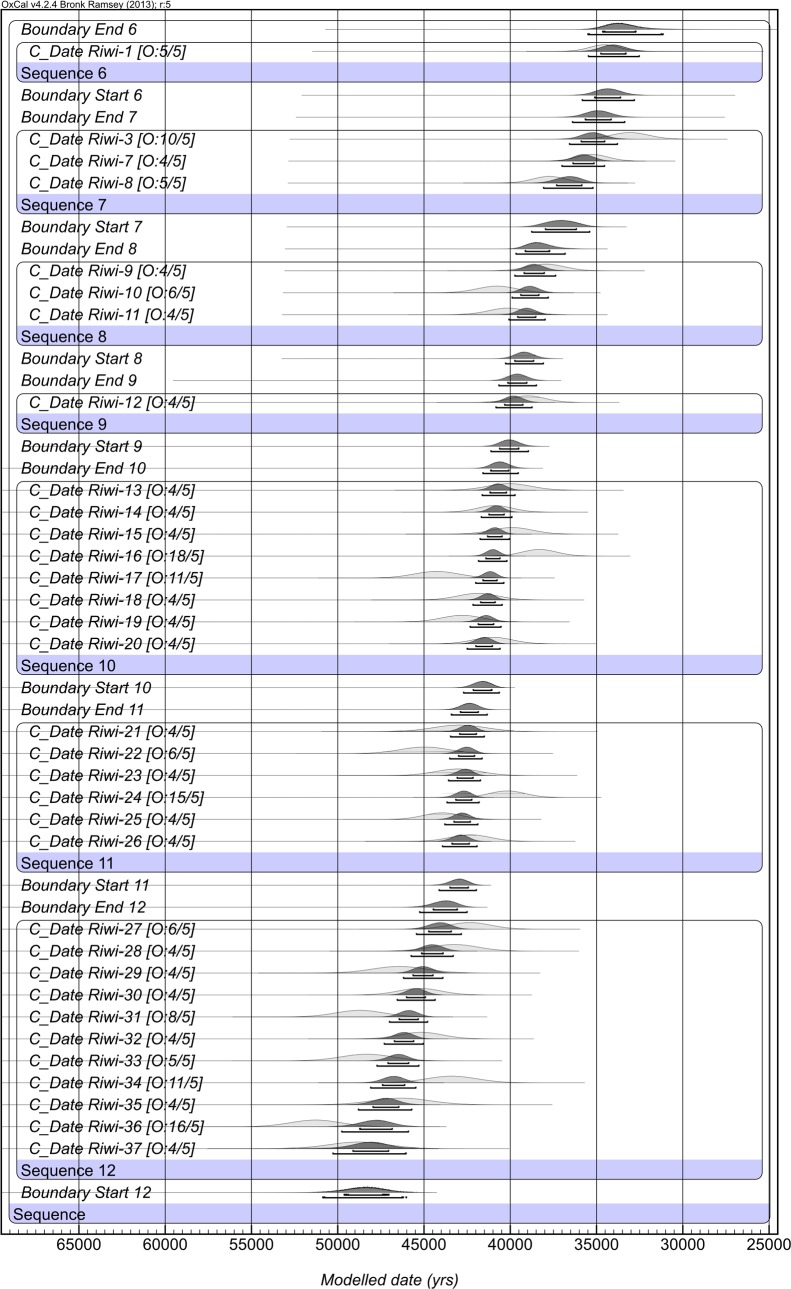
Bayesian model of the OSL dates from Riwi. Only random errors are included. For explanation of figure interpretation, see Fig 6.

The OSL Bayesian modelled sequence is shown in Fig 7 and data provided in S5 Table. 33 OSL age estimates listed in Table 3 were included in the Pleistocene model. A Holocene model was not run as only two dates are available. Riwi-2 and Riwi-6 were omitted from the model as they contained grains from more than one age population (S1 Fig).

The OSL ages are relatively consistent with each other and the stringent stratigraphic priors applied. The model identified five ages as having more than 10% posterior probability of being an outlier. However, none of the samples have an outlier probability of more than 18%, samples are found to be both too old and too young, and the samples were spread throughout the stratigraphy, suggesting that inaccuracies are random and of a small magnitude.

### 4.4 Comparing the models

The *Difference* function was used to examine whether the ^14^C and OSL age estimates produced consistent age models. This function subtracts one probability distribution function from another. If zero is included in the 95.4% probability range, the probability distribution functions are regarded as indistinguishable at 95.4%. It needs to be kept in mind that for OSL ages to be compared with other independent ages, the systematic errors omitted from individual age uncertainties to run the model, must now be added to the errors on the modelled start and end dates obtained for each SU.

**Fig 8:**
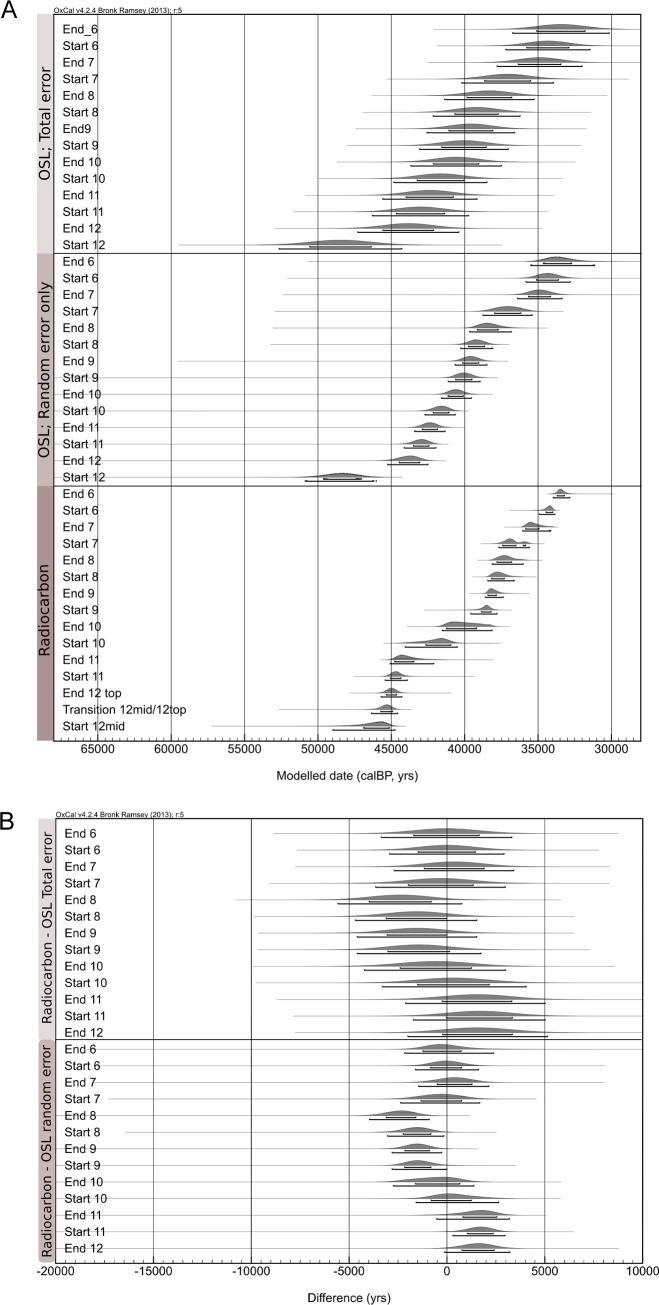
A comparison of Boundaries generated by the radiocarbon and OSL Bayesian models. (A) illustrates the Boundaries of the radiocarbon and OSL models, alongside the estimated Boundaries if the systematic error is included in the OSL model, and (B) the *Difference* between the Boundaries calculated in the radiocarbon model and the OSL model with and without the systematic error estimation.

To estimate the total OSL uncertainty, the mean and standard deviation of each *Boundary* within the OSL model was calculated in OxCal. This is reasonable as the probability distributions of the *Boundaries* within the OSL model approximate a normal distribution (Fig 7). The average relative systematic error (3.5% of the age estimate), representative for all stratigraphic units, was then combined with the modeled standard deviation in quadrature providing an estimate of the total error. The effect of this is illustrated in Fig 8A.

The results of the *Difference* function are given in Table 4 and Fig 8B. When the systematic uncertainty of the OSL dates is added to the OSL model *Boundaries*, the two models are consistent as all *Boundaries* overlap at 95.4% probability. Uncertainties in the accuracy of the radiocarbon calibration curve around 40 ka [64] are not significant within the typical precision of the OSL chronology (5000 yrs at 95.4% probability), and it is therefore likely that the agreement suggests both techniques produced accurate dates. As expected, when the systematic uncertainty is not considered the agreement is not as strong; the *Difference* between four *Boundaries* does not include zero. However, the offsets between the models are not systematic. At times the radiocarbon model is older than the OSL (Start 11), and at others the radiocarbon is younger (End 9 –End 8). It should also be noted that no radiocarbon dates were obtained from SU8 where the strongest deviation occurs.

**Table 4:** Difference between Boundaries from the models of the radiocarbon and OSL ages calculated in OxCal. If zero is included in the probability range the two probability distributions are considered indistinguishable.

Name	Difference			
	68.2% probability range		95.4% probability range	
	from	to	from	to
Radiocarbon—OSL total error				
Difference End 6	-1700	1650	-3370	3310
Difference Start 6	-1480	1450	-2930	2920
Difference End 7	-1160	1900	-2700	3410
Difference Start 7	-1980	1350	-3650	2990
Difference End 8	-3980	-800	-5580	760
Difference Start 8	-3120	-10	-4680	1520
Difference End 9	-3060	0	-4590	1520
Difference Start 9	-3030	130	-4590	1730
Difference End 10	-2400	1250	-4230	2990
Difference Start 10	-1510	2160	-3300	4050
Difference End 11	-250	3310	-2120	5030
Difference Start 11	-30	3340	-1720	5020
Difference End 12	-230	3340	-2000	5140
Radiocarbon—OSL random error				
Difference End 6	-1240	730	-2160	2380
Difference Start 6	-840	750	-1610	1620
Difference End 7	-500	1280	-1460	2140
Difference Start 7	-1340	760	-2370	1680
Difference End 8	-3110	-1590	-3960	-910
Difference Start 8	-2240	-820	-3040	-150
Difference End 9	-2150	-890	-2810	-270
Difference Start 9	-2170	-820	-2810	0
Difference End 10	-1610	660	-2740	1380
Difference Start 10	-810	1250	-1590	2650
Difference End 11	810	2550	-530	3190
Difference Start 11	1050	2360	310	2960
Difference End 12	770	2410	-130	3220

### 4.5 The chronology of Riwi

Given the agreement between the ^14^C and OSL chronologies, the chronology of the deposits at Riwi can be discussed considering the different types of information provided by each technique. Layer 12 started to form after 50.9–46.0 ka (Boundary Start 12, OSL model). The first hearth feature was found at or on the top of layer 12. Radiocarbon dates on charcoal from this feature provide a direct date of human activity. The model suggests that the hearth started to form at 46.4–44.6 cal kBP (Transition 12 mid/ 12 top, Radiocarbon model), giving the earliest secure date of human occupation in the cave. Some lithic artefacts were found at a greater depth, but it is not possible to test whether they are slightly earlier than the hearth feature or if they were associated with the hearth, e.g. were lying on an uneven surface or pushed to a greater depth through trampling. However, given the rapid sedimentation at the top of layer 12 as indicated by the OSL dates, the maximum possible age of these is similar to the hearth feature.

The age estimate for the start of sediment deposition at Riwi (Boundary Start 12, OSL model) and the first *in situ* occupation (Transition 12 mid/ 12 top, Radiocarbon model) are indistinguishable at 95.4% probability (-7340–1230 yrs), and sediment accumulation was instantaneous within the precision of the models. However, it is likely that there is between 920–5210 yrs (68.2% probability) of archaeologically sterile sediment present prior to the first arrival of people at Riwi.

Sedimentation was rapid between SU12 top–SU4, with around 0.5 m of sediment deposited in 16.8–13.8 cal kyr (68.2% probability; Radiocarbon Transition 12 mid/ 12 top and Radiocarbon End 4). During this period, occupation appears relatively continuous until around 30 cal kBP, with hearth features (Fig 3A and 3B), lithic artefacts and bone present throughout the deposit. After this relatively continuous sedimentation, a series of chronological hiatus’ are present, either due to erosion events, or more likely, a slowing of sedimentation due to the filling of the cave. The hiatus’ occur between c.30–21 cal kBP, between c.21–7 cal kBP and between c.7–1 cal kBP (Fig 8), and are interspersed with short, discrete pulses in sedimentation.

Without a sedimentary archive, it is not possible to say whether occupation continued throughout these hiatus’ at Riwi. Given the earlier hypotheses suggesting that the arid zone was not occupied during the LGM [15, 26, 27], the pulse in sedimentation dating to the LGM (SU3) is of particular interest. SU3 is found only in the SE corner of square 3 (Fig 8) where it’s preservation may be related to the presence of mudnest building by mud dauber wasps, which created a hard capping. In the remaining excavated area, the older Pleistocene sediments (SU7, 6 and 4) are directly in contact with Holocene deposits (SU1 and 2). The preservation of a single hearth feature dating to the LGM provides a glimpse that people were present during at least one episode within the major discontinuity, and highlights the old adage that an absence of evidence is not evidence of absence. In this case, without archaeologically sterile sediments dated to the LGM, it is not possible to say that occupation did not occur.

## 5. Conclusion

At Riwi, high resolution sampling of sand for OSL and charcoal for radiocarbon dating in the Late Pleistocene (c.30 – c.50 ka) have enabled Bayesian models for each method to be built and compared. Agreement between the techniques is excellent, giving confidence that both are likely to be accurate and have appropriate estimates of precision. Riwi contains ideal samples for both OSL and radiocarbon. Sediments are rich in clean quartz sand, whilst charcoal, though poorly preserved, is abundant in hearth features and is not contaminated with young carbon. Therefore, whilst we have demonstrated agreement between the methods in the best-case scenario, future work comparing the methods in more challenging deposits must continue.

Careful consideration of precision is required when addressing specific archaeological and palaeoenvironmental questions. Discussions over the earliest colonization of Australia rarely consider precision of the chronologies, or what precision is required to answer the question being asked. For example, Allen and O’Connell [65] use the term ‘central tendency(ies)’ nine times in a review of the age of the earliest colonization, without considering the uncertainties of several thousand years in each case. At Riwi, we have dated the start of the first occupation to 46.4–44.6 cal kBP, in accordance with the prevailing view of colonization at or prior to 47–48 ka. The importance of the age estimate is in its precision; just 1800 cal yrs at 95.4% probability. This is currently the most precisely dated ‘early’ occupation, and able to test the chronology of the arrival of humans in Australia. As further sequences in Australia and SE Asia are dated using similar techniques, high resolution sampling and statistical analyses, it will be possible to compare site chronologies to estimate when people arrived, rates of spread and connections to palaeoenvironmental changes, and enter these into larger models of early modern human dispersals.

## Supporting Information

S1 Fig Radial plots of single-grain D_e_ values for each of the samples measured from Riwi, presented in stratigraphic order. The shaded bands are centred on the weighted mean D_e_ values determined using CAM or FMM. The De values identified as outliers are shown as open triangles. The weighted mean D_e_ used in final age calculation and the overdispersion values for each sample are also provided.
(DOCX)

S1 Table Number of single-grains measured, rejected and accepted, together with the reasons for their rejection. (DOCX)

S2 Table Fitting details, D_e_ values for each component and the proportion of grains in each component for the two samples with mixed D_e_ distributions for which the finite mixture model was used. (DOCX)

S3 Table **Weighted mean De values and overdispersion values for each sample following two different scenarios**: (a) including all De values in each samples, or (b) rejecting outlier values identified as log De values with normalised median absolute deviations (nMADs) greater than 1.5. Also provided in the final column is the ratio of the latter over the former.
(DOCX)

S4 Table Bayesian model of radiocarbon dates. Where dates have not been included in the model, no Modelled probability age range is given.
(DOCX)

S5 Table Bayesian model of OSL dates, including random error only. Dates are in years before 1950 to enable comparison with radiocarbon age estimates.
(DOCX)
