# Supplementary material for: Towards an Accurate and Precise Chronology for the Colonization of Australia: The Example of Riwi, Kimberley, Western Australia
Source: PLoS One. 2016 Sep 21;11(9):e0160123. doi: 10.1371/journal.pone.0160123 (PMC5031455; doi:10.1371/journal.pone.0160123)
Supplement: S1 Fig — The shaded bands are centred on the weighted mean De values determined using CAM or FMM. The De values identified as outliers are shown as open triangles. The weighted mean De used in final age calculation and the overdispersion values for each sample are also provided. (DOCX) [file pone.0160123.s001.docx]

| **Towards an accurate and precise chronology for the colonization of Australia: The example of Riwi, Kimberly, Western Australia**  Wood, R.^1*^, Jacobs, Z.^2^, Balme, J.^3^, O’Connor, S.^4^, Vannieuwenhuyse, D.^3^, Whitau, R.^4^  *^1^Research School of Earth Sciences, Australian National University, Canberra, 2601, Australia*  *^2^Centre for Archaeological Science, School of Earth and Environmental Sciences, University of Wollongong, 2522, Australia*  *^3^School of Social Sciences, University of Western Australia, Crawley, 6009, Australia*  *^4^Department of Archaeology and Natural History, Research School of Pacific and Asian Studies, Australian National University, Canberra, 2601, Australia* |
| --- |

S1 Fig: Radial plots of single-grain D_e_ values for each of the samples measured from Riwi, presented in stratigraphic order. The shaded bands are centred on the weighted mean D_e_ values determined using CAM or FMM. The De values identified as outliers are shown as open triangles. The weighted mean D_e_ used in final age calculation and the overdispersion values for each sample are also provided.


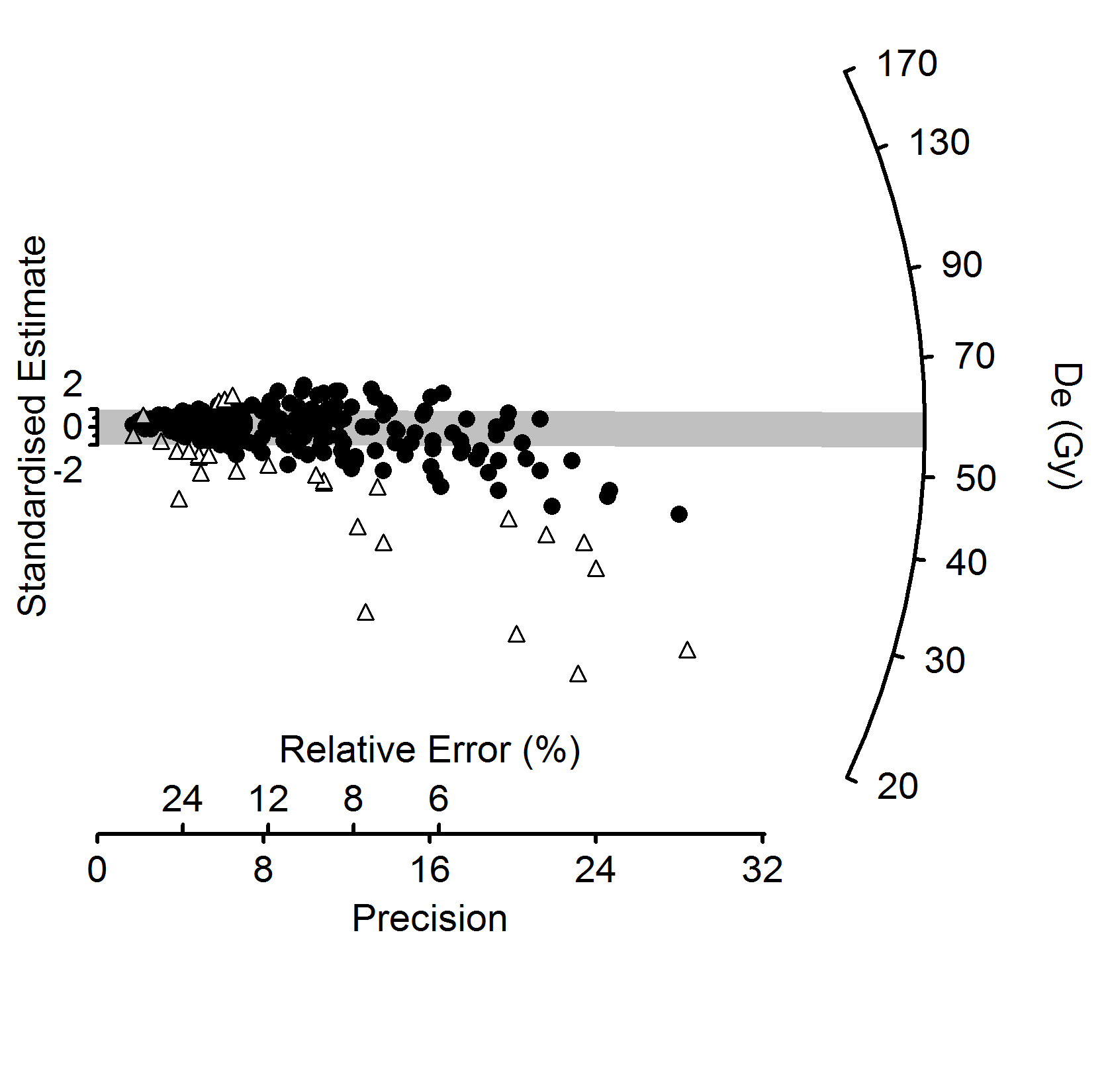

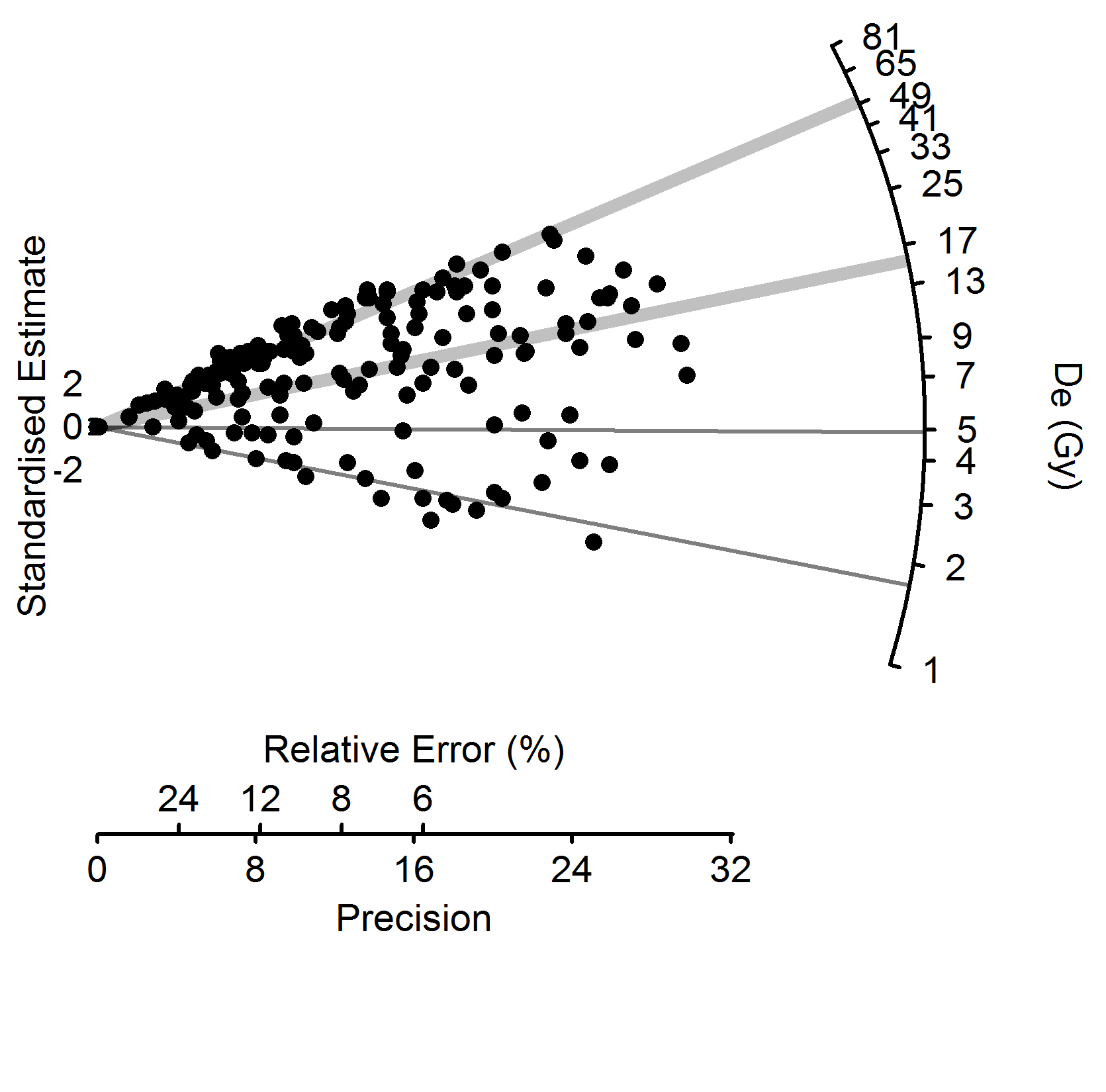


Riwi-1

N = 227

D_e_ = 57.8 ± 1.0

OD = 32 ± 2

Riwi-2

N = 185

D_e_ = 49.9 ± 1.7

OD = 118 ± 6

53%

27%

9%

11%

Riwi-3

N = 179

D_e_ = 57.4 ± 1.4

OD = 64 ± 4

Riwi-4

N = 268

D_e_ = 13.0 ± 0.1

OD = 28 ± 1


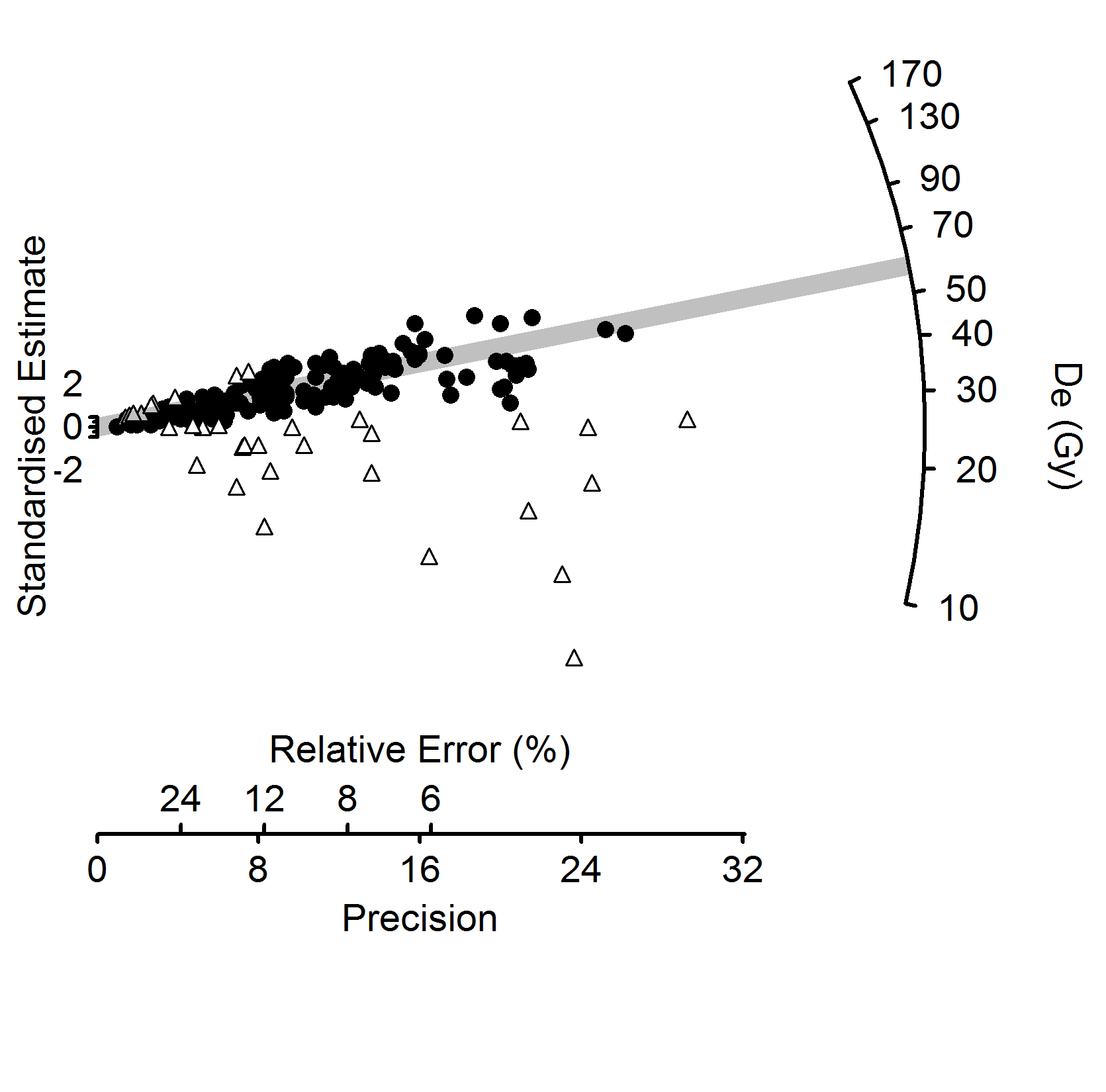

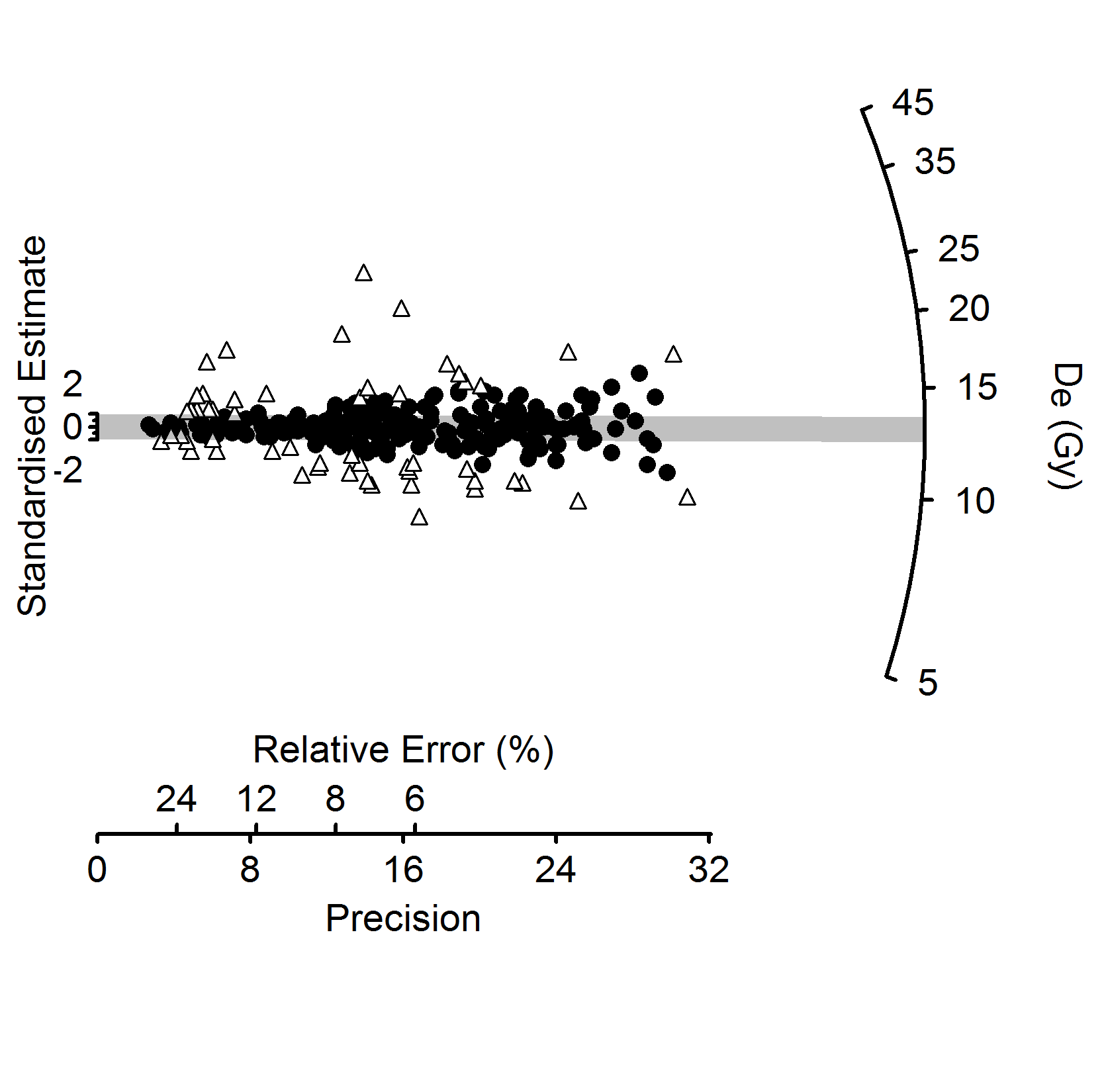


Riwi-5

N = 234

D_e_ = 11.7 ± 0.2

OD = 31 ± 2

Riwi-6

N = 217

D_e_ = 46.1 ± 0.8

OD = 71 ± 4


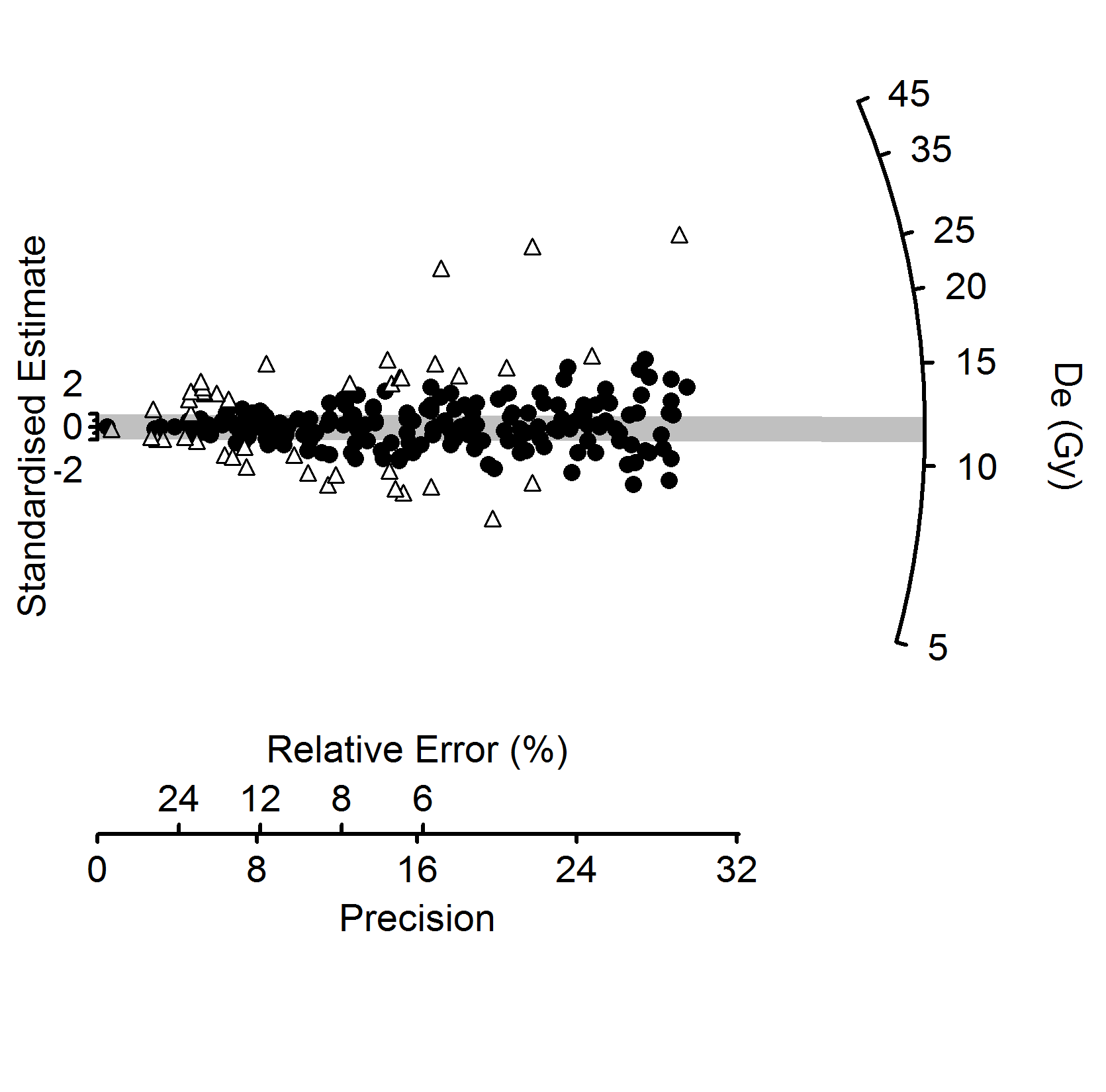

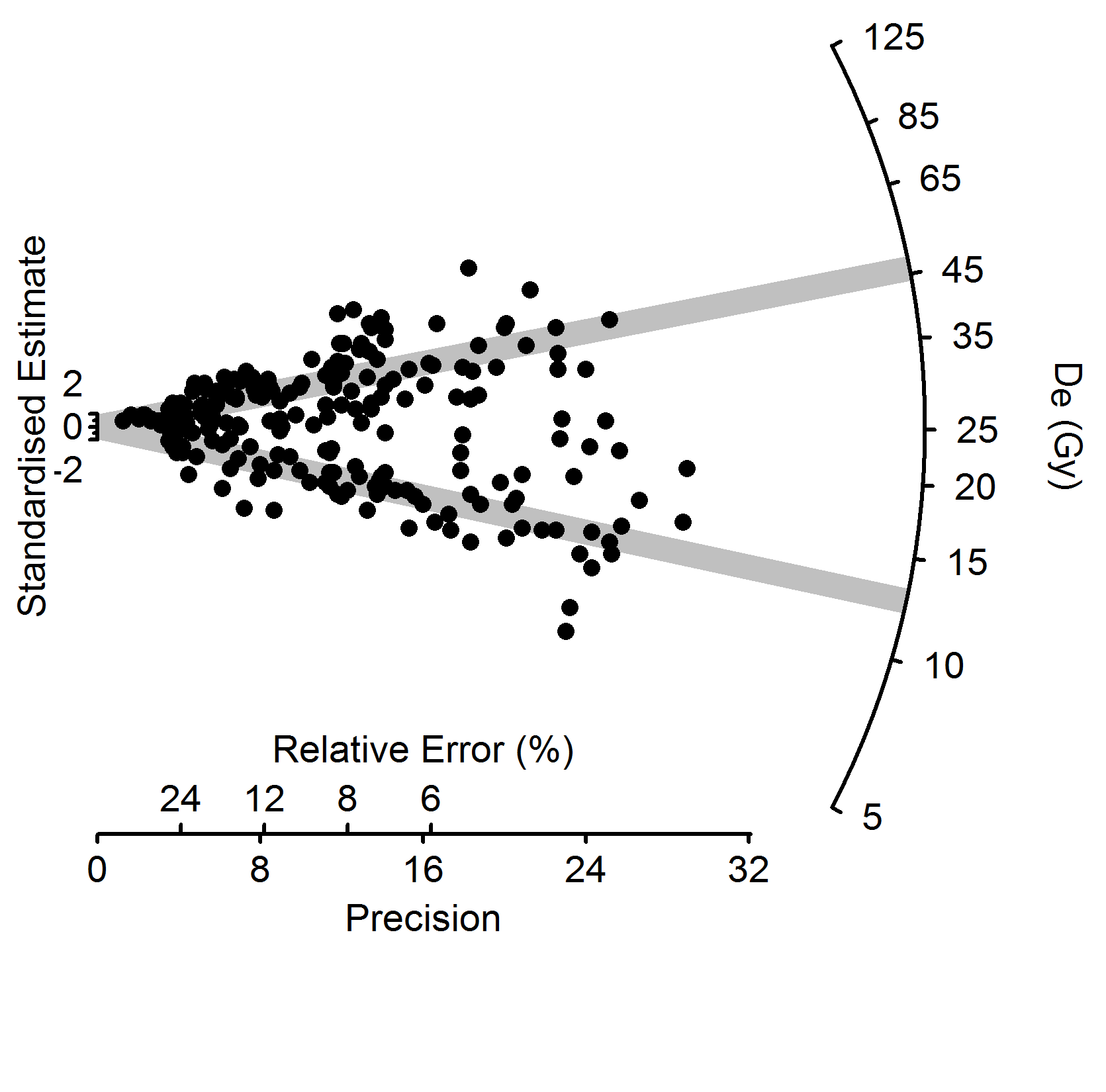


59%

41%


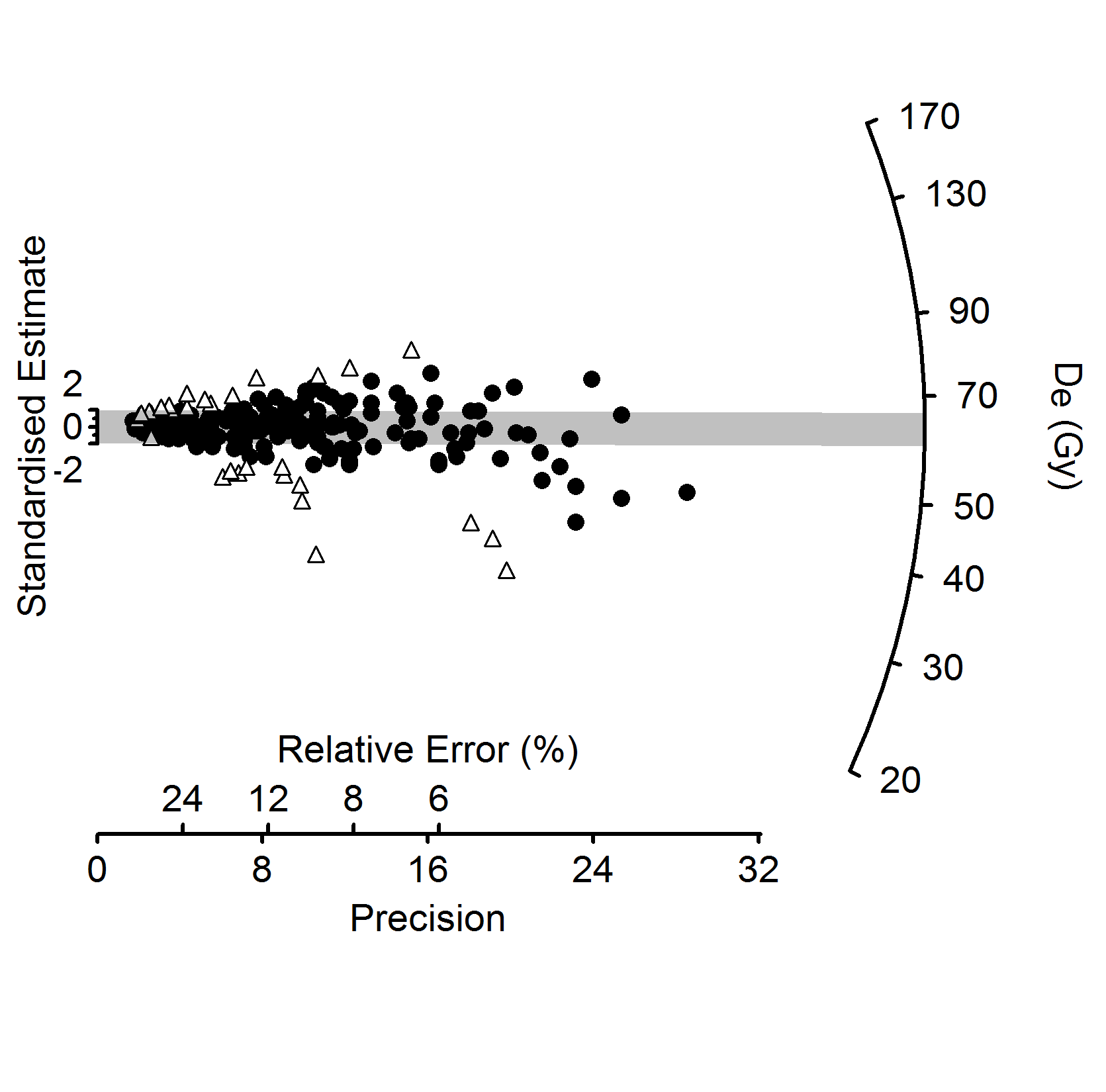

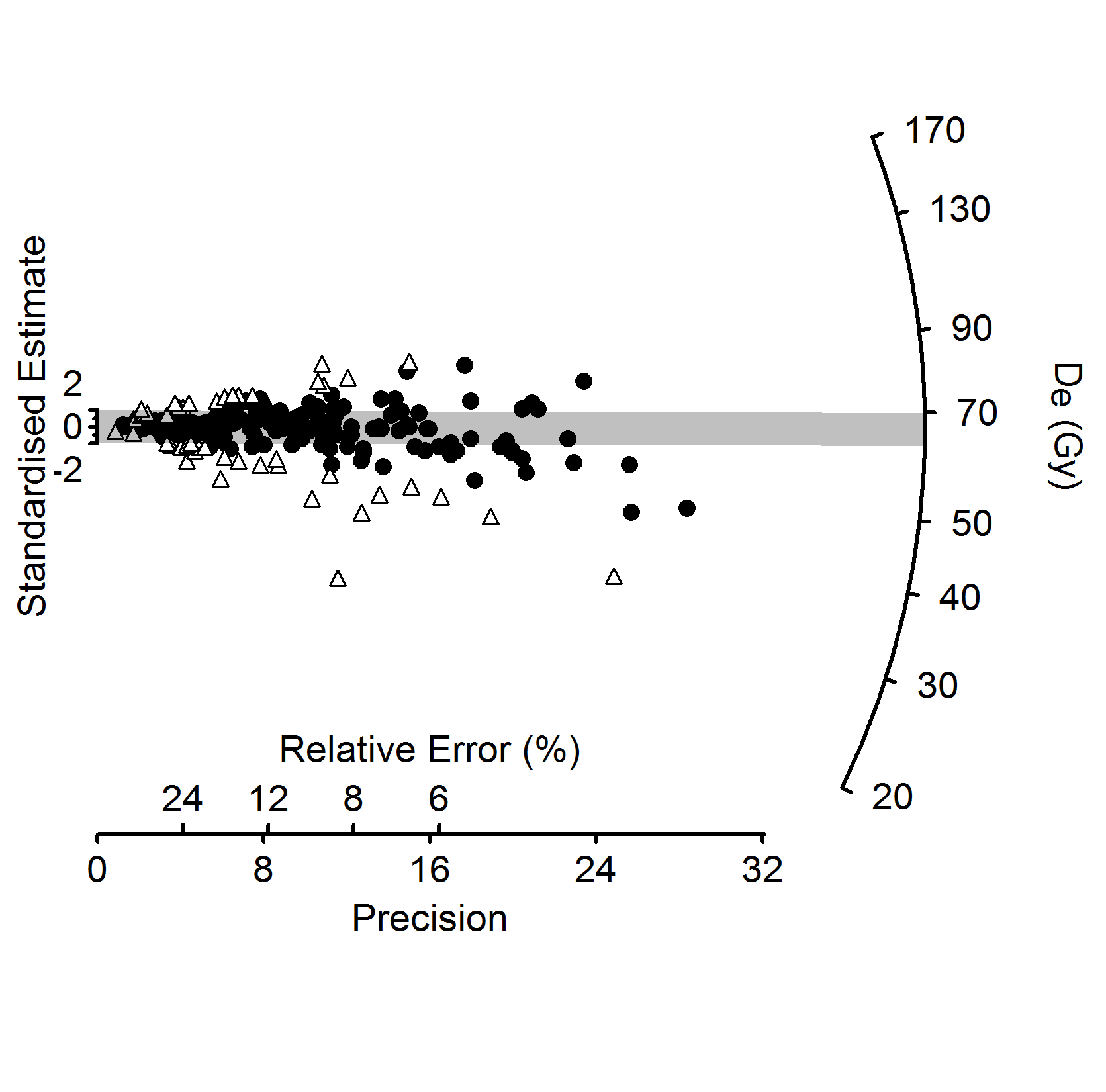


Riwi-8

N = 212

D_e_ = 67.0 ± 1.1

OD = 30 ± 2

Riwi-7

N = 211

D_e_ = 63.3 ± 1.2

OD = 33 ± 2

Riwi-10

N = 183

D_e_ = 69.3 ± 1.4

OD = 30 ± 2

Riwi-9

N = 185

D_e_ = 64.1 ± 1.4

OD = 34 ± 2


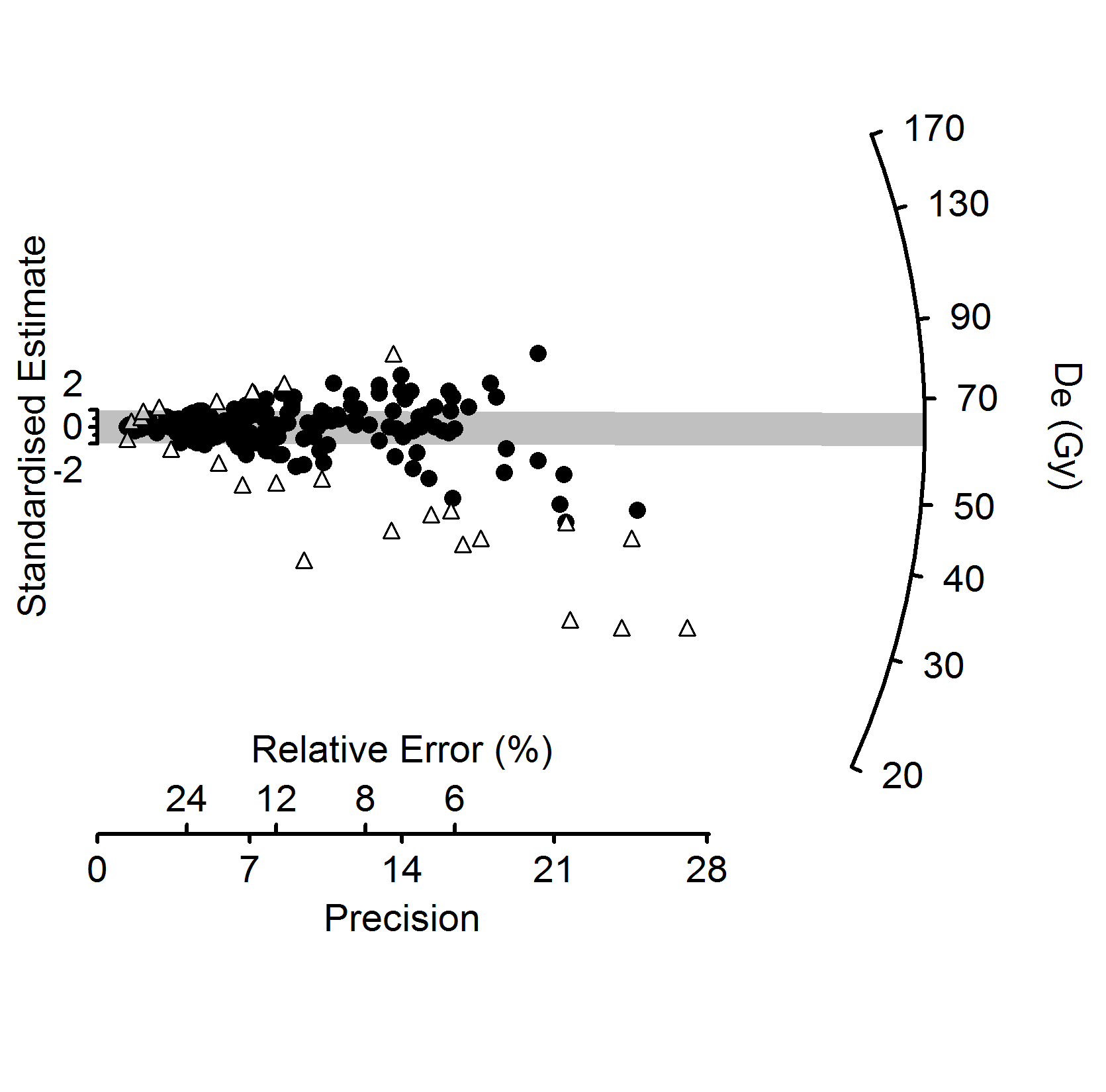

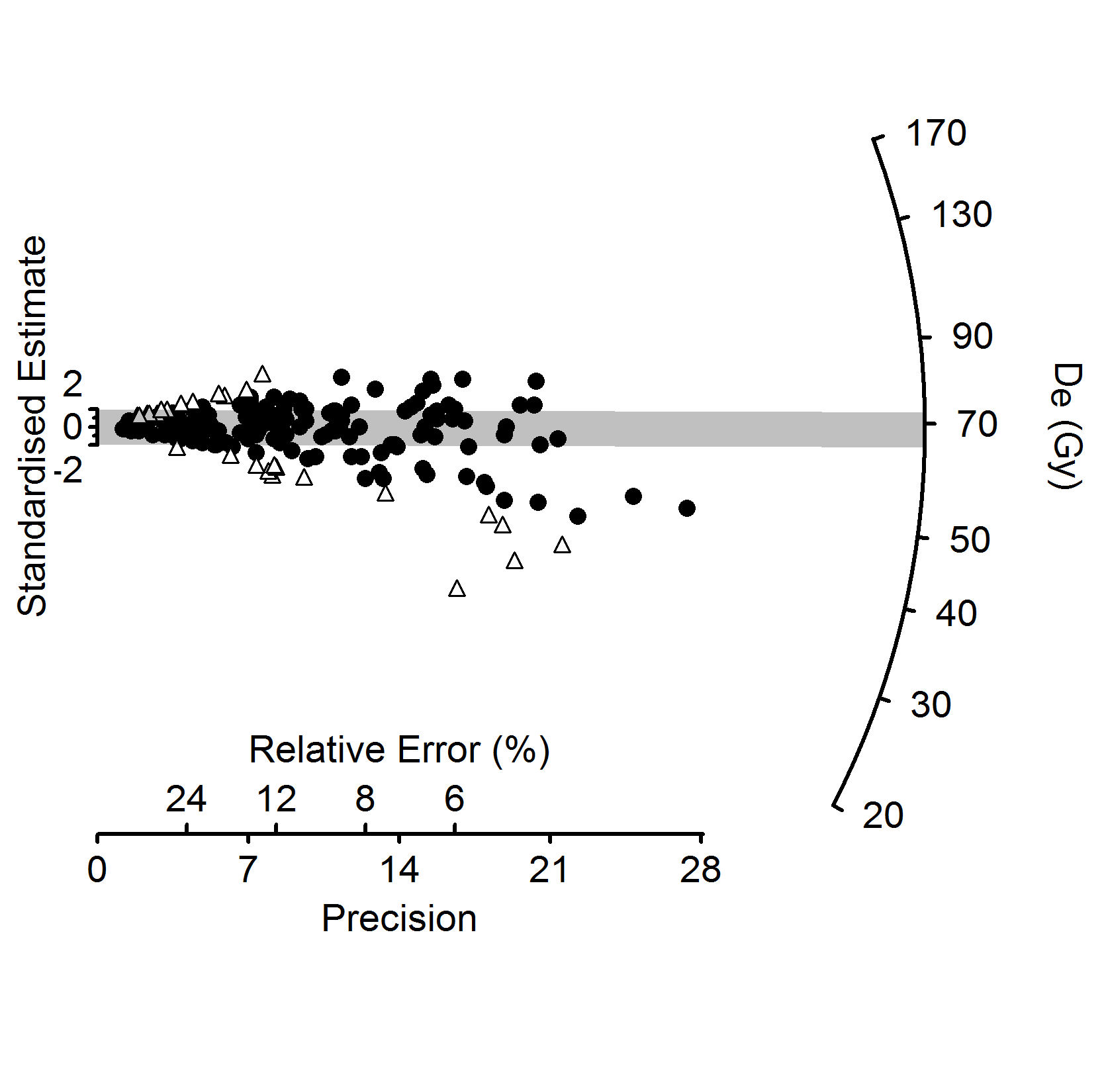


Riwi-11

N = 207

D_e_ = 68.0 ± 1.4

OD = 27 ± 2


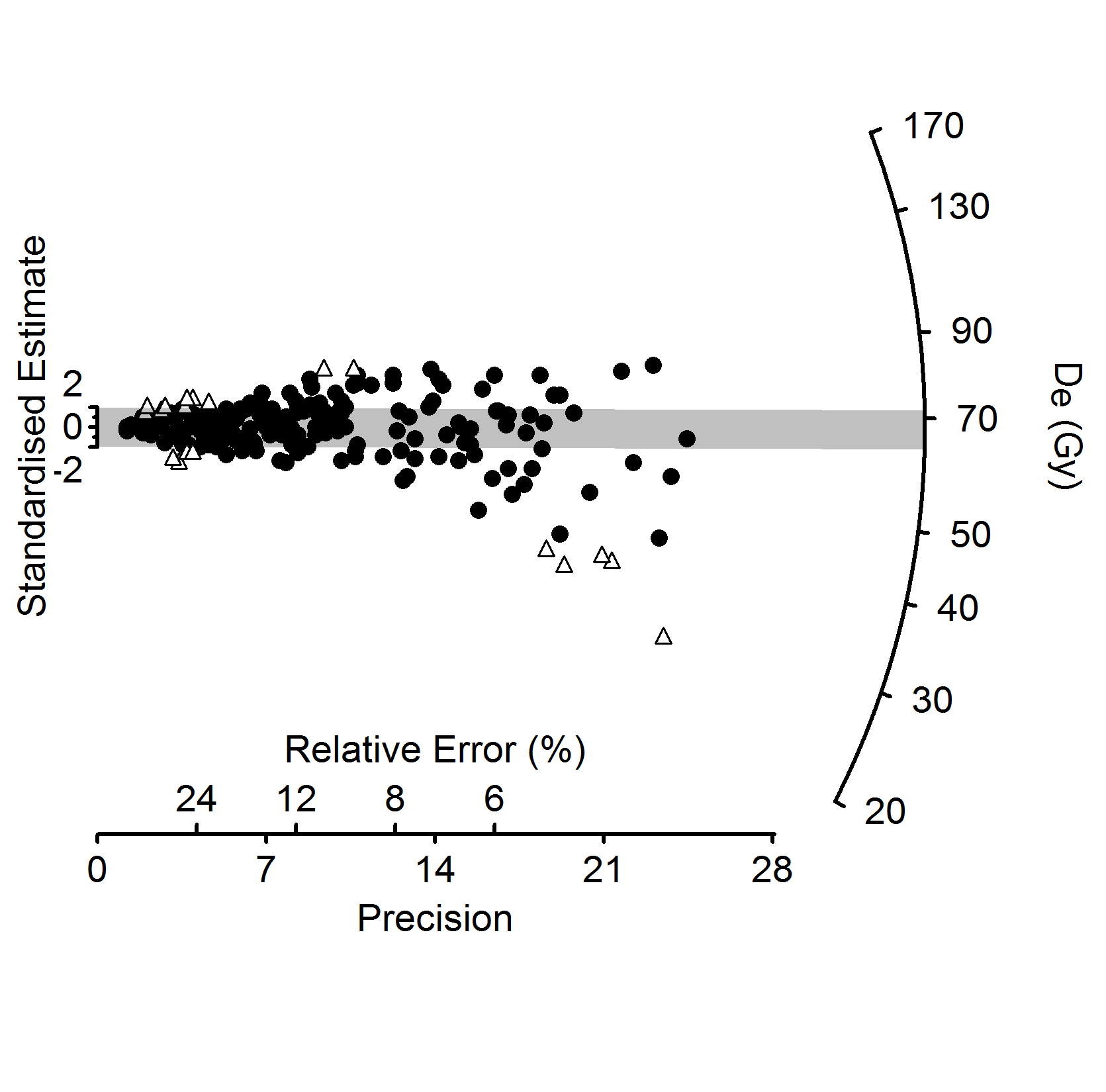

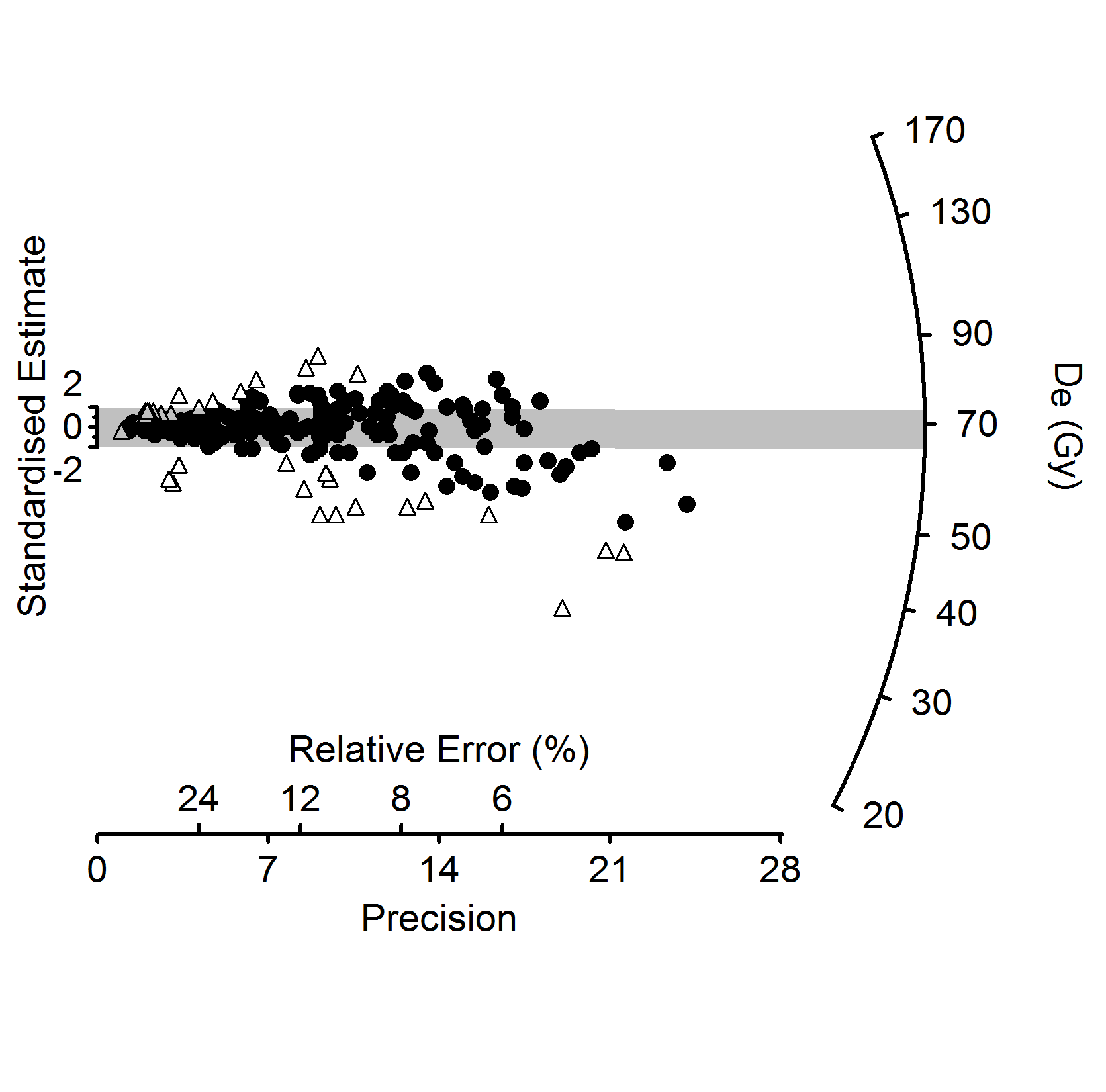


Riwi-12

N = 202

D_e_ = 68.9 ± 1.3

OD = 29 ± 2


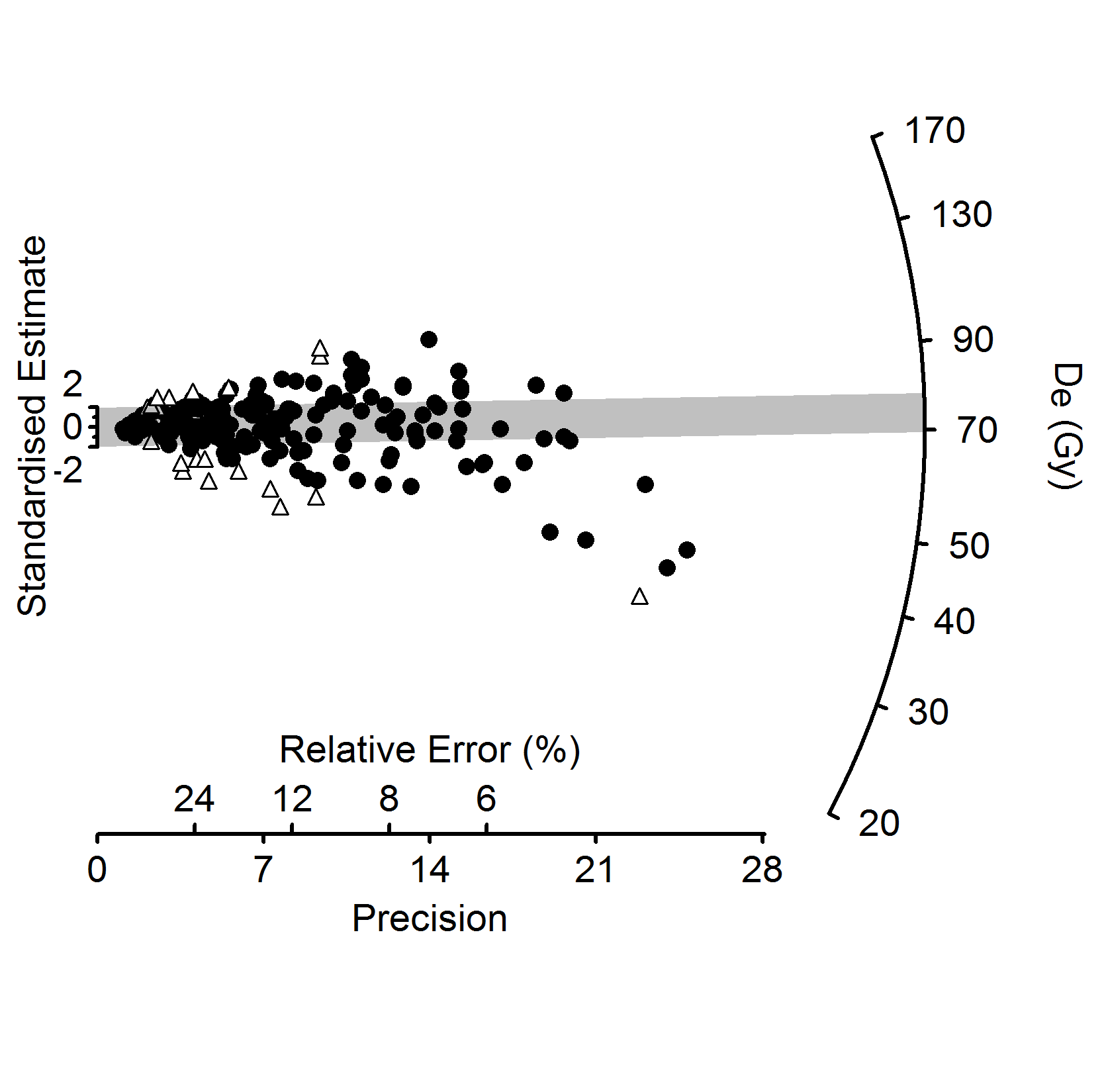

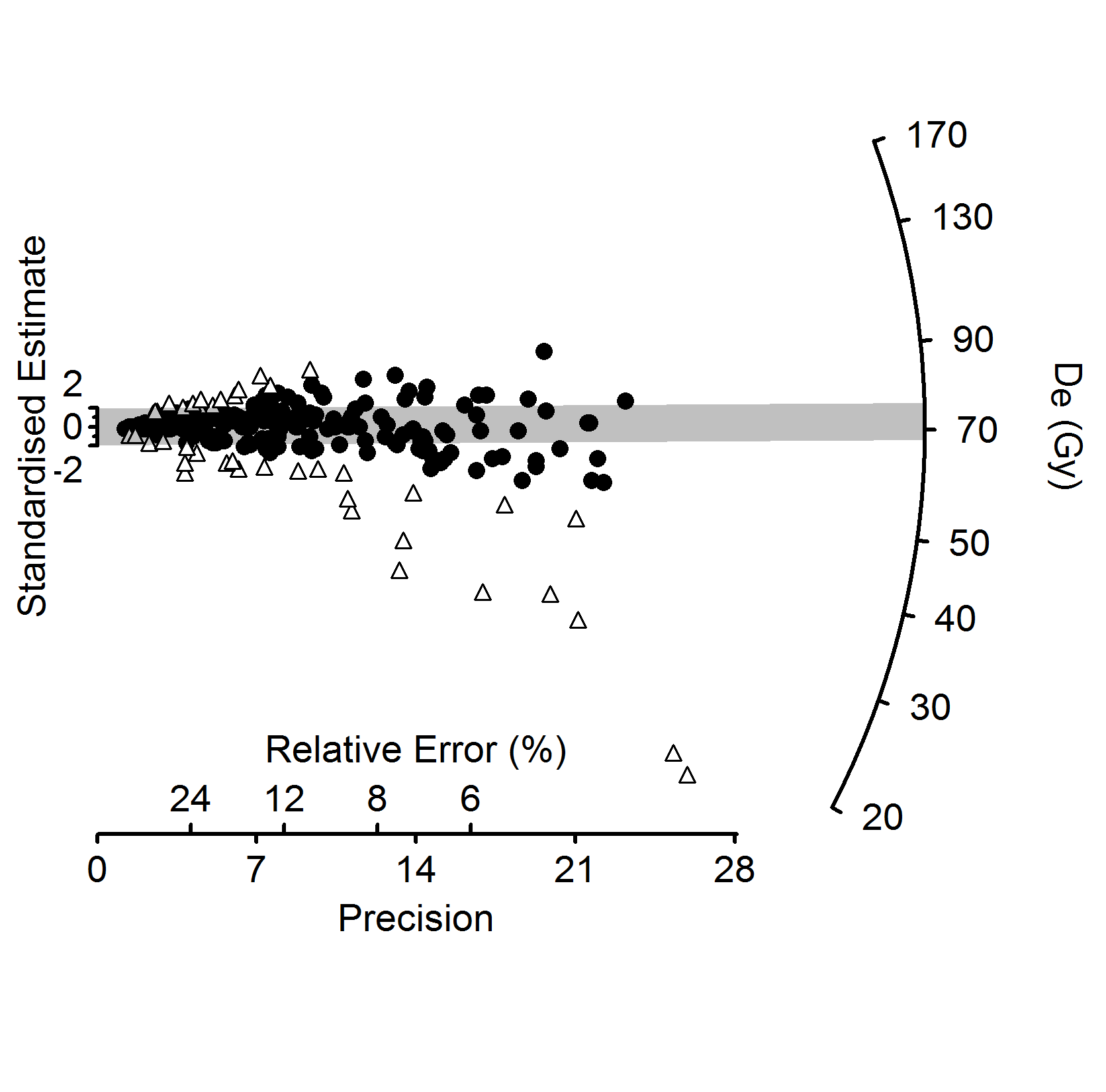


Riwi-14

N = 220

D_e_ = 71.4 ± 1.2

OD = 35 ± 2

Riwi-13

N = 198

D_e_ = 72.9 ± 1.9

OD = 36 ± 2

Riwi-16

N = 182

D_e_ = 68.2 ± 1.3

OD = 29 ± 2

Riwi-15

N = 183

D_e_ = 68.6 ± 1.6

OD = 34 ± 2


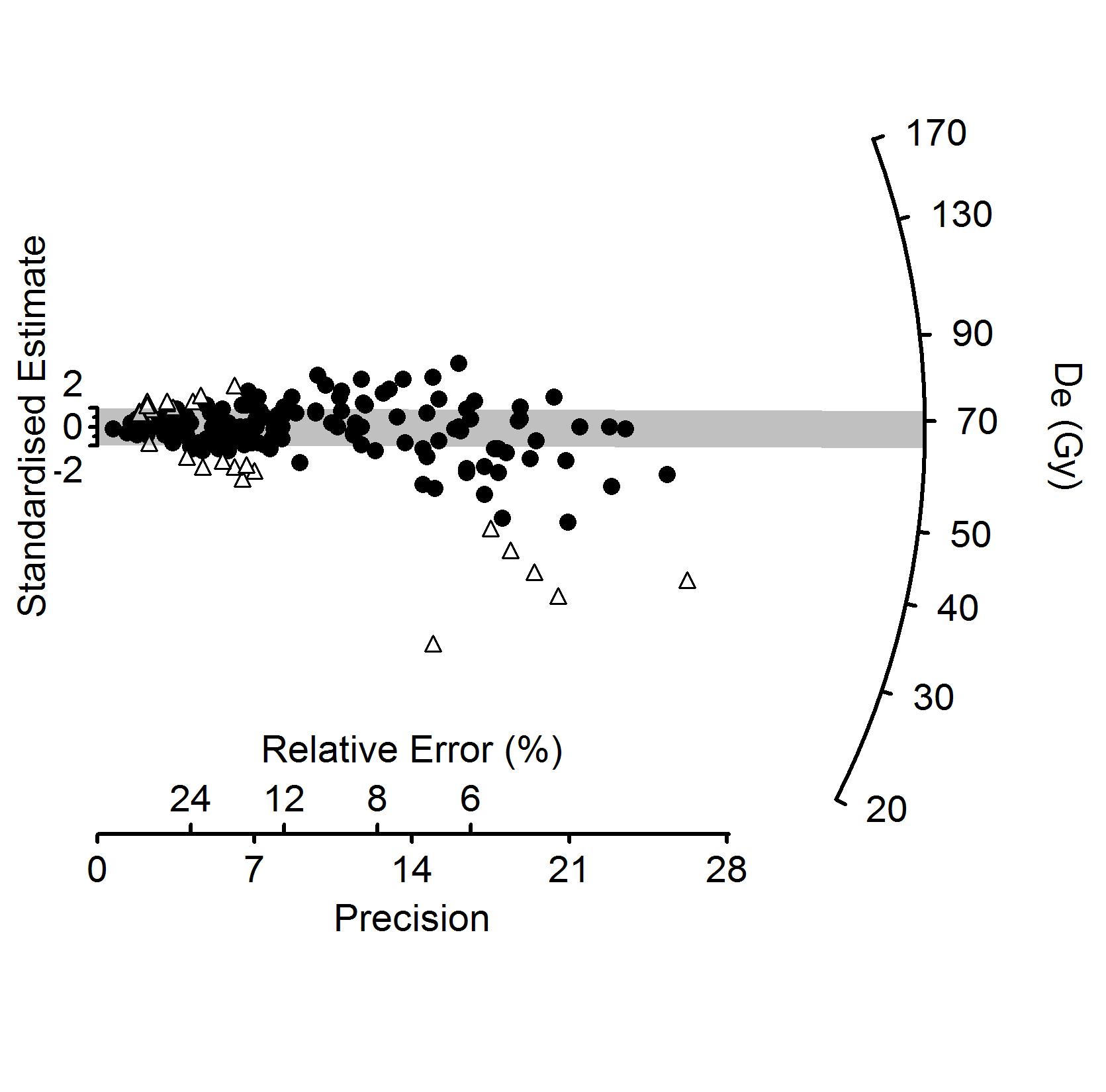

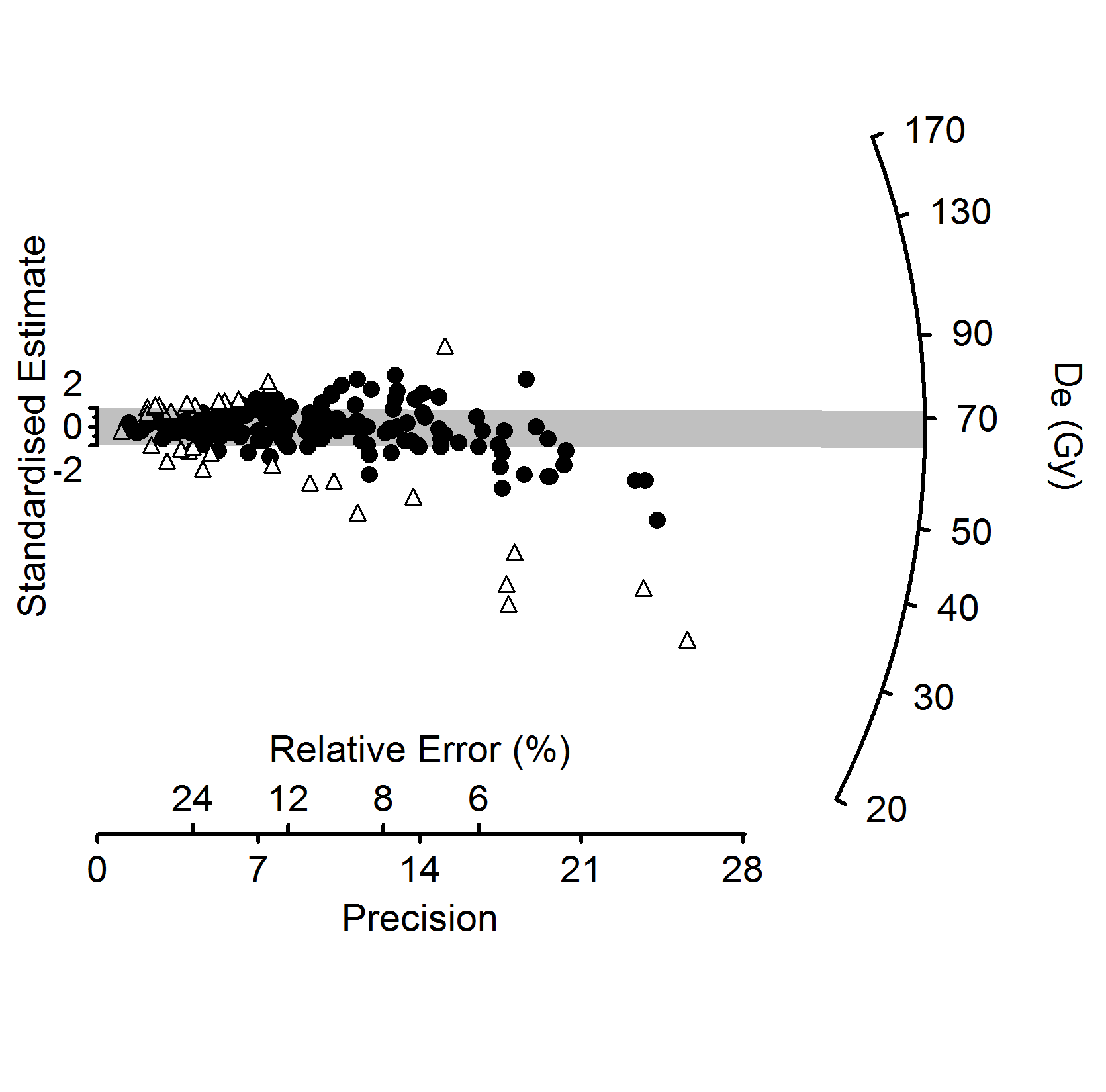


Riwi-17

N = 173

D_e_ = 77.4 ± 1.8

OD = 33 ± 2

Riwi-18

N = 185

D_e_ = 70.9 ± 1.5

OD = 30 ± 2


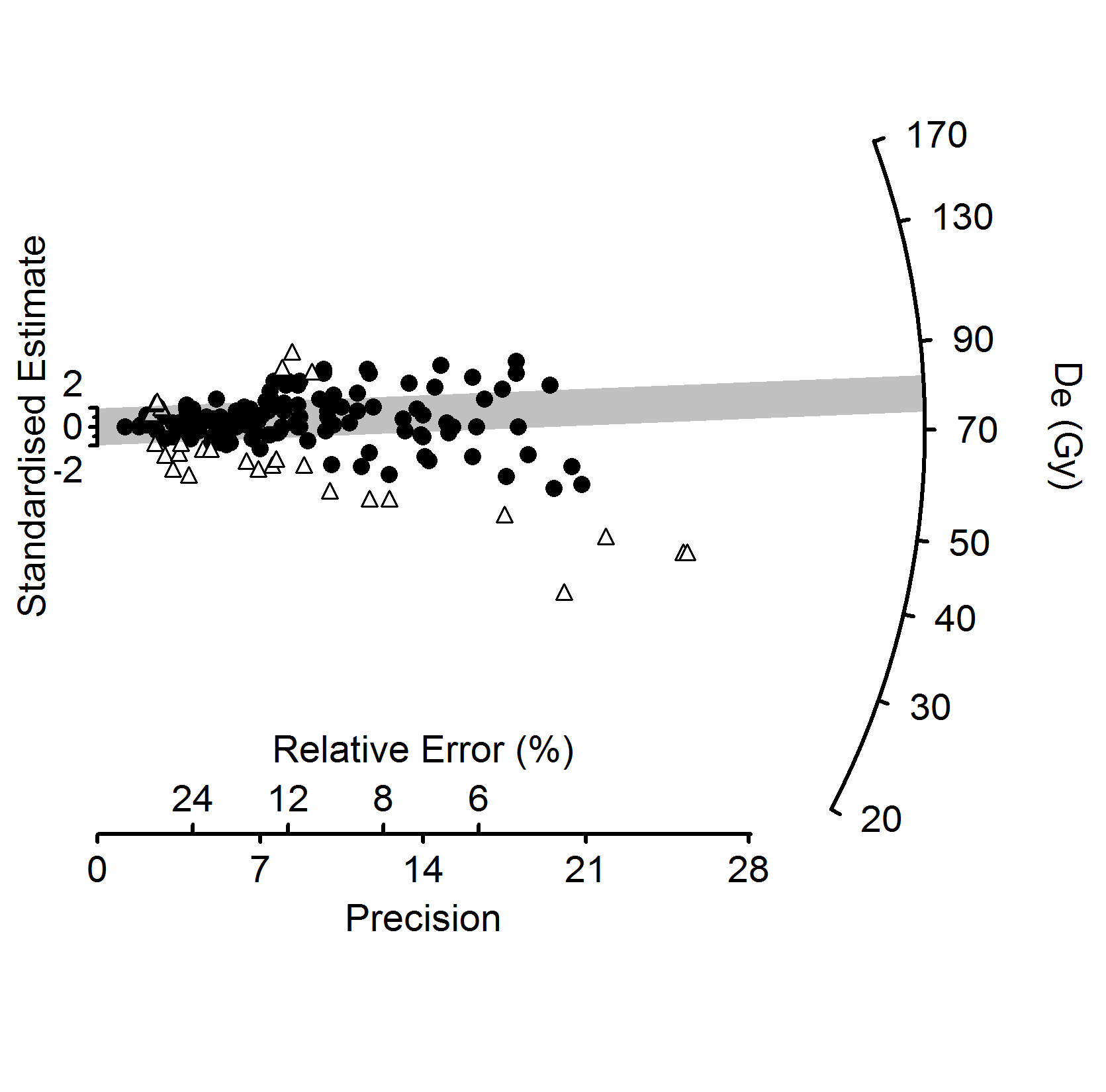

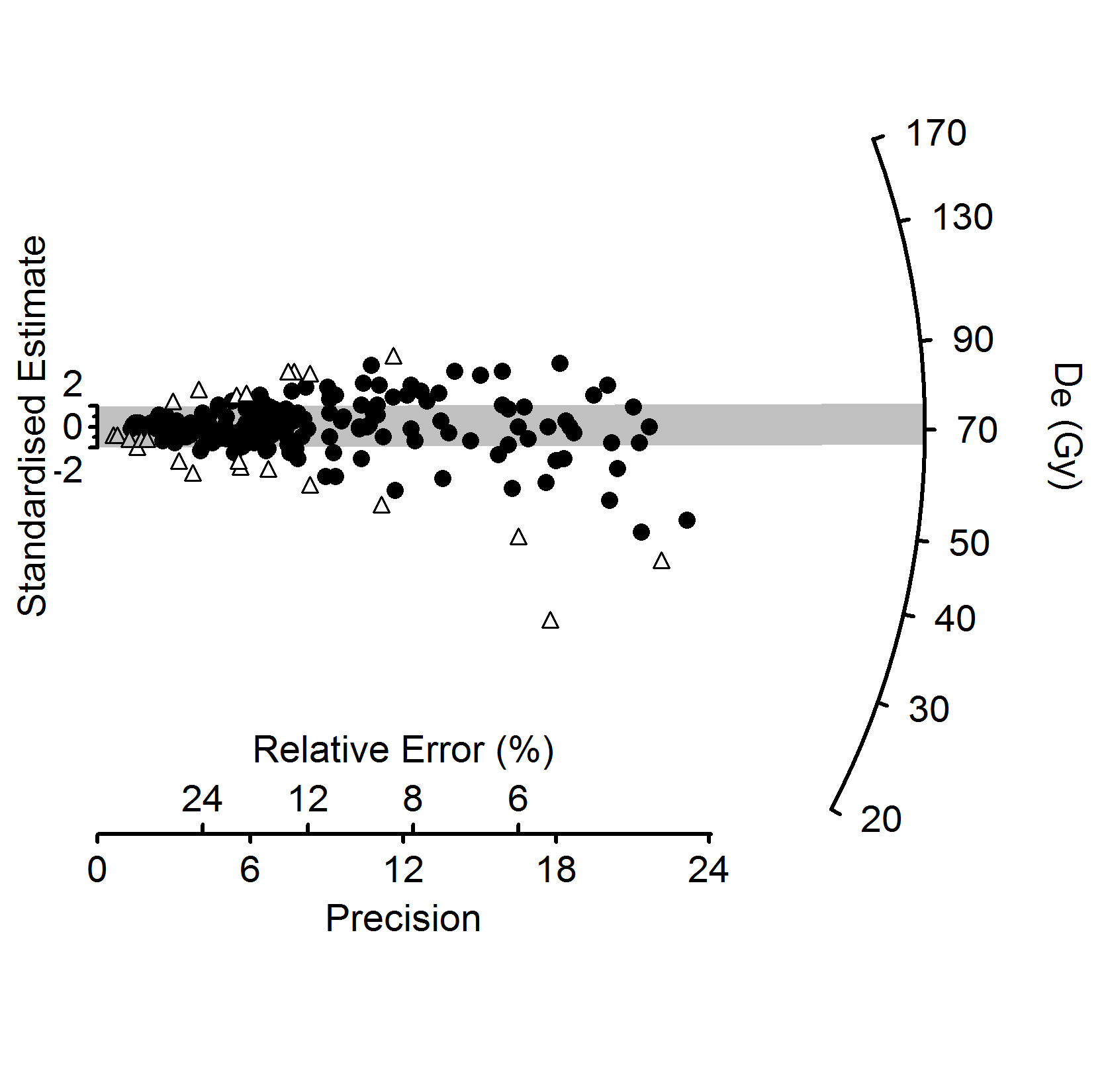


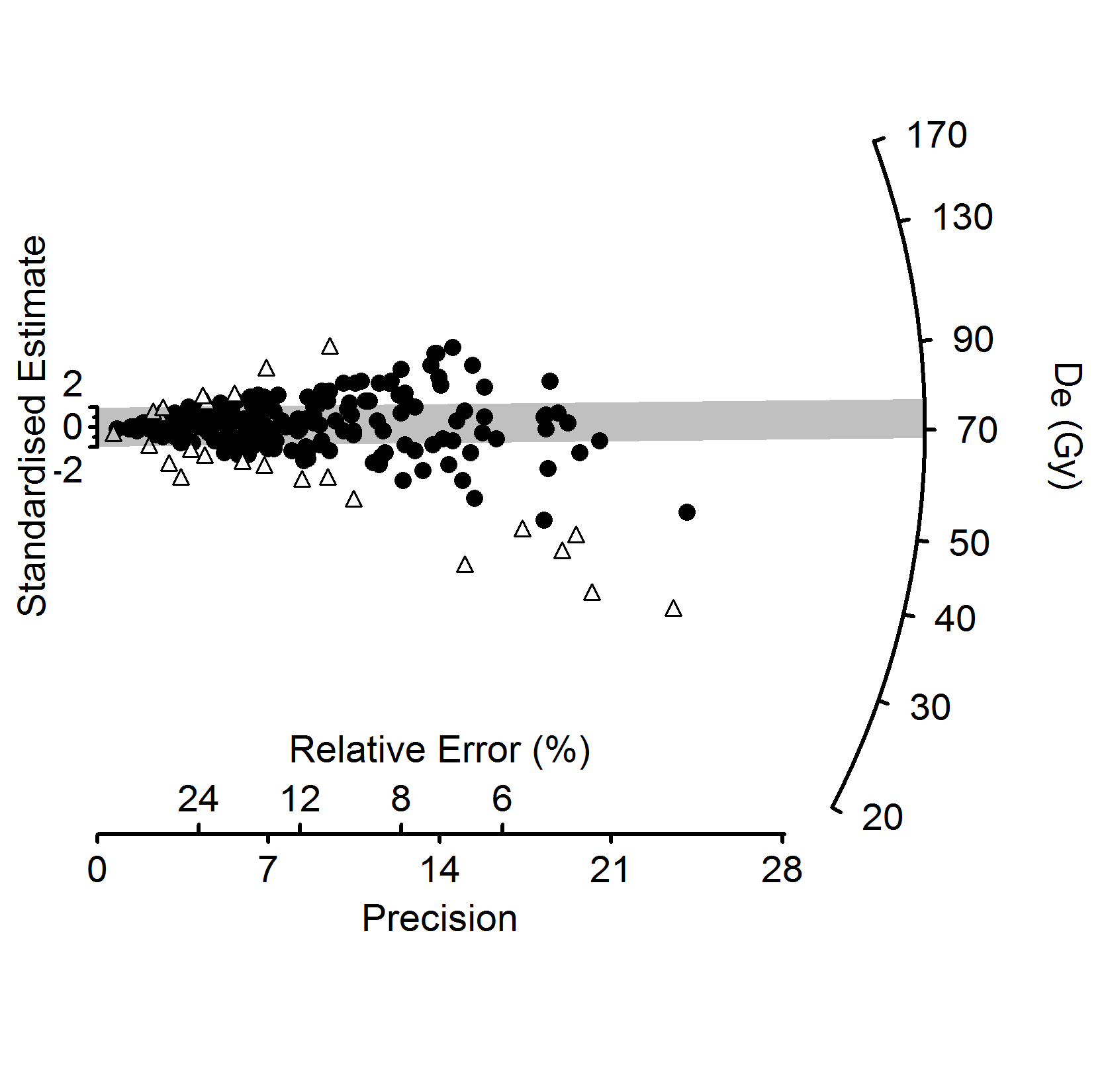

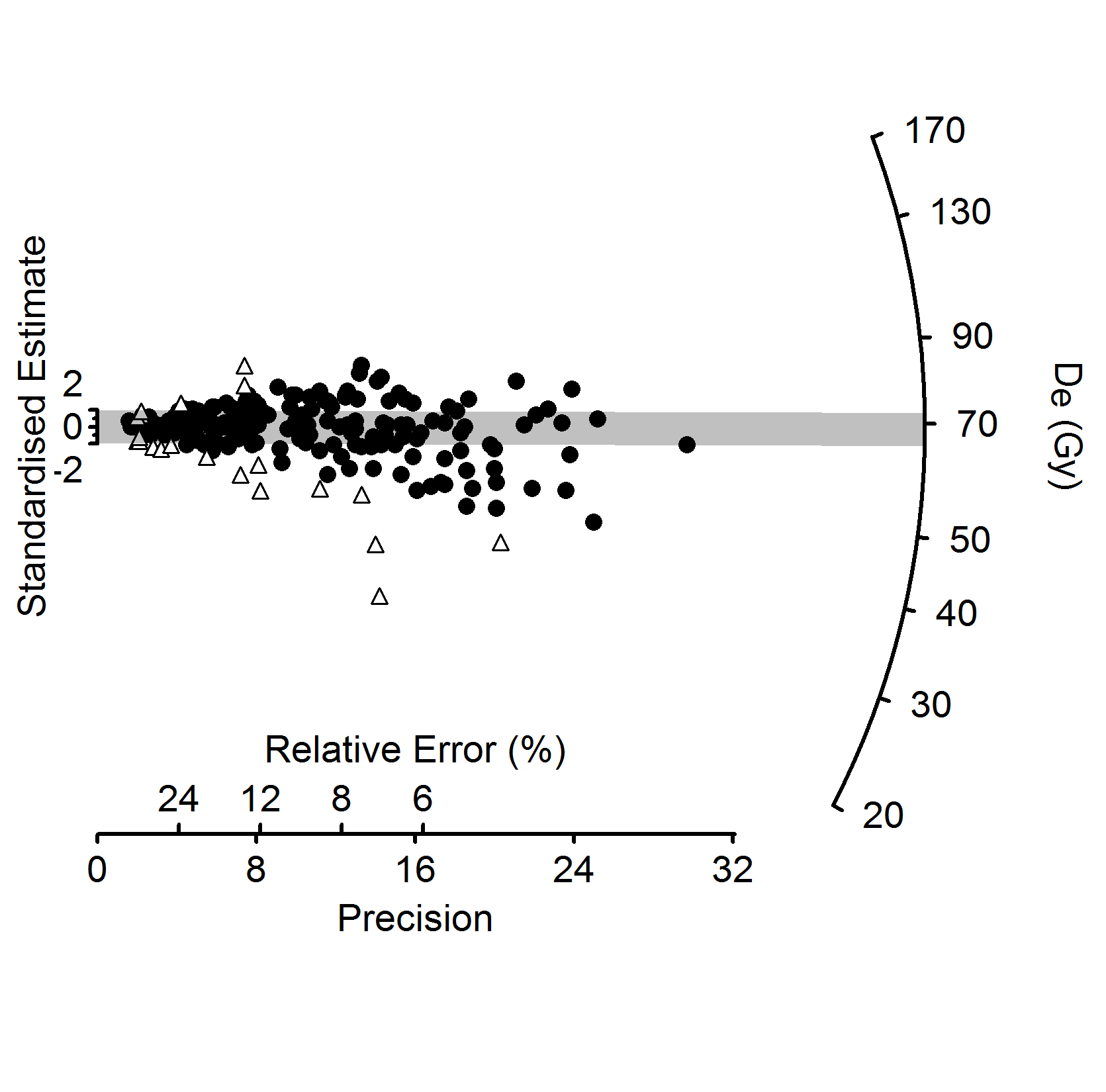


Riwi-20

N = 203

D_e_ = 69.2 ± 1.4

OD = 31 ± 2

Riwi-19

N = 214

D_e_ = 72.3 ± 1.5

OD = 30 ± 2

Riwi-22

N = 178

D_e_ = 77.5 ± 1.8

OD = 35 ± 2

Riwi-21

N = 174

D_e_ = 73.0 ± 2.2

OD = 35 ± 2


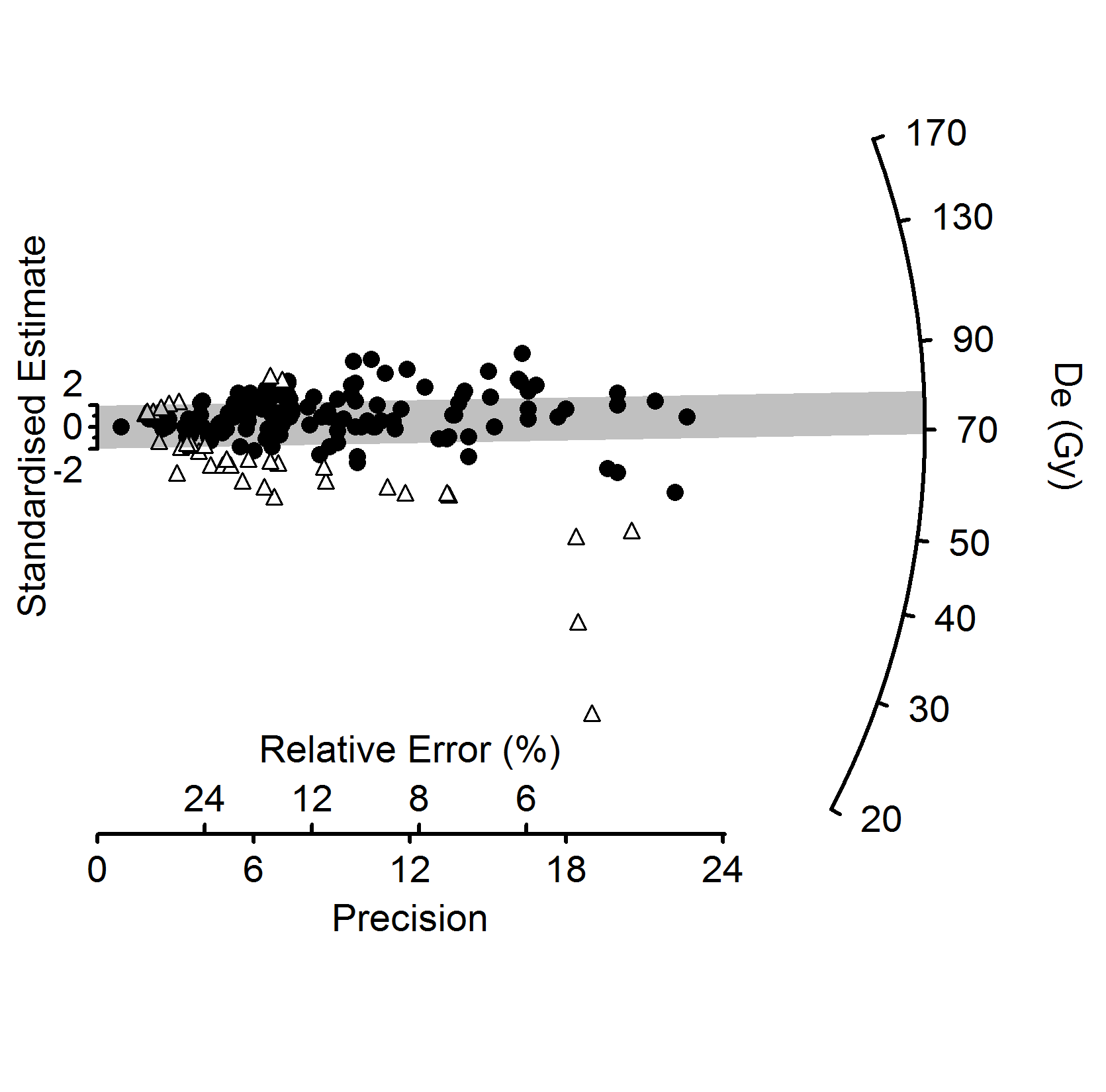

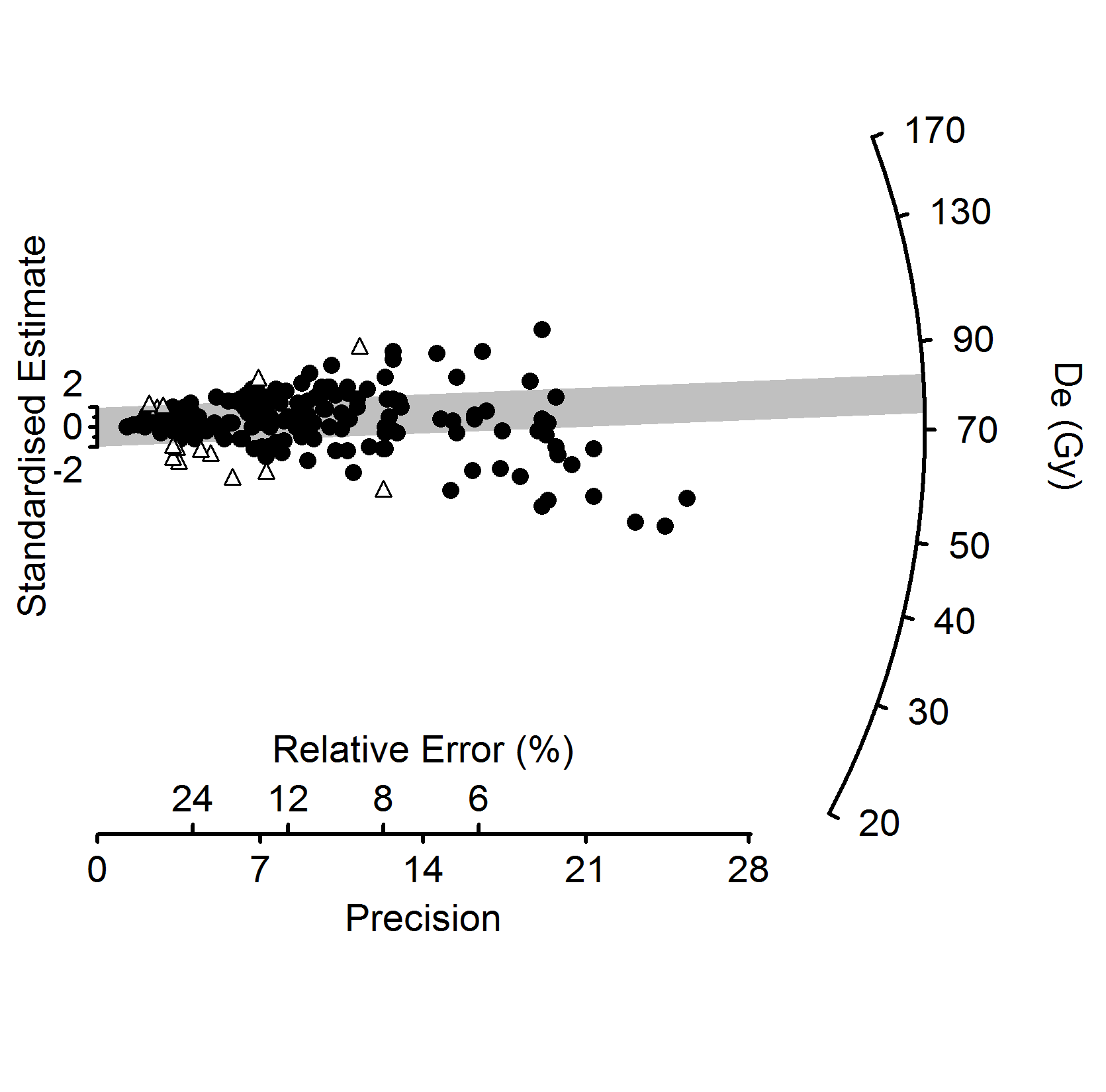


Riwi-24

N = 258

D_e_ = 67.7 ± 1.2

OD = 34 ± 2

Riwi-23

N = 208

D_e_ = 73.4 ± 1.8

OD = 32 ± 2


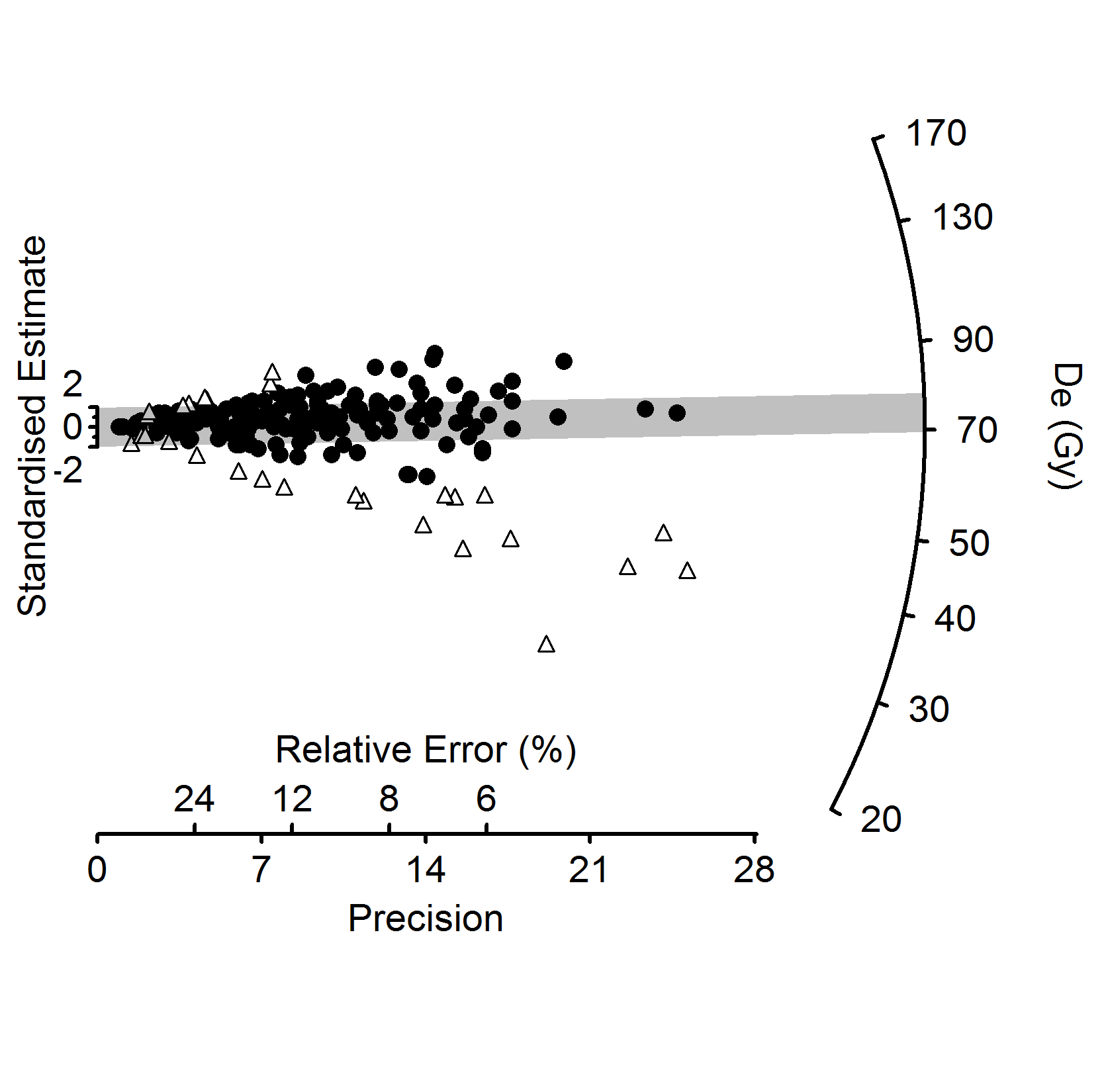

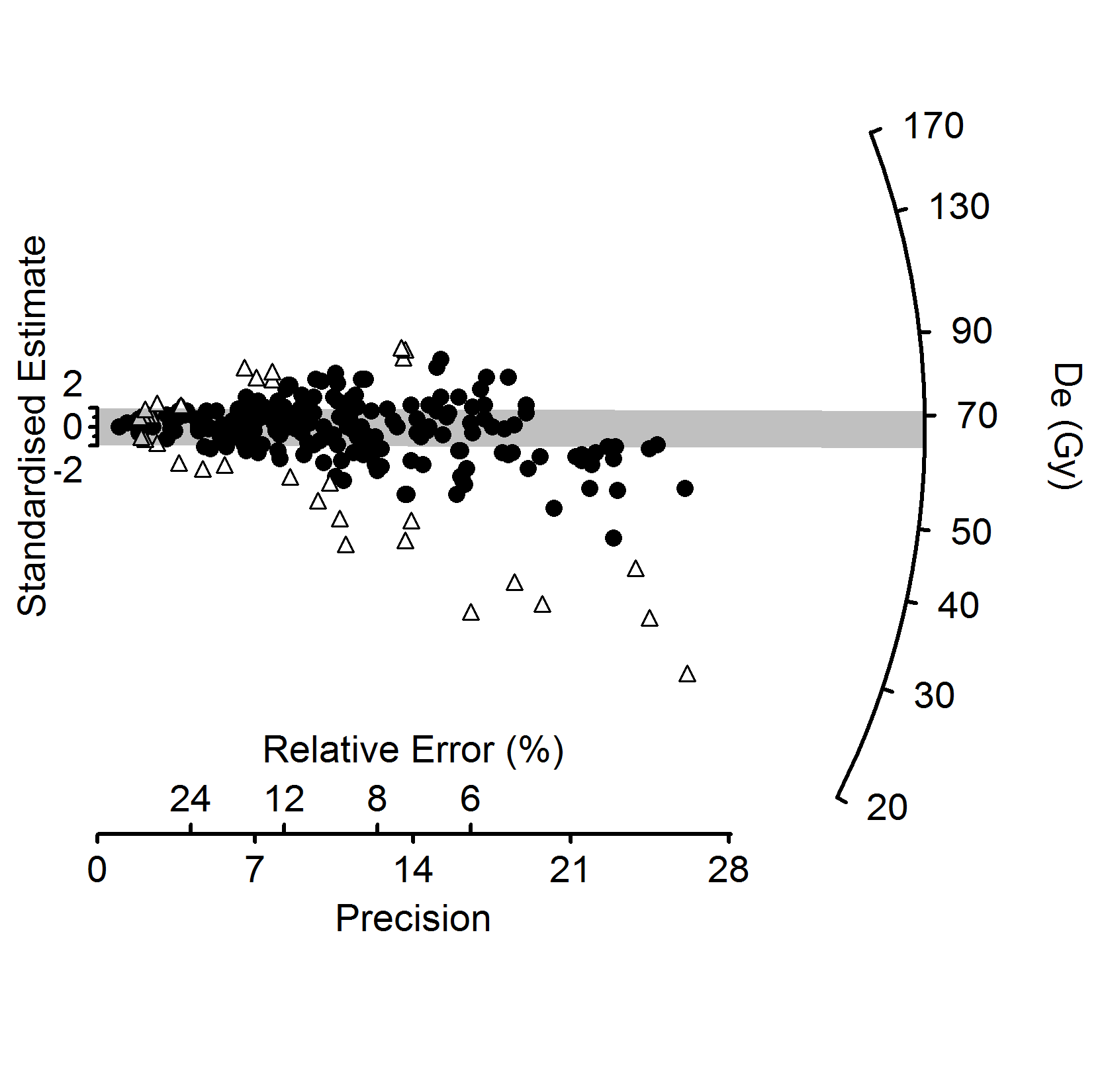


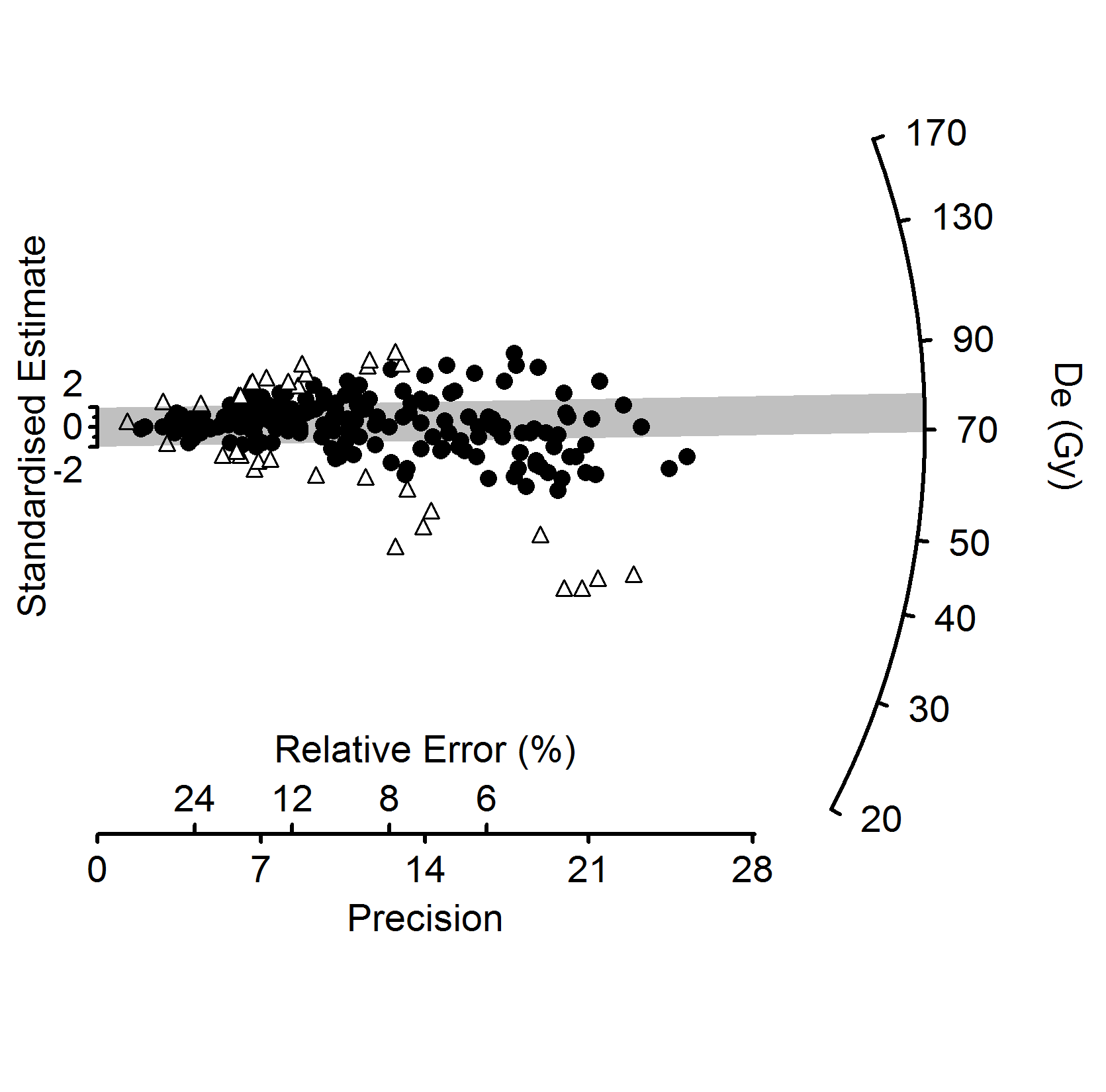

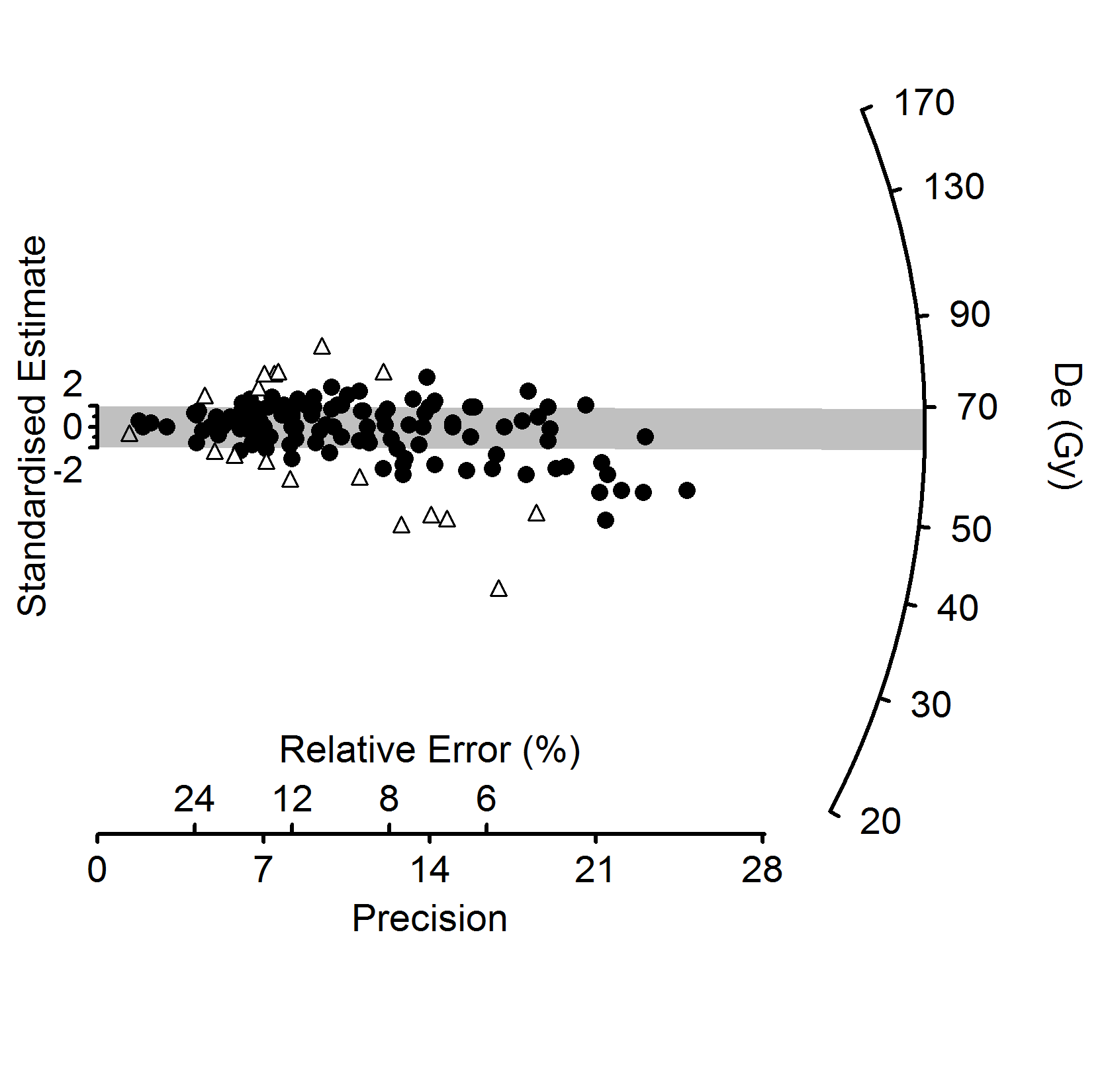


Riwi-26

N = 135

D_e_ = 66.2 ± 1.3

OD = 29 ± 2

Riwi-25

N = 217

D_e_ = 72.9 ± 1.2

OD = 28 ± 2

Riwi-28

N = 141

D_e_ = 71.6 ± 1.8

OD = 35 ± 2

Riwi-27

N = 142

D_e_ = 68.2 ± 1.5

OD = 27 ± 2


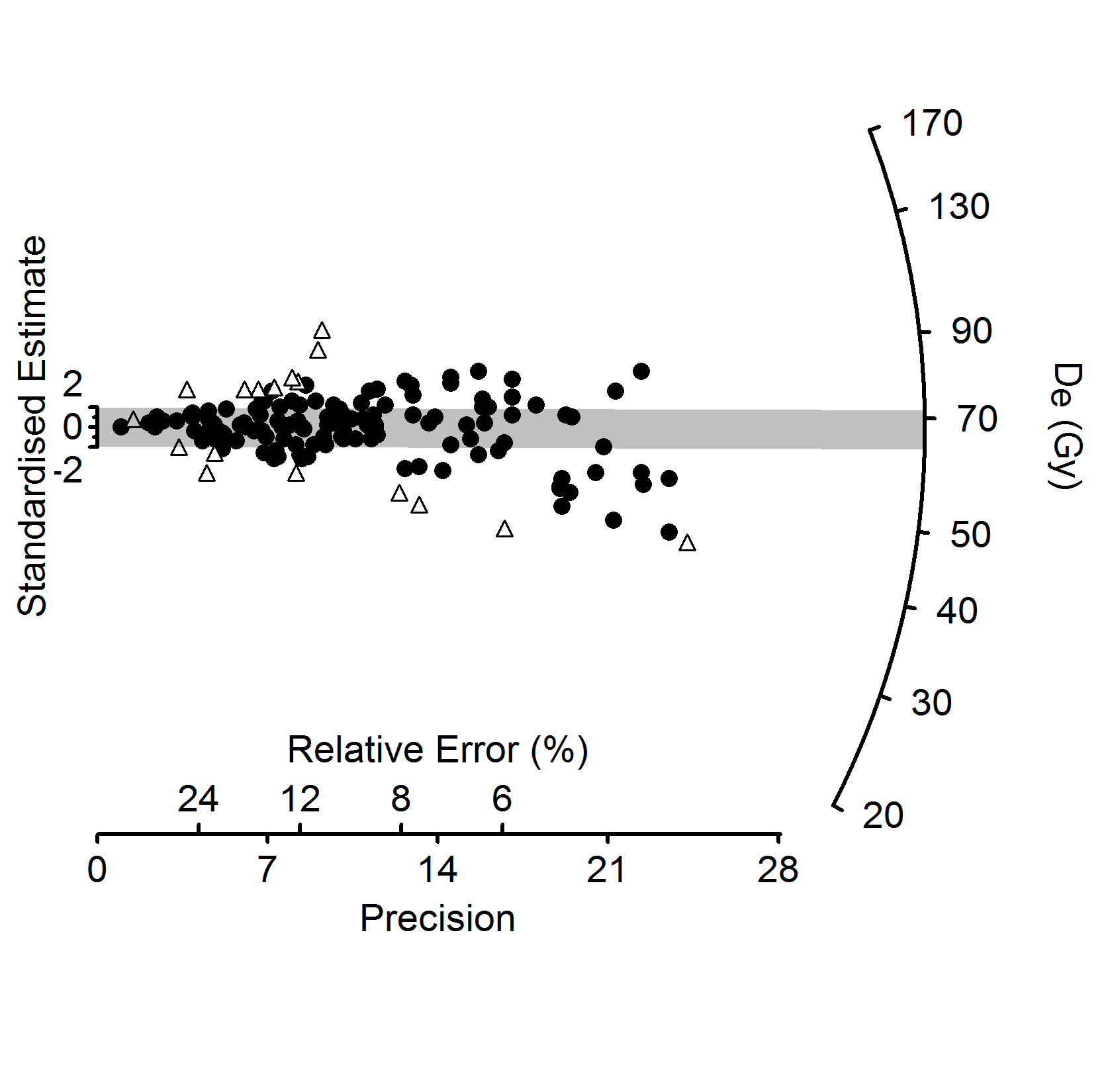

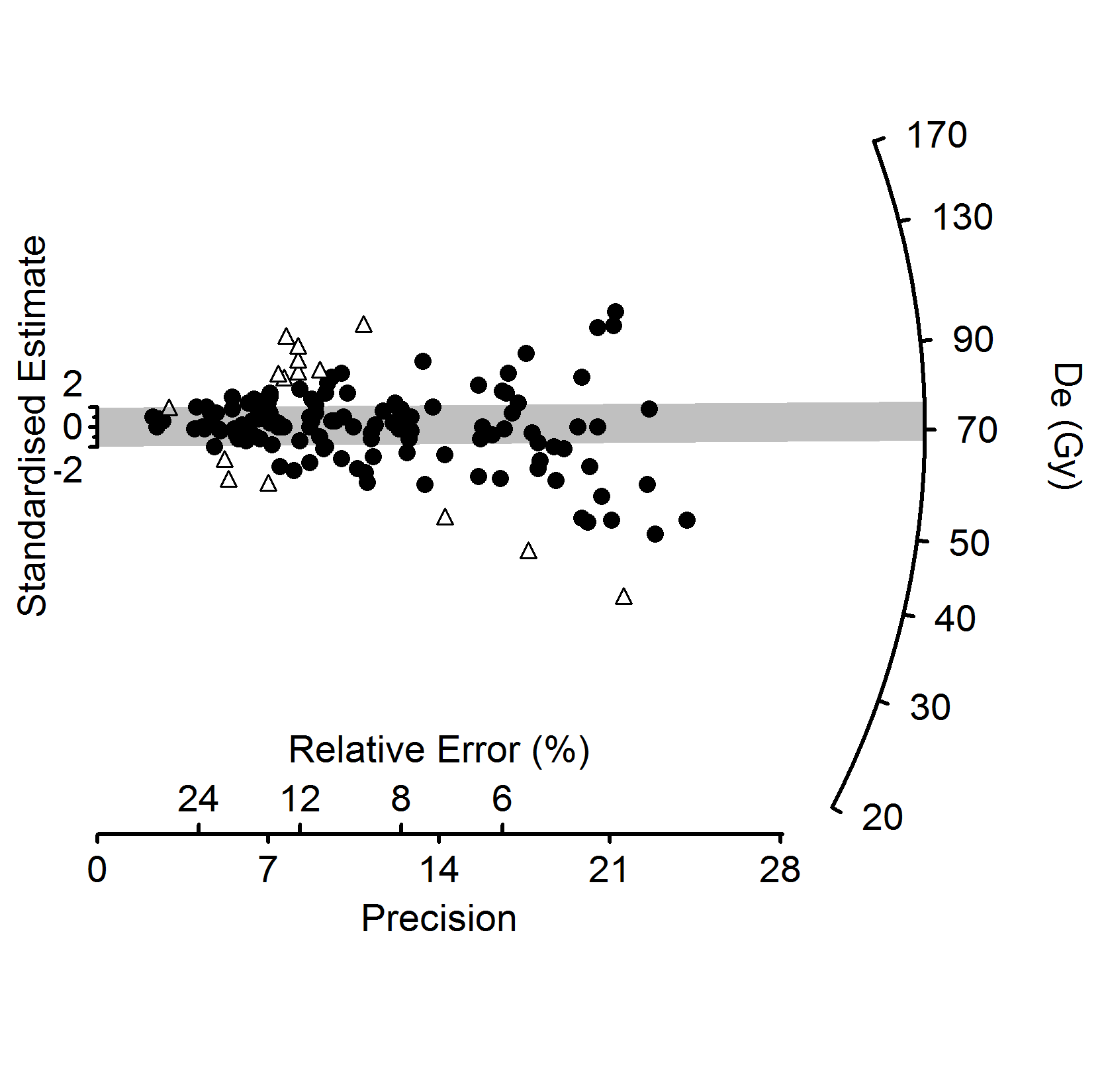


Riwi-29

N = 130

D_e_ = 76.8 ± 2.1

OD = 37 ± 3

Riwi-30

N = 158

D_e_ = 74.0 ± 1.5

OD = 29 ± 2


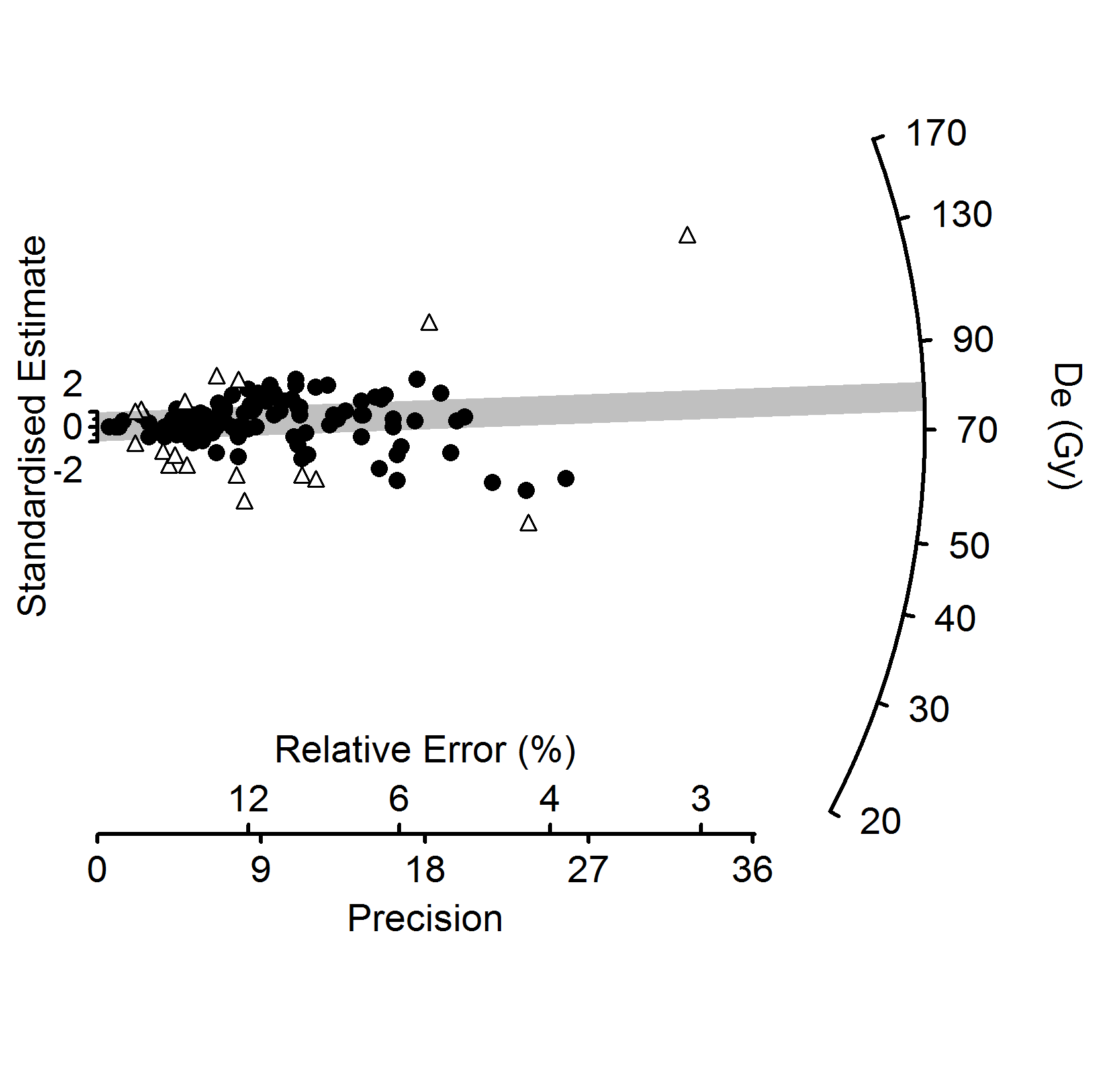

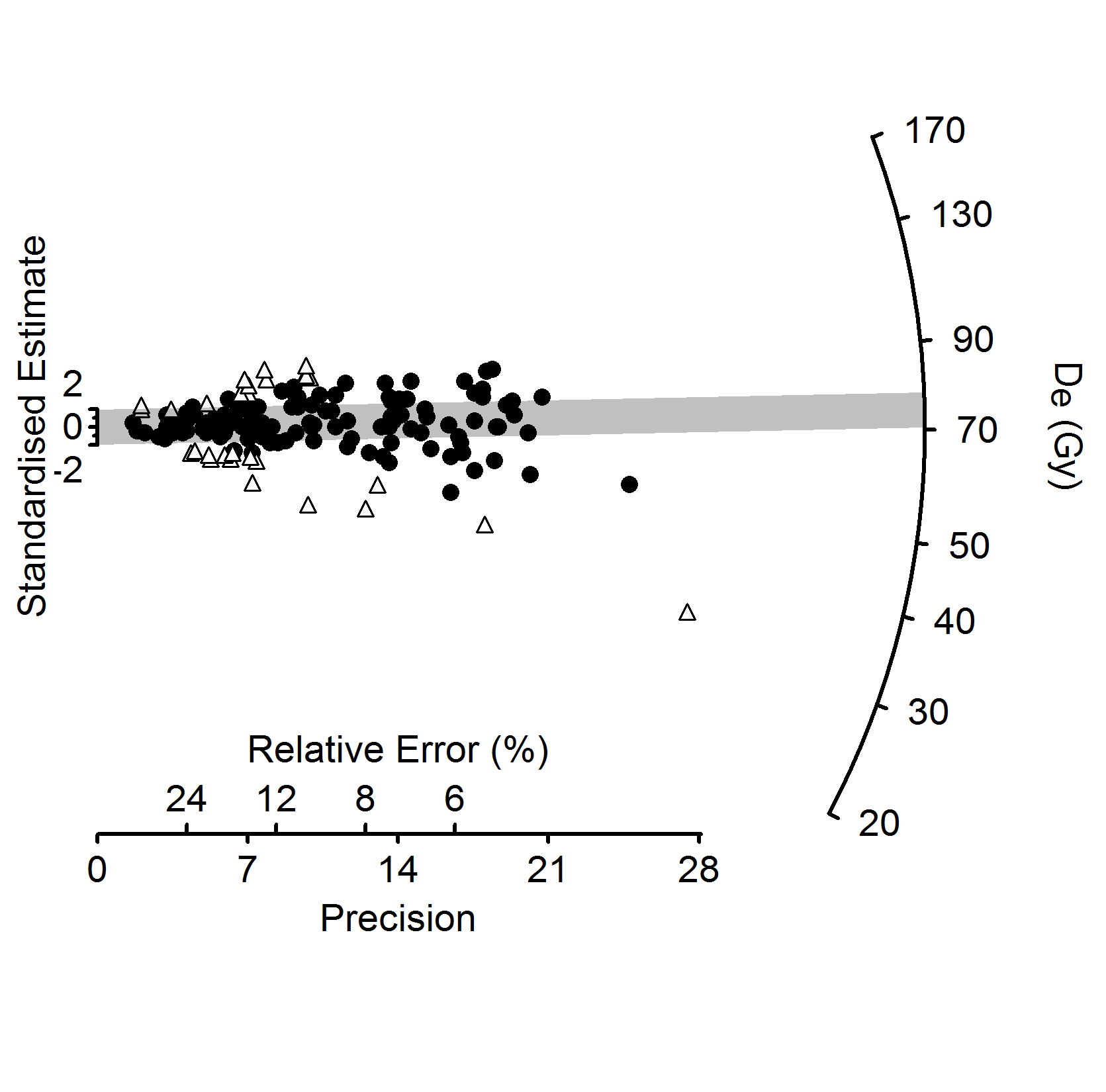


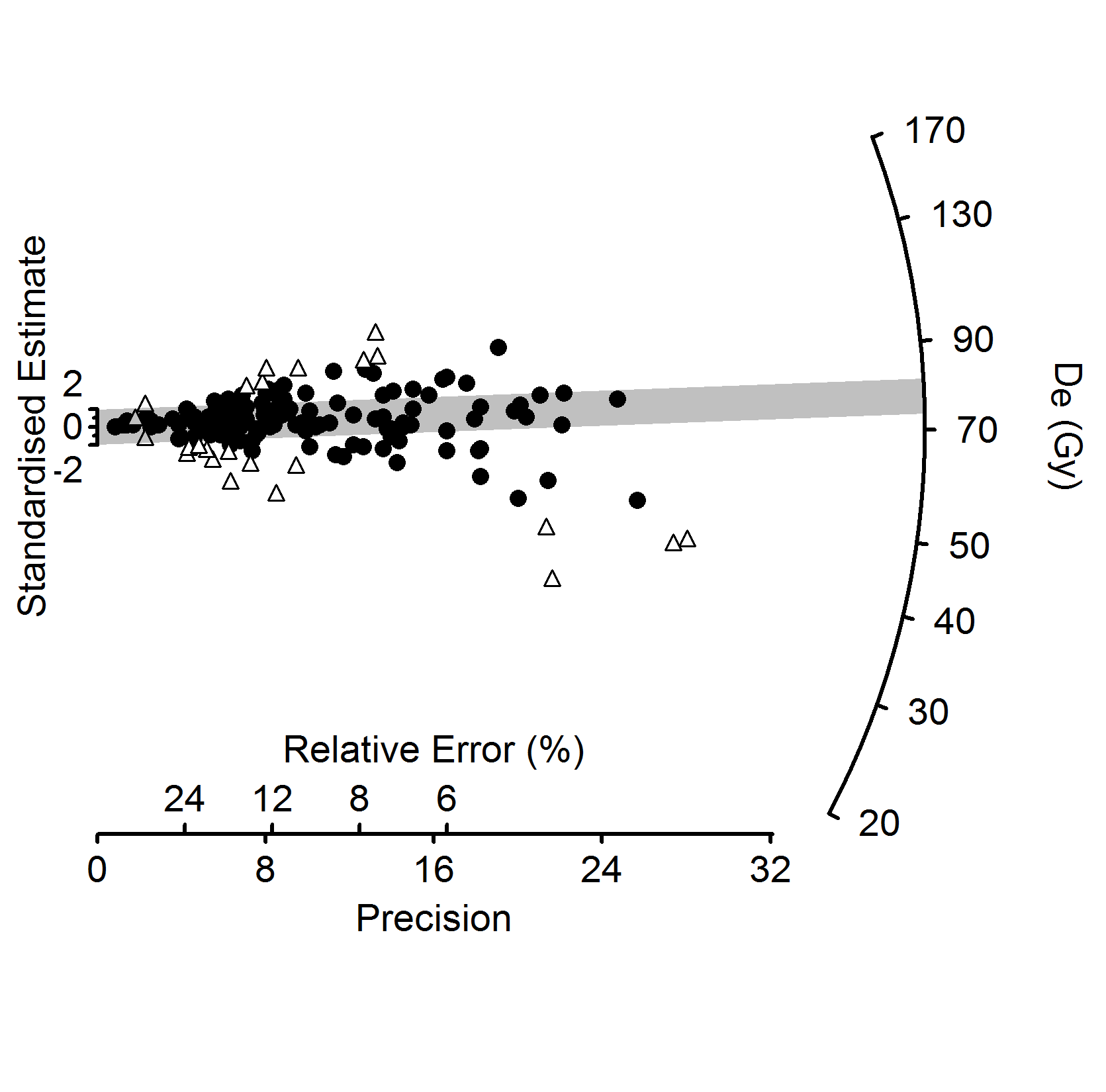

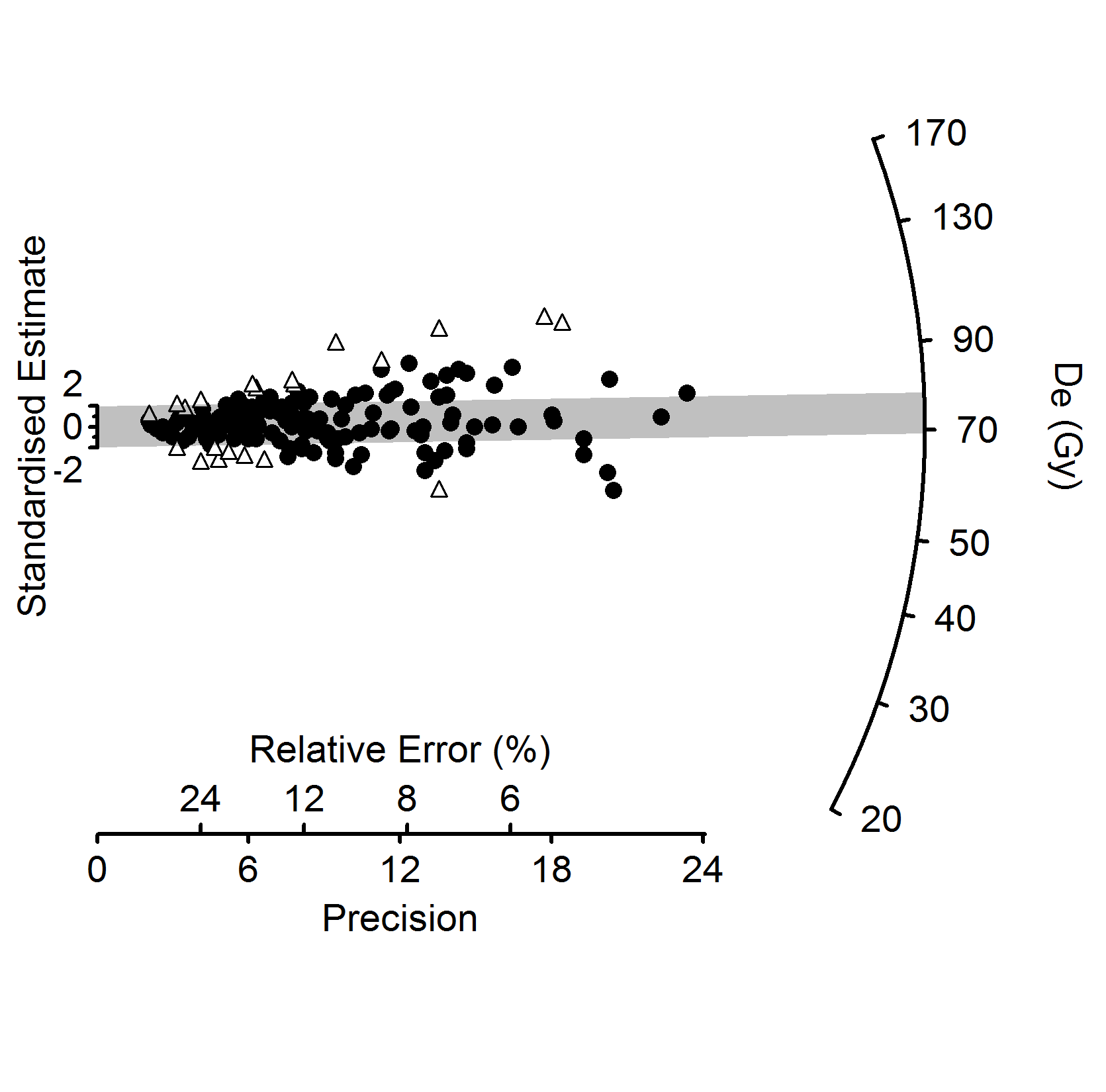


Riwi-32

N = 156

D_e_ = 73.3 ± 1.5

OD = 24 ± 2

Riwi-31

N = 160

D_e_ = 76.6 ± 1.6

OD = 32 ± 2

Riwi-33

N = 136

D_e_ = 78.5 ± 1.9

OD = 34 ± 3

Riwi-34

N = 140

D_e_ = 69.4 ± 1.9

OD = 42 ± 3


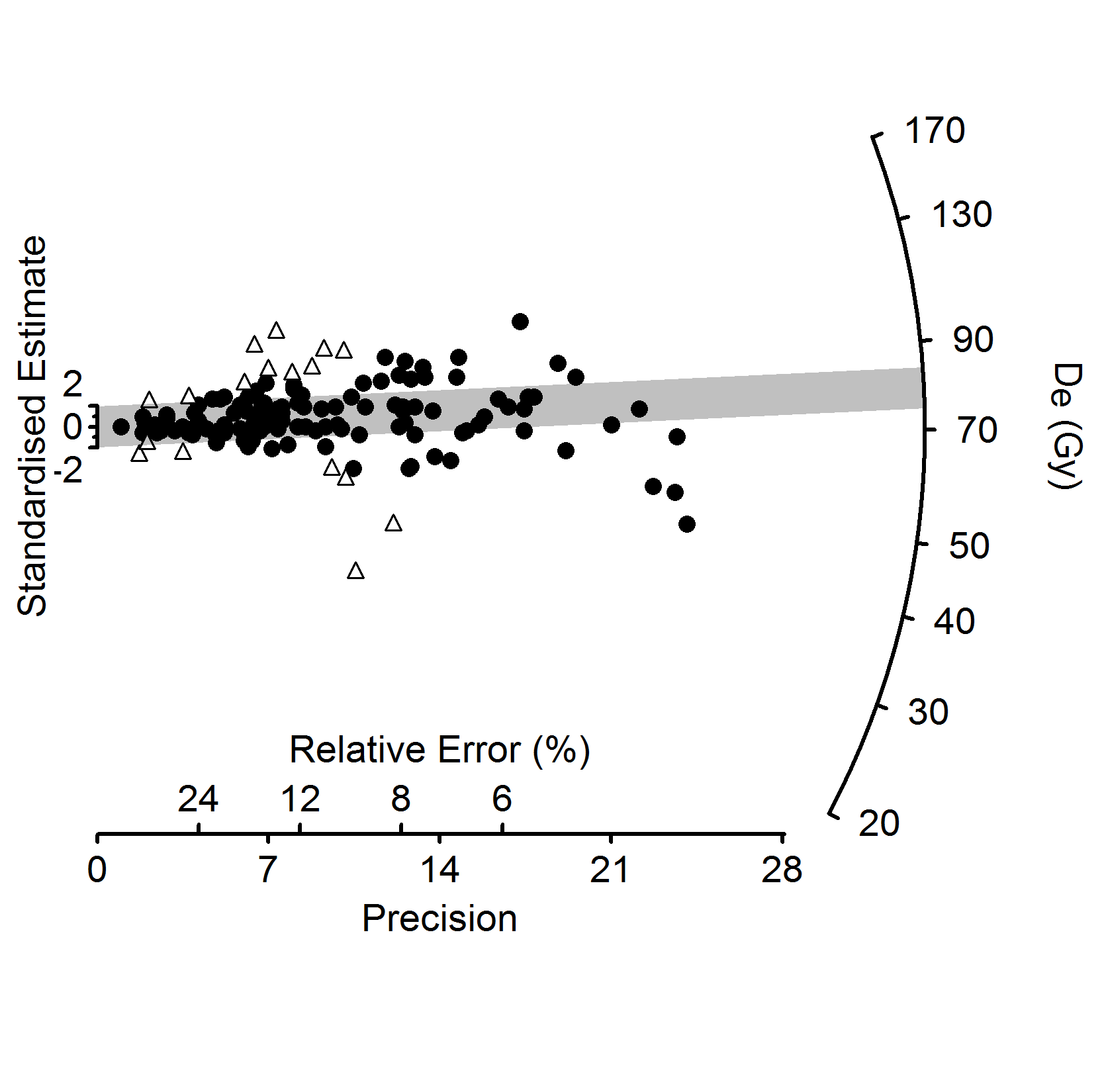

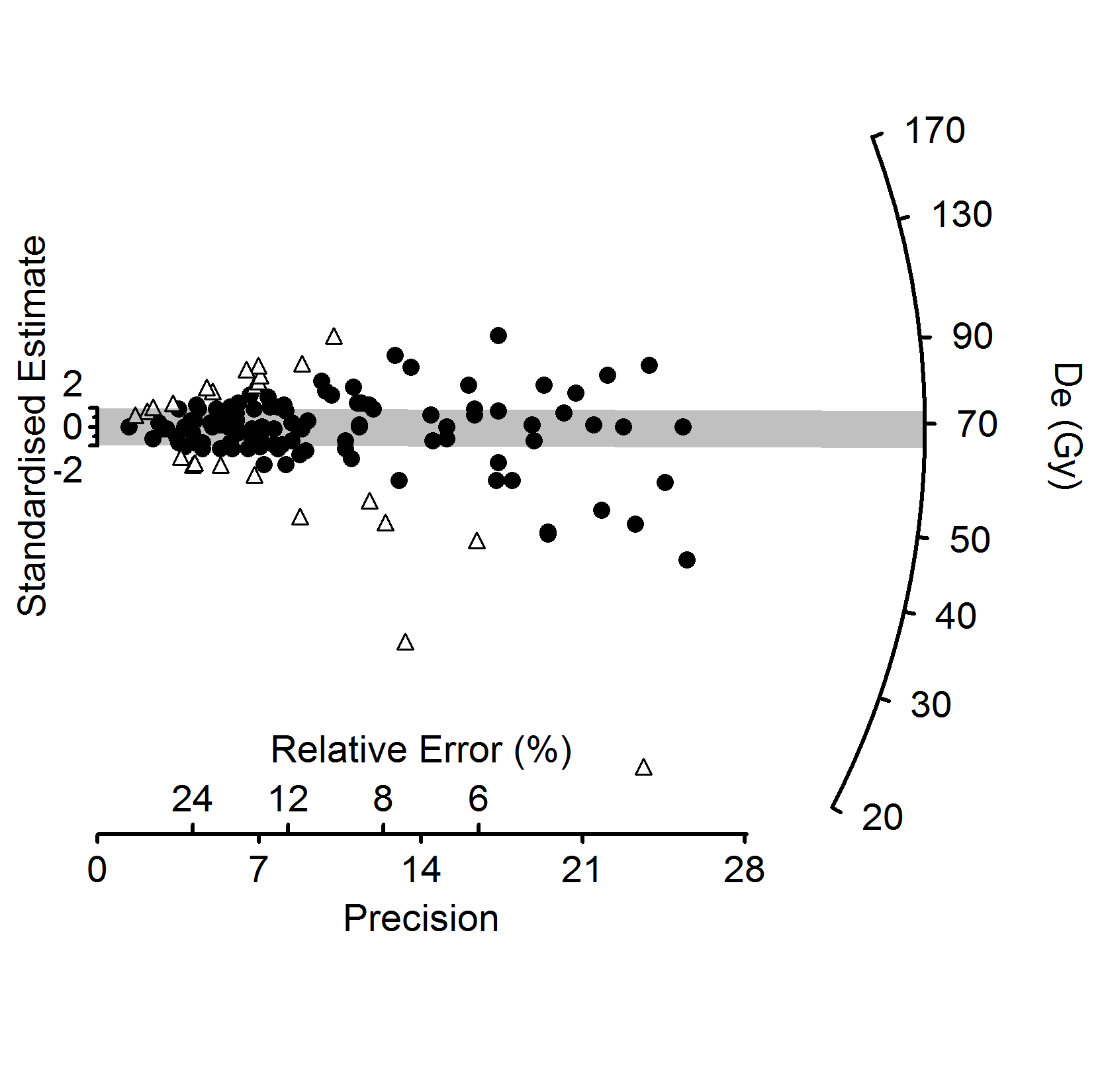


Riwi-35

N = 117

D_e_ = 75.8 ± 2.3

OD = 29 ± 2

Riwi-36

N = 130

D_e_ = 84.6 ± 1.8

OD = 29 ± 2


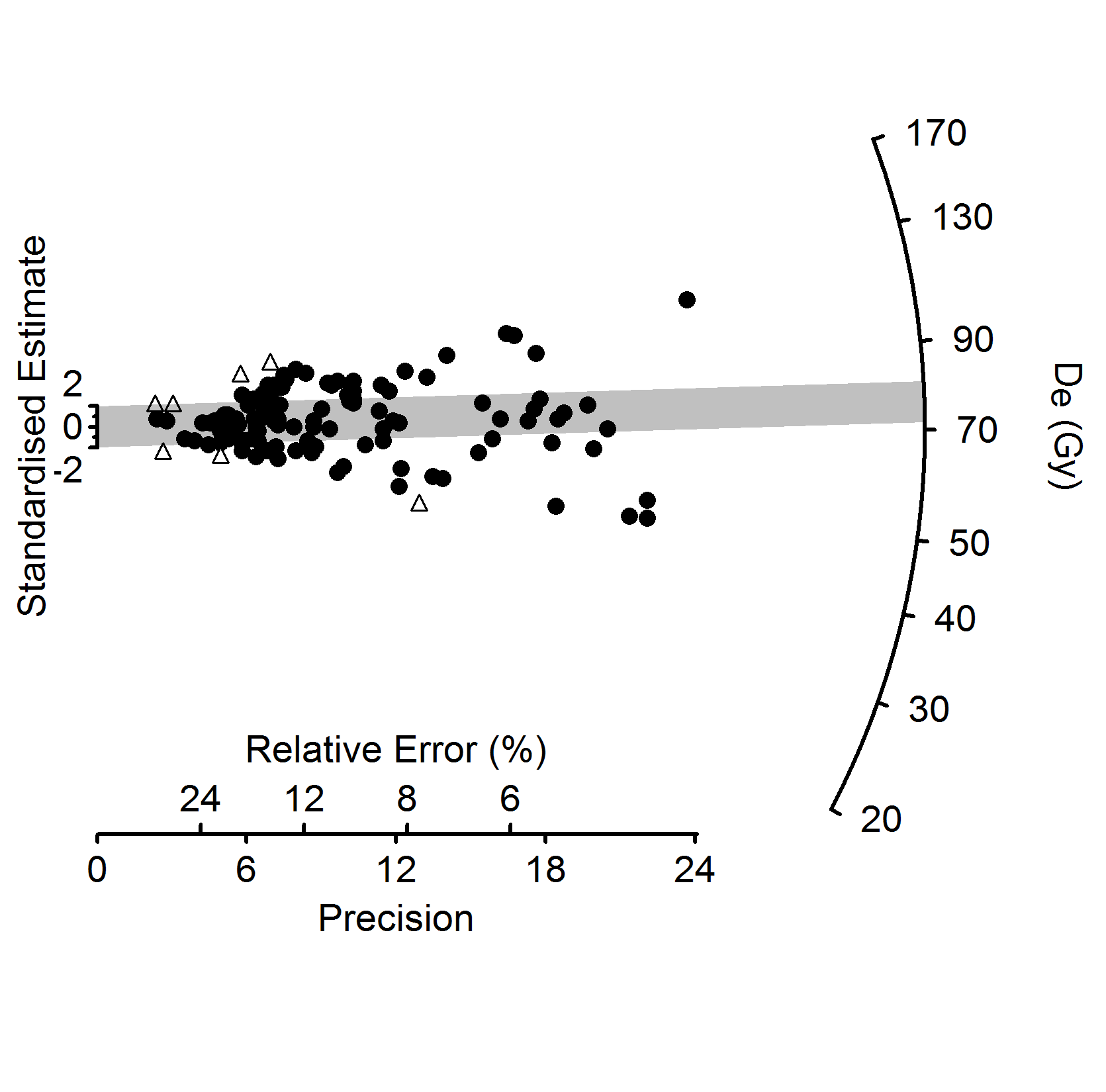

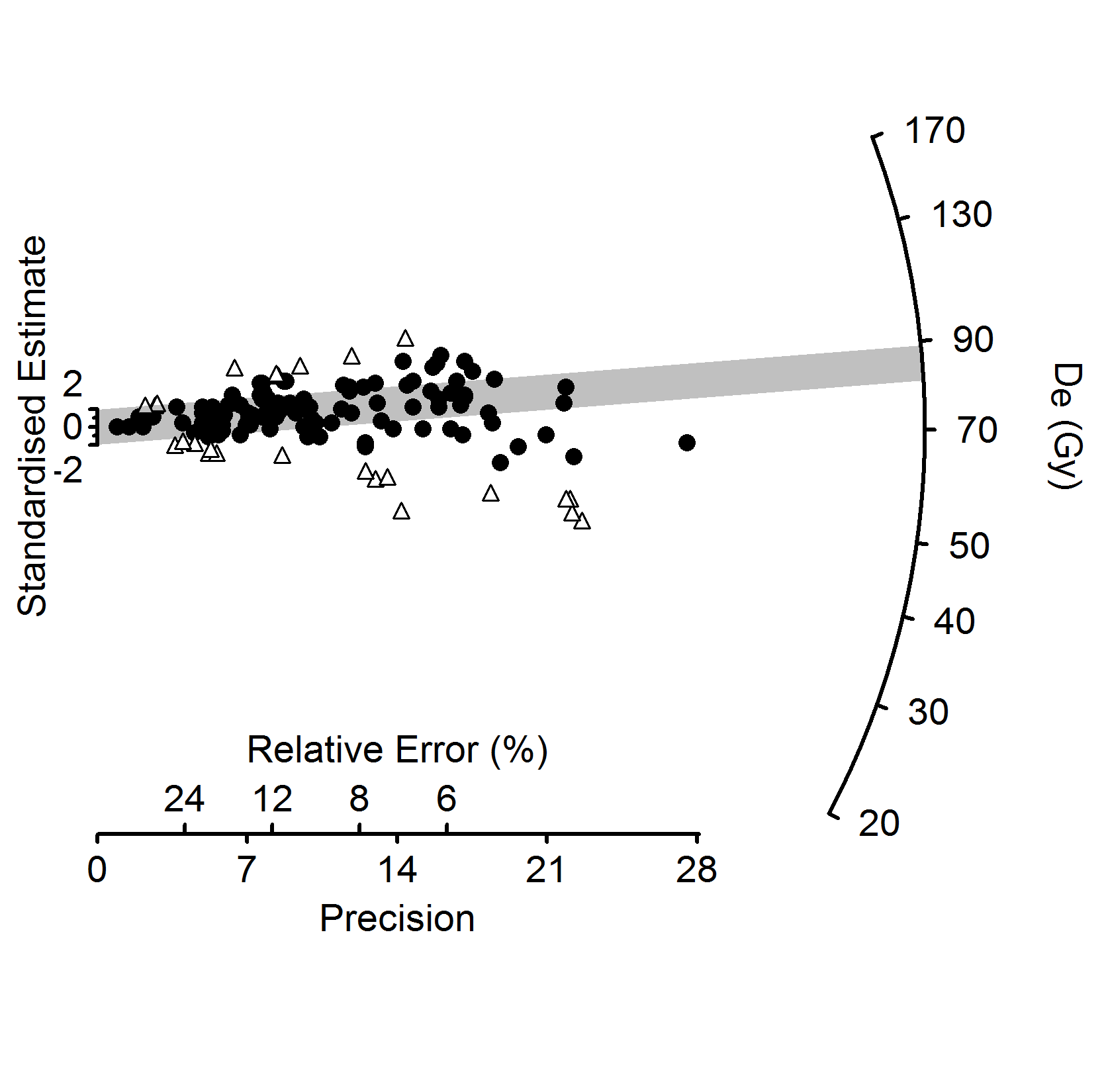


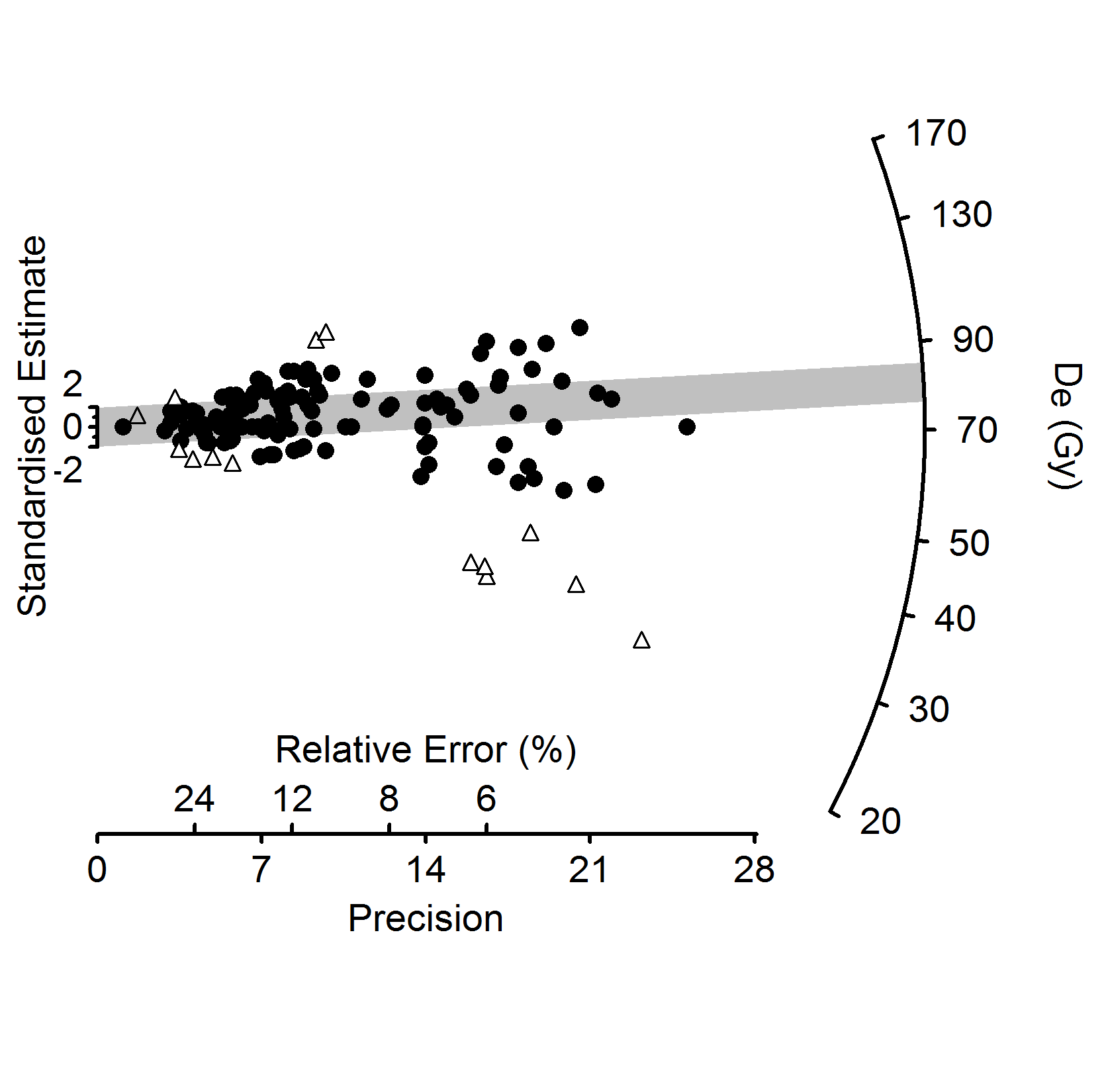


Riwi-37

N = 128

D_e_ = 80.2 ± 2.6

OD = 38 ± 3
