# Supplementary material for: Towards an Accurate and Precise Chronology for the Colonization of Australia: The Example of Riwi, Kimberley, Western Australia
Source: PLoS One. 2016 Sep 21;11(9):e0160123. doi: 10.1371/journal.pone.0160123 (PMC5031455; doi:10.1371/journal.pone.0160123)
Supplement: S1 Table — (DOCX) [file pone.0160123.s002.docx]

| **Supplementary Information**  **Towards an accurate and precise chronology for the colonization of Australia: The example of Riwi, Kimberly, Western Australia**  Wood, R.^1*^, Jacobs, Z.^2^, Balme, J.^3^, O’Connor, S.^4^, Vannieuwenhuyse, D.^3^, Whitau, R.^4^  *^1^Research School of Earth Sciences, Australian National University, Canberra, 2601, Australia*  *^2^Centre for Archaeological Science, School of Earth and Environmental Sciences, University of Wollongong, 2522, Australia*  *^3^School of Social Sciences, University of Western Australia, Crawley, 6009, Australia*  *^4^Department of Archaeology and Natural History, Research School of Pacific and Asian Studies, Australian National University, Canberra, 2601, Australia*  **S1 Table: Number of single-grains measured, rejected and accepted, together with the reasons for their rejection.** | | | | | | | | |
| --- | --- | --- | --- | --- | --- | --- | --- | --- |
| **Sample name** | **No. of grains measured** | **T_N_ signal <3xBG** | **0 Gy dose >5% of L_N_** | **Poor recycling ratio** | **No L_N_/T_N_ intersection** | **Depletion by IR** | **Sum of rejected grains** | **Acceptable individual D_e_ values** |
| **Riwi-1** | 500 | 203 | 8 | 26 | 28 | 8 | 273 | 227 |
| **Riwi-2** | 500 | 240 | 28 | 30 | 7 | 10 | 315 | 185 |
| **Riwi-3** | 500 | 247 | 12 | 32 | 22 | 8 | 321 | 179 |
| **Riwi-4** | 500 | 160 | 27 | 33 | 0 | 12 | 232 | 268 |
| **Riwi-5** | 500 | 146 | 42 | 64 | 0 | 14 | 266 | 234 |
| **Riwi-6** | 500 | 212 | 17 | 40 | 7 | 7 | 283 | 217 |
| **Riwi-7** | 500 | 223 | 10 | 19 | 22 | 6 | 289 | 211 |
| **Riwi-8** | 500 | 235 | 10 | 22 | 16 | 5 | 288 | 212 |
| **Riwi-9** | 500 | 238 | 3 | 37 | 26 | 11 | 315 | 185 |
| **Riwi-10** | 500 | 248 | 7 | 35 | 16 | 11 | 317 | 183 |
| **Riwi-11** | 500 | 222 | 7 | 27 | 30 | 7 | 293 | 207 |
| **Riwi-12** | 500 | 246 | 8 | 22 | 16 | 6 | 298 | 202 |
| **Riwi-13** | 500 | 230 | 3 | 30 | 29 | 10 | 302 | 198 |
| **Riwi-14** | 500 | 243 | 6 | 11 | 10 | 10 | 280 | 220 |
| **Riwi-15** | 500 | 245 | 6 | 37 | 17 | 12 | 317 | 183 |
| **Riwi-16** | 500 | 259 | 5 | 20 | 23 | 11 | 318 | 182 |
| **Riwi-17** | 500 | 247 | 10 | 25 | 40 | 5 | 327 | 173 |
| **Riwi-18** | 500 | 224 | 9 | 17 | 39 | 26 | 315 | 185 |
| **Riwi-19** | 500 | 211 | 9 | 20 | 39 | 7 | 286 | 214 |
| **Riwi-20** | 500 | 186 | 7 | 55 | 32 | 17 | 297 | 203 |
| **Riwi-21** | 500 | 245 | 7 | 21 | 45 | 8 | 326 | 174 |
| **Riwi-22** | 500 | 264 | 6 | 18 | 26 | 8 | 322 | 178 |
| **Riwi-23** | 500 | 216 | 7 | 30 | 35 | 4 | 292 | 208 |
| **Riwi-24** | 500 | 151 | 1 | 53 | 34 | 3 | 242 | 258 |
| **Riwi-25** | 500 | 186 | 0 | 59 | 35 | 3 | 283 | 217 |
| **Riwi-26** | 500 | 311 | 1 | 23 | 26 | 4 | 365 | 135 |
| **Riwi-27** | 500 | 298 | 1 | 22 | 31 | 6 | 358 | 142 |
| **Riwi-28** | 500 | 290 | 0 | 23 | 40 | 6 | 359 | 141 |
| **Riwi-29** | 500 | 297 | 8 | 26 | 25 | 14 | 370 | 130 |
| **Riwi-30** | 500 | 274 | 11 | 26 | 25 | 6 | 342 | 158 |
| **Riwi-31** | 500 | 272 | 6 | 25 | 27 | 10 | 340 | 160 |
| **Riwi-32** | 500 | 280 | 10 | 24 | 22 | 8 | 344 | 156 |
| **Riwi-33** | 500 | 307 | 5 | 27 | 17 | 8 | 364 | 136 |
| **Riwi-34** | 500 | 286 | 8 | 31 | 29 | 6 | 360 | 140 |
| **Riwi-35** | 500 | 330 | 1 | 25 | 20 | 7 | 383 | 117 |
| **Riwi-36** | 500 | 325 | 2 | 13 | 24 | 6 | 370 | 130 |
| **Riwi-37** | 500 | 312 | 0 | 30 | 26 | 4 | 372 | 128 |
| **Total** | 18500 | 9109 | 308 | 1078 | 906 | 314 | 11724 | 6776 |
| ***% total*** |  | ***49.2*** | ***1.7*** | ***5.8*** | ***4.9*** | ***1.7*** | ***63.4*** | ***36.6*** |
| ***% accept*** |  |  | ***3.3*** | ***11.5*** | ***9.6*** | ***3.3*** |  | ***72.2*** |

T_N_ is the OSL signal measured in response to the test dose given after measurement of the natural OSL signal.

L_N_ is the natural OSL signal.

Recycling ratio is the ratio of the sensitivity-corrected OSL signals measured from duplicate doses to test the efficacy of the test dose correction used in the SAR procedure.

IR is the infrared stimulation used to erase any part of the signal that may be derived from IR-sensitive (e.g., feldspar) grains.
