# Supplementary material for: Towards an Accurate and Precise Chronology for the Colonization of Australia: The Example of Riwi, Kimberley, Western Australia
Source: PLoS One. 2016 Sep 21;11(9):e0160123. doi: 10.1371/journal.pone.0160123 (PMC5031455; doi:10.1371/journal.pone.0160123)
Supplement: S2 Table — (DOCX) [file pone.0160123.s003.docx]

| **Supplementary Information**  **Towards an accurate and precise chronology for the colonization of Australia: The example of Riwi, Kimberly, Western Australia**  Wood, R.^1*^, Jacobs, Z.^2^, Balme, J.^3^, O’Connor, S.^4^, Vannieuwenhuyse, D.^3^, Whitau, R.^4^  *^1^Research School of Earth Sciences, Australian National University, Canberra, 2601, Australia*  *^2^Centre for Archaeological Science, School of Earth and Environmental Sciences, University of Wollongong, 2522, Australia*  *^3^School of Social Sciences, University of Western Australia, Crawley, 6009, Australia*  *^4^Department of Archaeology and Natural History, Research School of Pacific and Asian Studies, Australian National University, Canberra, 2601, Australia*  **S2 Table: Fitting details, D_e_ values for each component and the proportion of grains in each component for the two samples with mixed D_e_ distributions for which the finite mixture model was use.**   \| **Sample** \| **OD (%)** \| **k** \| **D_e_-1 (Gy)** \| **Proportion (%)** \| **D_e_-2 (Gy)** \| **Proportion (%)** \| **D_e_-3 (Gy)** \| **Proportion (%)** \| **D_e_-4 (Gy)** \| **Proportion (%)** \| \| --- \| --- \| --- \| --- \| --- \| --- \| --- \| --- \| --- \| --- \| --- \| \| **Riwi-2** \| 25 \| 4 \| 49.9 ± 1.7 \| 53 ± 4 \| 15.3 ± 0.8 \| 27 ± 3 \| 4.8 ± 0.5 \| 9 ± 2 \| 1.7 ± 0.1 \| 11 ± 3 \| \| **Riwi-6** \| 15 \| 2 \| 46.1 ± 0.8 \| 59 ± 3 \| 12.7 ± 0.3 \| 41 ± 3 \|  \|  \|  \|  \| |
| --- | --- | --- | --- | --- | --- | --- | --- | --- | --- | --- | --- | --- | --- | --- | --- | --- | --- | --- | --- | --- | --- | --- | --- | --- | --- | --- | --- | --- | --- | --- | --- | --- | --- |
