# Supplementary material for: Towards an Accurate and Precise Chronology for the Colonization of Australia: The Example of Riwi, Kimberley, Western Australia
Source: PLoS One. 2016 Sep 21;11(9):e0160123. doi: 10.1371/journal.pone.0160123 (PMC5031455; doi:10.1371/journal.pone.0160123)
Supplement: S3 Table — Weighted mean De values and overdispersion values for each sample following two different scenarios: (a) including all De values in each samples, or (b) rejecting outlier values identified as log De values with normalised median absolute deviations (nMADs) greater than 1.5. Also provided in the final column is the ratio of the latter over the former. (DOCX) [file pone.0160123.s004.docx]

| **Supplementary Information**  **Towards an accurate and precise chronology for the colonization of Australia: The example of Riwi, Kimberly, Western Australia**  Wood, R.^1*^, Jacobs, Z.^2^, Balme, J.^3^, O’Connor, S.^4^, Vannieuwenhuyse, D.^3^, Whitau, R.^4^  *^1^Research School of Earth Sciences, Australian National University, Canberra, 2601, Australia*  *^2^Centre for Archaeological Science, School of Earth and Environmental Sciences, University of Wollongong, 2522, Australia*  *^3^School of Social Sciences, University of Western Australia, Crawley, 6009, Australia*  *^4^Department of Archaeology and Natural History, Research School of Pacific and Asian Studies, Australian National University, Canberra, 2601, Australia* |
| --- |

**S3 Table: Weighted mean De values and overdispersion values for each sample following two different scenarios**: (a) including all De values in each samples, or (b) rejecting outlier values identified as log De values with normalised median absolute deviations (nMADs) greater than 1.5. Also provided in the final column is the ratio of the latter over the former.

| **Sample** | **All values included** | | **After outlier rejection** | | **D_e_ ratios** |
| --- | --- | --- | --- | --- | --- |
|  | **D_e_ (Gy)** | **OD (%)** | **D_e_ (Gy)** | **OD (%)** |  |
| Riwi-4 | 13.1 ± 0.3 | 30.4 ± 1.4 | 13.0 ± 0.1 | 13.0 ± 0.8 | 0.99 ± 0.02 |
| Riwi-5 | 12.1 ± 0.3 | 35.0 ± 1.7 | 11.7 ± 0.2 | 18.1 ± 1.1 | 0.97 ± 0.03 |
| Riwi-1 | 54.0 ± 1.3 | 32.3 ± 1.8 | 57.8 ± 1.0 | 19.2 ± 1.4 | 1.07 ± 0.03 |
| Riwi-3 | 48.1 ± 2.4 | 65.2 ± 3.7 | 57.4 ± 1.4 | 25.7 ± 1.9 | 1.19 ± 0.07 |
| Riwi-7 | 63.0 ± 1.6 | 32.5 ± 1.9 | 63.3 ± 1.2 | 20.5 ± 1.5 | 1.00 ± 0.03 |
| Riwi-8 | 65.4 ± 1.6 | 31.5 ± 1.9 | 67.0 ± 1.1 | 17.3 ± 1.4 | 1.02 ± 0.03 |
| Riwi-9 | 60.5 ± 1.8 | 36.0 ± 2.2 | 64.1 ± 1.4 | 23.1 ± 1.7 | 1.06 ± 0.04 |
| Riwi-10 | 68.3 ± 1.7 | 30.3 ± 2.0 | 69.3 ± 1.4 | 21.5 ± 1.7 | 1.02 ± 0.03 |
| Riwi-11 | 67.4 ± 1.6 | 28.6 ± 1.8 | 68.0 ± 1.4 | 22.9 ± 1.6 | 1.01 ± 0.03 |
| Riwi-12 | 67.2 ± 1.7 | 30.9 ± 1.9 | 68.9 ± 1.3 | 19.2 ± 1.5 | 1.03 ± 0.03 |
| Riwi-13 | 72.1 ± 2.2 | 37.3 ± 2.3 | 72.9 ± 1.9 | 29.3 ± 2.0 | 1.01 ± 0.04 |
| Riwi-14 | 67.4 ± 1.8 | 35.4 ± 2.0 | 71.4 ± 1.2 | 17.9 ± 1.4 | 1.06 ± 0.03 |
| Riwi-15 | 66.3 ± 1.9 | 34.9 ± 2.3 | 68.6 ± 1.6 | 23.7 ± 1.8 | 1.04 ± 0.04 |
| Riwi-16 | 66.1 ± 1.7 | 30.9 ± 2.0 | 68.2 ± 1.3 | 18.8 ± 1.5 | 1.03 ± 0.03 |
| Riwi-17 | 72.6 ± 2.1 | 33.7 ± 2.2 | 77.4 ± 1.8 | 22.6 ± 1.9 | 1.07 ± 0.04 |
| Riwi-18 | 69.3 ± 1.8 | 29.9 ± 2.0 | 70.9 ± 1.5 | 21.9 ± 1.7 | 1.02 ± 0.03 |
| Riwi-19 | 69.6 ± 1.7 | 31.5 ± 1.9 | 72.3 ± 1.5 | 23.5 ± 1.7 | 1.04 ± 0.03 |
| Riwi-20 | 66.8 ± 1.7 | 32.4 ± 1.9 | 69.2 ± 1.4 | 24.5 ± 1.6 | 1.03 ± 0.03 |
| Riwi-21 | 71.2 ± 2.2 | 35.7 ± 2.3 | 73.0 ± 2.2 | 19.7 ± 1.7 | 1.02 ± 0.04 |
| Riwi-22 | 77.5 ± 1.8 | 29.7 ± 2.0 | 76.2 ± 1.9 | 25.9 ± 1.8 | 0.98 ± 0.03 |
| Riwi-23 | 77.6 ± 1.4 | 30.4 ± 1.9 | 73.4 ± 1.8 | 18.6 ± 1.5 | 0.95 ± 0.03 |
| Riwi-24 | 66.3 ± 1.6 | 34.6 ± 1.8 | 67.7 ± 1.2 | 23.2 ± 1.4 | 1.02 ± 0.03 |
| Riwi-25 | 71.9 ± 1.6 | 29.7 ± 1.6 | 72.9 ± 1.2 | 19.1 ± 1.3 | 1.01 ± 0.03 |
| Riwi-26 | 65.8 ± 1.7 | 27.5 ± 2.0 | 66.2 ± 1.3 | 18.0 ± 1.6 | 1.01 ± 0.03 |
| Riwi-27 | 68.8 ± 1.8 | 28.5 ± 2.0 | 68.2 ± 1.5 | 20.7 ± 1.7 | 0.99 ± 0.03 |
| Riwi-28 | 73.0 ± 2.3 | 34.9 ± 2.3 | 71.6 ± 1.8 | 26.1 ± 2.0 | 0.98 ± 0.04 |
| Riwi-29 | 74.3 ± 2.7 | 37.4 ± 2.7 | 76.8 ± 2.1 | 25.3 ± 2.2 | 1.03 ± 0.05 |
| Riwi-30 | 72.7 ± 2.0 | 30.9 ± 2.1 | 74.0 ± 1.5 | 18.6 ± 1.6 | 1.02 ± 0.03 |
| Riwi-31 | 74.4 ± 2.0 | 31.3 ± 2.1 | 76.6 ± 1.6 | 21.0 ± 1.7 | 1.03 ± 0.04 |
| Riwi-32 | 74.9 ± 1.8 | 25.8 ± 1.9 | 73.3 ± 1.5 | 19.0 ± 1.7 | 0.98 ± 0.03 |
| Riwi-33 | 79.8 ± 2.6 | 34.5 ± 2.5 | 78.5 ± 1.9 | 22.4 ± 1.9 | 0.98 ± 0.04 |
| Riwi-34 | 68.6 ± 2.7 | 43.2 ± 2.9 | 69.4 ± 1.9 | 26.4 ± 2.2 | 1.01 ± 0.05 |
| Riwi-35 | 76.2 ± 2.5 | 32.1 ± 2.5 | 75.8 ± 2.3 | 29.3 ± 2.3 | 0.99 ± 0.04 |
| Riwi-36 | 81.1 ± 2.3 | 29.9 ± 2.2 | 84.6 ± 1.8 | 17.8 ± 1.7 | 1.04 ± 0.04 |
| Riwi-37 | 76.2 ± 2.7 | 37.9 ± 2.7 | 80.2 ± 2.3 | 27.1 ± 2.2 | 1.05 ± 0.05 |
