# Supplementary material for: Towards an Accurate and Precise Chronology for the Colonization of Australia: The Example of Riwi, Kimberley, Western Australia
Source: PLoS One. 2016 Sep 21;11(9):e0160123. doi: 10.1371/journal.pone.0160123 (PMC5031455; doi:10.1371/journal.pone.0160123)
Supplement: S4 Table — Where dates have not been included in the model, no Modelled probability age range is given. (DOCX) [file pone.0160123.s005.docx]

| **Supplementary Information**  **Towards an accurate and precise chronology for the colonization of Australia: The example of Riwi, Kimberly, Western Australia**  Wood, R.^1*^, Jacobs, Z.^2^, Balme, J.^3^, O’Connor, S.^4^, Vannieuwenhuyse, D.^3^, Whitau, R.^4^  *^1^Research School of Earth Sciences, Australian National University, Canberra, 2601, Australia*  *^2^Centre for Archaeological Science, School of Earth and Environmental Sciences, University of Wollongong, 2522, Australia*  *^3^School of Social Sciences, University of Western Australia, Crawley, 6009, Australia*  *^4^Department of Archaeology and Natural History, Research School of Pacific and Asian Studies, Australian National University, Canberra, 2601, Australia* |
| --- |

**S4 Table: Bayesian model of radiocarbon dates**. Where dates have not been included in the model, no Modelled probability age range is given.

| Name | Unmodelled (cal BP) | | | | | | | | Modelled (cal BP) | | | | | | | | | C | |
| --- | --- | --- | --- | --- | --- | --- | --- | --- | --- | --- | --- | --- | --- | --- | --- | --- | --- | --- | --- |
|  | 68.2% probability range | | | | 95.4% probability range | | | | 68.2% probability range | | | | 95.4% probability range | | | | |  | |
|  | from | | to | | from | | to | | from | | to | | from | | to | | |  |  |
| Boundary End 1 |  | | | | | | | | 640 | | 385 | | 660 | | -580 | | | 94.7 | |
| R_Date SANU43337 | 640 | | 560 | | 650 | | 555 | | 650 | | 565 | | 655 | | 555 | | | 99.2 | |
| R_Date D-AMS 004068 | 720 | | 675 | | 730 | | 670 | | 720 | | 675 | | 735 | | 665 | | | 99.9 | |
| R_Date D-AMS 004064 | 905 | | 770 | | 915 | | 760 | | 900 | | 765 | | 910 | | 740 | | | 99.4 | |
| R_Date SANU-38221 | 7415 | | 7275 | | 7420 | | 7270 | |  | |  | |  | |  | | | 99.9 | |
| R_Date SANU-39505 | 7320 | | 7180 | | 7415 | | 7170 | |  | |  | |  | |  | | | 99.9 | |
| R_Date SANU-38220 | 22855 | | 22625 | | 22960 | | 22525 | |  | |  | |  | |  | | | 99.8 | |
| Phase 1 |  | | | | | | | | | | | | | | | | | | |
| Boundary Start 1 |  | | | | | | | | 1150 | | 765 | | 2530 | | 700 | | 97 | | |
| Boundary End 2 |  | | | | | | | | 7125 | | 6865 | | 7155 | | 5585 | | 79.8 | | |
| R_Date D-AMS 004067 | 7415 | | 7305 | | 7420 | | 7270 | | 7345 | | 7265 | | 7420 | | 7180 | | 96.5 | | |
| R_Date D-AMS 004062 | 7250 | | 7170 | | 7275 | | 7025 | | 7250 | | 7175 | | 7275 | | 7030 | | 99.9 | | |
| R_Date D-AMS 004061 | 7170 | | 7015 | | 7245 | | 7000 | | 7175 | | 7025 | | 7245 | | 7010 | | 99.5 | | |
| R_Date Wk 7605 | 6175 | | 5925 | | 6195 | | 5905 | | 7335 | | 6155 | | 7430 | | 5915 | | 65.1 | | |
| R_Date D-AMS 004069 | 7155 | | 6950 | | 7160 | | 6935 | | 7160 | | 6995 | | 7165 | | 6950 | | 97.9 | | |
| R_Date D-AMS 004065 | 7155 | | 7005 | | 7175 | | 6935 | | 7160 | | 7045 | | 7235 | | 6950 | | 98.8 | | |
| R_Date D-AMS 004063 | 7320 | | 7180 | | 7415 | | 7170 | | 7315 | | 7180 | | 7405 | | 7165 | | 99.5 | | |
| R_Date SANU-38223 | 7170 | | 7020 | | 7245 | | 7000 | | 7170 | | 7025 | | 7245 | | 7005 | | 99.5 | | |
| Phase 2 |  | | | | | | | | | | | | | | | | | | |
| Boundary Start 2 |  | | | | | | | | 7430 | | 7280 | | 7685 | | 7195 | | 91.6 | | |
| Sequence_Holocene | | | | | | | | | | | | | | | | | | | |
| Boundary End 3 | |  | | | | | | | | 20440 | | 19150 | | 20570 | | 16470 | | | 84.1 |
| R_Date SANU-38225 | | 22370 | | 22130 | | 22420 | | 21960 | | 22370 | | 22100 | | 22470 | | 21860 | | | 92.5 |
| R_Combine SANU-38226 38814 | | 20460 | | 20250 | | 20540 | | 20140 | | 20480 | | 20260 | | 20620 | | 20040 | | | 95.7 |
| Phase 3 | |  | | | | | | | | | | | | | | | | | |
| Boundary Start 3 | |  | | | | | | | | 24880 | | 22090 | | 29140 | | 20440 | | | 75.3 |
| Boundary End 4 | |  | | | | | | | | 31320 | | 28730 | | 31390 | | 24690 | | | 95.5 |
| R_Date SANU-39509 | | 31280 | | 31060 | | 31400 | | 30940 | | 31280 | | 31040 | | 31470 | | 30830 | | | 98.7 |
| Phase 4 | |  | | | | | | | | | | | | | | | | | |
| Boundary Start 4 | |  | | | | | | | | 31840 | | 31090 | | 32800 | | 30930 | | | 97.7 |
| Boundary End 5 | |  | | | | | | | | 32830 | | 31610 | | 33330 | | 31230 | | | 98.5 |
| R_Date SANU-39507 | | 34810 | | 34370 | | 35010 | | 34140 | | 33210 | | 32120 | | 33520 | | 31520 | | | 98.0 |
| Phase 5 | |  | | | | | | | | | | | | | | | | | |
| Boundary Start 5 | |  | | | | | | | | 33480 | | 32510 | | 33710 | | 31790 | | | 98.7 |
| Boundary End 6 | |  | | | | | | | | 33690 | | 33210 | | 33970 | | 32830 | | | 99.4 |
| R_Date Wk 7896 | | 33970 | | 33440 | | 34200 | | 33030 | | 33970 | | 33570 | | 34180 | | 33350 | | | 99.8 |
| R_Date D-AMS 004070 | | 34300 | | 33980 | | 34490 | | 33850 | | 34200 | | 33910 | | 34370 | | 33780 | | | 99.8 |
| R_Date SANU-35920 | | 34310 | | 33910 | | 34540 | | 33770 | | 34180 | | 33860 | | 34380 | | 33720 | | | 99.7 |
| R_Date SANU-35907 | | 34040 | | 33710 | | 34230 | | 33560 | | 34030 | | 33730 | | 34180 | | 33580 | | | 99.9 |
| R_Combine SANU-39506 39510 | | 33610 | | 33220 | | 33730 | | 32980 | | 33750 | | 33400 | | 34040 | | 33170 | | | 99.5 |
| Phase 6 | |  | | | | | | | | | | | | | | | | | |
| Boundary Start 6 | |  | | | | | | | | 34460 | | 34000 | | 34920 | | 33850 | | | 99.3 |
| Boundary End 7 | |  | | | | | | | | 35860 | | 34930 | | 36040 | | 34130 | | | 98.5 |
| R_Date Wk 7606 | | 36190 | | 35230 | | 36730 | | 34770 | | 36210 | | 35460 | | 36650 | | 35050 | | | 99.0 |
| R_Date D-AMS 004066 | | 35980 | | 35550 | | 36160 | | 35340 | | 36010 | | 35590 | | 36200 | | 35360 | | | 99.4 |
| R_Date SANU-35916 | | 33990 | | 33670 | | 34170 | | 33500 | | 36550 | | 35370 | | 37160 | | 34030 | | | 89.6 |
| R_Date SANU-37707 | | 39290 | | 38540 | | 39780 | | 38290 | | 36710 | | 35500 | | 37370 | | 34960 | | | 98.2 |
| R_Combine SANU-35914 35921 35924 | | 37440 | | 36700 | | 37830 | | 36480 | | 37120 | | 35650 | | 37360 | | 35260 | | | 98.1 |
| Phase 7 | |  | | | | | | | | | | | | | | | | | |
| Boundary Start 7 | |  | | | | | | | | 37410 | | 35840 | | 37670 | | 35590 | | | 97.6 |
| Boundary End 8 | |  | | | | | | | | 37810 | | 36800 | | 38130 | | 36010 | | | 99 |
| Phase 8 | |  | | | | | | | | | | | | | | | | | |
| Boundary Start 8 | |  | | | | | | | | 38170 | | 37300 | | 38430 | | 36620 | | | 99.6 |
| Boundary End 9 | |  | | | | | | | | 38420 | | 37840 | | 38600 | | 37360 | | | 99.6 |
| R_Date SANU-35913 | | 38670 | | 37880 | | 38960 | | 37200 | | 38580 | | 38100 | | 38780 | | 37740 | | | 99.6 |
| R_Date SANU-35919 | | 38840 | | 38090 | | 39230 | | 37460 | | 38610 | | 38120 | | 38850 | | 37740 | | | 99.6 |
| R_Date SANU-35906 | | 38410 | | 37450 | | 38610 | | 36850 | | 38530 | | 38050 | | 38710 | | 37680 | | | 99.7 |
| Phase 9 | |  | | | | | | | | | | | | | | | | | |
| Boundary Start 9 | |  | | | | | | | | 38860 | | 38190 | | 39590 | | 37790 | | | 98.9 |
| Boundary End 10 | |  | | | | | | | | 41240 | | 39180 | | 41530 | | 38130 | | | 94.1 |
| R_Date SANU-35918 | | 38090 | | 37050 | | 38410 | | 36650 | | 41870 | | 39910 | | 42490 | | 38140 | | | 92.4 |
| R_Date SANU-37706 | | 41670 | | 40870 | | 41970 | | 40400 | | 41620 | | 40760 | | 41990 | | 40230 | | | 99.0 |
| Phase 10 | |  | | | | | | | | | | | | | | | | | |
| Boundary Start 10 | |  | | | | | | | | 42650 | | 40940 | | 44060 | | 40490 | | | 98.7 |
| Boundary End 11 | |  | | | | | | | | 44770 | | 43470 | | 45080 | | 42110 | | | 97.6 |
| R_Combine SANU-35911 and 35922 | | 45450 | | 44500 | | 45990 | | 44030 | | 44870 | | 44150 | | 45210 | | 43650 | | | 99.4 |
| R_Date SANU-35910 | | 34080 | | 33750 | | 34270 | | 33590 | | 44890 | | 43890 | | 45260 | | 42830 | | | 98.4 |
| R_Date ANUA-13005 | | 45680 | | 43810 | | 46900 | | 43040 | | 44860 | | 44010 | | 45180 | | 43410 | | | 99.4 |
| Phase 11 | |  | | | | | | | | | | | | | | | | | |
| Boundary Start 11 | |  | | | | | | | | 45060 | | 44340 | | 45420 | | 43900 | | | 99.3 |
| Boundary End 12 top | |  | | | | | | | | 45330 | | 44640 | | 45690 | | 44270 | | | 99.5 |
| R_Combine SANU-35917 and 35925 | | 45470 | | 44530 | | 46000 | | 44060 | | 45490 | | 44810 | | 45860 | | 44500 | | | 99.8 |
| R_Date SANU-35909 | | 45680 | | 44330 | | 46480 | | 43600 | | 45500 | | 44800 | | 45900 | | 44470 | | | 99.7 |
| Phase 12 top | |  | | | | | | | | | | | | | | | | | |
| Boundary Transition 12 mid/ 12 top | |  | | | | | | | | 45720 | | 44910 | | 46350 | | 44560 | | | 99.6 |
| R_Date ANUA-13006 | | 45350 | | 43160 | | 47070 | | 42420 | | 46130 | | 45070 | | 47130 | | 44680 | | | 99.7 |
| Phase 12 mid | |  | | | | | | | | | | | | | | | | | |
| Boundary Start 12 mid | |  | | | | | | | | 46880 | | 45130 | | 48990 | | 44730 | | | 95.1 |
| Sequence Pleistocene | |  | | | | | | | | | | | | | | | | | |
