# Supplementary material for: Towards an Accurate and Precise Chronology for the Colonization of Australia: The Example of Riwi, Kimberley, Western Australia
Source: PLoS One. 2016 Sep 21;11(9):e0160123. doi: 10.1371/journal.pone.0160123 (PMC5031455; doi:10.1371/journal.pone.0160123)
Supplement: S5 Table — Dates are in years before 1950 to enable comparison with radiocarbon age estimates. (DOCX) [file pone.0160123.s006.docx]

| **Supplementary Information**  **Towards an accurate and precise chronology for the colonization of Australia: The example of Riwi, Kimberly, Western Australia**  Wood, R.^1*^, Jacobs, Z.^2^, Balme, J.^3^, O’Connor, S.^4^, Vannieuwenhuyse, D.^3^, Whitau, R.^4^  *^1^Research School of Earth Sciences, Australian National University, Canberra, 2601, Australia*  *^2^Centre for Archaeological Science, School of Earth and Environmental Sciences, University of Wollongong, 2522, Australia*  *^3^School of Social Sciences, University of Western Australia, Crawley, 6009, Australia*  *^4^Department of Archaeology and Natural History, Research School of Pacific and Asian Studies, Australian National University, Canberra, 2601, Australia* |
| --- |

**S5 Table: Bayesian model of OSL dates, including random error only**. Dates are in years before 1950 to enable comparison with radiocarbon age estimates.

| Name | | Unmodelled (yrs) | | | | Modelled (yrs) | | | | C |
| --- | --- | --- | --- | --- | --- | --- | --- | --- | --- | --- |
|  |  | 68.2% probability range | | 95.4% probability range | | 68.2% probability range | | 95.4% probability range | |  |
|  |  | from | to | from | to | from | to | from | to |  |
| Boundary End 6 |  | | | | | 34640 | 32720 | 35500 | 31140 | 93.9 |
| C_Date Riwi-1 | | 35390 | 33670 | 36250 | 32820 | 34750 | 33300 | 35470 | 32530 | 99.6 |
| Sequence 6 |  | |  |  |  |  |  |  |  |  |
| Boundary Start 6 |  | | | | | 35100 | 33610 | 35830 | 32810 | 99.6 |
| Boundary End 7 |  | | | | | 35650 | 34150 | 36390 | 33370 | 99.6 |
| C_Date Riwi-3 | | 34140 | 31990 | 35210 | 30920 | 35890 | 34530 | 36560 | 33790 | 99.7 |
| C_Date Riwi-7 | | 36170 | 34340 | 37080 | 33430 | 36360 | 35150 | 37000 | 34540 | 99.8 |
| C_Date Riwi-8 | | 38690 | 36790 | 39630 | 35850 | 37310 | 35850 | 38070 | 35210 | 99.7 |
| Sequence 7 |  | | | | | | | | | |
| Boundary Start 7 |  | | | | | 37960 | 36170 | 38750 | 35400 | 99.4 |
| Boundary End 8 |  | | | | | 39130 | 37720 | 39660 | 36820 | 99.1 |
| C_Date Riwi-9 | | 39010 | 36840 | 40090 | 35760 | 39200 | 38030 | 39730 | 37370 | 99.8 |
| C_Date Riwi-10 | | 41910 | 39630 | 43050 | 38500 | 39390 | 38340 | 39890 | 37800 | 99.8 |
| C_Date Riwi-11 | | 41240 | 39050 | 42340 | 37950 | 39570 | 38520 | 40070 | 37980 | 99.8 |
| Sequence 8 |  | | | | | | | | | |
| Boundary Start 8 |  | | | | | 39740 | 38640 | 40270 | 38090 | 99.6 |
| Boundary End 9 |  | | | | | 40140 | 39040 | 40660 | 38480 | 99.7 |
| C_Date Riwi-12 | | 39980 | 37970 | 40980 | 36970 | 40320 | 39270 | 40830 | 38740 | 99.8 |
| Sequence 9 |  | | | | | | | | | |
| Boundary Start 9 |  | | | | | 40620 | 39510 | 41120 | 38950 | 99.7 |
| Boundary End 10 |  | | | | | 41130 | 40090 | 41580 | 39540 | 99.4 |
| C_Date Riwi-13 | | 41320 | 38810 | 42580 | 37550 | 41170 | 40220 | 41630 | 39730 | 99.8 |
| C_Date Riwi-14 | | 41910 | 39860 | 42930 | 38840 | 41240 | 40360 | 41680 | 39910 | 99.8 |
| C_Date Riwi-15 | | 41060 | 38720 | 42230 | 37560 | 41310 | 40480 | 41750 | 40060 | 99.8 |
| C_Date Riwi-16 | | 39310 | 37310 | 40310 | 36320 | 41410 | 40600 | 41840 | 40200 | 99.8 |
| C_Date Riwi-17 | | 45580 | 42980 | 46870 | 41680 | 41580 | 40770 | 42000 | 40370 | 99.8 |
| C_Date Riwi-18 | | 43050 | 40710 | 44220 | 39550 | 41710 | 40880 | 42160 | 40470 | 99.8 |
| C_Date Riwi-19 | | 43980 | 41610 | 45160 | 40430 | 41850 | 40960 | 42320 | 40540 | 99.8 |
| C_Date Riwi-20 | | 42160 | 39890 | 43300 | 38750 | 41990 | 41040 | 42500 | 40590 | 99.8 |
| Sequence 10 |  | | | | | | | | | |
| Boundary Start 10 |  | | | | | 42140 | 41070 | 42700 | 40640 | 99.2 |
| Boundary End 11 |  | | | | | 42890 | 41850 | 43410 | 41340 | 99.2 |
| C_Date Riwi-21 | | 44460 | 41420 | 45970 | 39910 | 42930 | 41960 | 43460 | 41510 | 99.8 |
| C_Date Riwi-22 | | 46370 | 43550 | 47770 | 42140 | 43000 | 42070 | 43510 | 41630 | 99.8 |
| C_Date Riwi-23 | | 44330 | 41710 | 45640 | 40410 | 43080 | 42160 | 43580 | 41730 | 99.8 |
| C_Date Riwi-24 | | 41200 | 39140 | 42230 | 38110 | 43150 | 42230 | 43660 | 41810 | 99.8 |
| C_Date Riwi-25 | | 45030 | 42850 | 46120 | 41770 | 43260 | 42320 | 43790 | 41880 | 99.8 |
| C_Date Riwi-26 | | 43470 | 41160 | 44630 | 40010 | 43380 | 42380 | 43920 | 41930 | 99.7 |
| Sequence 11 |  | | | | | | | | | |
| Boundary Start 11 |  | | | | | 43490 | 42430 | 44120 | 41960 | 99.1 |
| Boundary End 12 |  | | | | | 44460 | 43070 | 45250 | 42510 | 99.5 |
| C_Date Riwi-27 | | 43560 | 41130 | 44770 | 39920 | 44720 | 43420 | 45440 | 42840 | 99.7 |
| C_Date Riwi-28 | | 44620 | 41880 | 45990 | 40510 | 45140 | 43890 | 45750 | 43310 | 99.8 |
| C_Date Riwi-29 | | 47990 | 44880 | 49530 | 43340 | 45630 | 44480 | 46190 | 43910 | 99.8 |
| C_Date Riwi-30 | | 46490 | 44020 | 47720 | 42790 | 46010 | 44920 | 46550 | 44360 | 99.8 |
| C_Date Riwi-31 | | 50120 | 47320 | 51520 | 45920 | 46440 | 45330 | 47000 | 44790 | 99.7 |
| C_Date Riwi-32 | | 46420 | 43930 | 47660 | 42690 | 46720 | 45600 | 47300 | 45040 | 99.8 |
| C_Date Riwi-33 | | 49800 | 46820 | 51280 | 45340 | 47080 | 45890 | 47720 | 45300 | 99.7 |
| C_Date Riwi-34 | | 44860 | 41930 | 46320 | 40470 | 47400 | 46120 | 48090 | 45470 | 99.6 |
| C_Date Riwi-35 | | 47990 | 44660 | 49650 | 43000 | 47950 | 46460 | 48800 | 45710 | 99.6 |
| C_Date Riwi-36 | | 52730 | 49850 | 54160 | 48410 | 48720 | 46840 | 49760 | 45900 | 99.3 |
| C_Date Riwi-37 | | 50470 | 47150 | 52120 | 45490 | 49110 | 47060 | 50280 | 46040 | 99.3 |
| Sequence 12 |  | | | | | | | | | |
| Boundary Start 12 |  | | | | | 49630 | 47030 | 50860 | 46020 | 86.2 |
| Sequence |  | | | | | | | | | |
| U(0,4) | | 3.99E-17 | 4 | 3.99E-17 | 4 | 0.164 | 3.94 | 5.38E-17 | 3.948 | 99.8 |
| T(5) | | -1.135 | 1.135 | -2.65 | 2.65 |  | | | | 99.9 |
| Outlier_Model General |  | | | | | -1051 | 2720 | -4418 | 4357 | 99.9 |
